# The Cell Wall PAC (Proline-Rich, Arabinogalactan Proteins, Conserved Cysteines) Domain-Proteins Are Conserved in the Green Lineage

**DOI:** 10.3390/ijms21072488

**Published:** 2020-04-03

**Authors:** Huan Nguyen-Kim, Hélène San Clemente, Josef Laimer, Peter Lackner, Gabriele Gadermaier, Christophe Dunand, Elisabeth Jamet

**Affiliations:** 1 Laboratoire de Recherche en Sciences Végétales, Université de Toulouse, CNRS, UPS, 31320 Auzeville Tolosane, France; huanqnu@gmail.com (H.N-K.); sancle@lrsv.ups-tlse.fr (H.S.C.); dunand@lrsv.ups-tlse.fr (C.D.); 2 Paris-Lodron-University of Salzburg, Department of Biosciences, Salzburg 5020, Austria; josef.laimer@sbg.ac.at (J.L.); peter.lackner@sbg.ac.at (P.L.); gabriele.gadermaier@sbg.ac.at (G.G.)

**Keywords:** cell wall, evolution, green lineage, modeling, PAC domain, phylogeny, plant

## Abstract

Plant cell wall proteins play major roles during plant development and in response to environmental cues. A bioinformatic search for functional domains has allowed identifying the PAC domain (Proline-rich, Arabinogalactan proteins, conserved Cysteines) in several proteins (PDPs) identified in cell wall proteomes. This domain is assumed to interact with pectic polysaccharides and *O*-glycans and to contribute to non-covalent molecular scaffolds facilitating the remodeling of polysaccharidic networks during rapid cell expansion. In this work, the characteristics of the PAC domain are described in detail, including six conserved Cys residues, their spacing, and the predicted secondary structures. Modeling has been performed based on the crystal structure of a *Plantago lanceolata* PAC domain. The presence of β-sheets is assumed to ensure the correct folding of the PAC domain as a β-barrel with loop regions. We show that PDPs are present in early divergent organisms from the green lineage and in all land plants. PAC domains are associated with other types of domains: Histidine-rich, extensin, Proline-rich, or yet uncharacterized. The earliest divergent organisms having PDPs are Bryophytes. Like the complexity of the cell walls, the number and complexity of PDPs steadily increase during the evolution of the green lineage. The association of PAC domains with other domains suggests a neo-functionalization and different types of interactions with cell wall polymers

## 1. Introduction

Plant cell walls are composite structures mainly made of polysaccharides and proteins. Cellulose microfibrils and hemicelluloses form intricate networks, which are embedded in a pectin matrix [1]. Although present in minor amounts, the cell wall proteins (CWPs) play critical roles in polysaccharides organization and remodeling processes during growth and upon environmental stresses [2,3]. Cell wall proteomics has revealed the great diversity of CWPs and allowed the discovery of unexpected CWP families [4]. The combination of genetics and biochemistry approaches has allowed demonstrating the roles of CWPs in polysaccharide metabolism, biosynthesis of lipid-rich cell wall layers, lignin monomer polymerization, but also in signaling and ROS homeostasis maintenance [5,6,7,8].

Among the newly described CWPs families, the importance of the PAC (Proline-rich Arabinogalactan protein and Conserved Cysteines) domain containing-protein (PDP) family could be stressed because of their presence in many cell wall proteomes (see *WallProtDB*, www.polebio.lrsv.ups-tlse.fr/WallProtDB/, query with “Ole e1 allergen domain” as a keyword). The name of the PDP family was initially proposed by Baldwin et al. [9], who described them as a sub-family of non-classical arabinogalactan proteins (AGPs) containing both an AGP domain and a C-terminal domain containing six Cysteines residues (named Cys 1 to Cys 6 herein). Later on, a domain partly describing the PAC domain has been proposed in the Pfam database (PF01190, http://pfam.xfam.org/). The firstly described member of this family was a protein from *Nicotiana alata* named AGPNa3 [10]. Then, several proteins very close to AGPNa3 were studied, for a review, see [11]. As examples, the following ones can be mentioned: *Daucus carota* DcAGP1 [12]; *Arabidopsis thaliana* AtAGP30 [13], and AtAGP31 (At1g28290) [14]; *Capsicum annuum* CaPRP1 [15]; *Gossypium hirsutum* GhAGP31 [16]; and *Petunia hybrida* PhPRP1 [17]. More recently, it appeared that the PAC domain could also be found alone, located at the N-terminus of the mature protein or associated with different types of domains, such as a Histidine-rich region, an *O*-glycosylated Proline/Hydroxyproline-rich domain, or an extensin domain [18,19].

Functional studies on several of the *A. thaliana* PDPs have shown their diverse roles during plant development. *PRPL1* (*Proline-Rich Protein-Like*, *At5g05500*) has a trichoblast-specific expression and plays roles in root hair elongation, as shown by the reduction in length of root hairs in the *prpl1* mutant [20]. Plants lacking *FOCL1* (*Fused Outer Cutiular Ledge 1*, *At2g16630*) produce stomata without a cuticular ledge, and thus, focl1 mutants display drought tolerance [21]. *AtAGP30* (*At2g33790*) is involved in root regeneration in vitro and in the timing of seed germination [13]. *AtAGP30* is expressed in root atrichoblasts under the control of ABA signaling [22]. *AtAGP31* is expressed in vascular tissues and repressed by methyl jasmonate at the transcriptional level [14]. AtAGP31 has also been shown to accumulate in actively growing etiolated hypocotyls [23]. In vitro interactions have been demonstrated between its PAC domain and galactans or the Gal-Ara-rich *O*-glycans of its Proline/Hydroxyproline rich domain [11]. These studies have led to the assumption that AtAGP31 could be involved in cell wall non-covalent protein/polysaccharide networks playing roles during quick cell elongation [11].

Recently, the crystal structure of the PAC domain of an allergenic protein from *Plantago lanceolata* containing an N-terminal PAC domain (Pla l 1 as a member of the Ole e 1–like protein family, PDP code 4Z8W) has been determined, highlighting the importance of β-sheets in its secondary structure [24]. In particular, the structure revealed a seven-stranded β -barrel with four loop regions. Three intramolecular disulfide bonds were found between (i) β 1b and β 6 strands (Cys 1-Cys 5), (ii) β 2 and β 5 strands (Cys 3-Cys 4), and the (iii) C-terminus and loop C-terminal of β 2 strand (Cys 2-Cys 6), thus forming a closed branched loop. A detailed characterization of allergens of the same protein family allowed proposing that they share the same core structure, whereas loop regions can be heterogeneous.

In this article, we aim at giving an evolutive overview of the PDPs throughout the green lineage, from Bryophytes to late divergent plants, such as monocots and dicots. We first define more precisely the PAC domain characteristics in order to retrieve PAC domain sequences from available genomic or RNA-seq databases using a tailor-made bioinformatic script. Since the conservation of the primary amino acid sequences of PAC domains was rather low, and since the presence of β -sheets seemed to be essential for domain folding, bona fide PAC domains were selected according to their secondary structure conservation, and protein alignment was done using a software taking into account secondary structures. Modeling of tertiary structures was done based on the available crystal structure of the Pla l 1 PAC domain. Finally, we could draw a phylogenic tree and sort the PAC domains according to their association with other domains. We could also investigate the occurrence of PAC domains in ancestor organisms.

## 2. Results and Discussion

### 2.1. Characteristics of the PAC Domain and Search for New PDP Candidates

**Figure 1:**
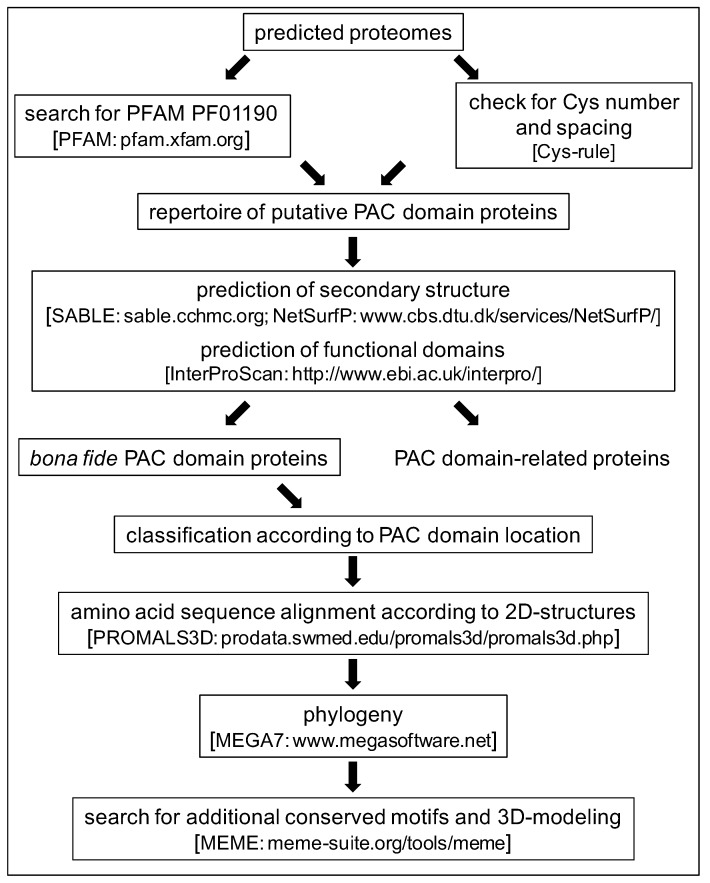
Pipeline for Proline-rich Arabinogalactan protein and Conserved Cys (PAC) domain protein identification and phylogeny. The name of the bioinformatics programs and resources used at each step are indicated in brackets.

The overall strategy used for this study is summarized in Figure 1. As a first step and in order to obtain a better definition of a PAC domain, orthologous sequences have been identified in the *A. thaliana* genome using that of the AtAGP31 PAC domain. Altogether, 14 candidate sequences were identified and manually checked for the presence of the six conserved Cys residues: At1g29140, At1g78040, At3g09925, At4g08685, At4g18596, At5g45880, At5g54855, AtAGP31, At5g05500 (PRPL1), At5g15790, At2g34790 (AtAGP30), At2g34700, At4g18596, and At2g16630 (FOCL1). These sequences were then used to identify additional PDPs by sequence similarity in eight other angiosperm genomes: *Amborella trichopoda*, *Brachypodium distachyon*, *Oryza sativa*, *Sorghum bicolor*, *Populus trichocarpa*, *Eucalyptus grandis*, *Linum usitatissimum*, and *Gossypium raimondii*. About 50 putative PDPs were collected and manually checked for the presence of the six conserved Cys residues. From this first data mining step, it appeared that the level of conservation of the amino acid sequences of the PAC domains could be low. In particular, except between the two first conserved Cys residues (Cys 1 and Cys 2), the spacing between Cys residues could be variable. Thus, the usual homology-based mining was not sufficient, and an alternative strategy was necessary to obtain exhaustive results for each plant. The alignment of angiosperms PAC domains has allowed calculating the range of spacing between the conserved Cys residues. Then, a tailor-made script based on several points detailed in Table 1 has been set up to search for additional PDPs in the same genomes or in other genomics or transcriptomics databases. However, the prediction of a signal peptide for protein secretion could not be made systematically for the proteins translated from transcriptomics data because the sequences could be incomplete. Furthermore, when genomic sequences were available, the presence of an intron between the sequences encoding, on the one hand, Cys 1 and Cys 2, and on the other hand, Cys 3 to Cys 6 was searched for to support the PAC domain identification.

Using this script, sequences encoding PAC domains have been searched for in 78 plant species belonging to the green lineage from Bryophytes (*Bryophyta*, *Marchantiophyta* and *Anthocerotophyta*) to late divergent plants. Altogether, about 450 putative PAC domain sequences were collected (S1–S4).

Three additional criteria have then been used to select bona fide PAC domain proteins. The first one was the number of conserved Cys residues. Indeed, we have found putative PAC domains showing the expected characteristics, but containing only five Cys residues, or containing more Cys residues, up to nine (S1,S5). Although some of them had sequences very similar to those of six Cys-containing PAC domains (S5), we have decided to dismiss them in case of a lack or an excess of Cys residues, which would modify the folding of the domain by generating disulfide bridges different from the expected ones. The second exclusion criterion was the absence of predicted β-sheets. Indeed, the crystal structure of the Pla I 1 PAC domain has allowed highlighting the importance of these β-sheets in its secondary structure [24]. Some proteins with large predicted α-helices and/or no predicted β-sheets have been dismissed with regard to this criterion, especially in Bryophytes, Equisetales, and Alismatales (S1,S6). The third criterion was the presence of associated predicted functional domains suggesting intracellular functions like aldehyde dehydrogenase domain (PF00171, *Tetraphis pellucida* HVBQ_2004216) or JmjC and JmjN domains of transcription factors (PF02373 and PF02375, *Pallavicinia lyelli* YFGP_2007785) (S3). In most of these latter cases, it was not possible to predict the sub-cellular localization of the proteins because they resulted from the translation of incomplete contigs obtained from RNA-seq data.

### 2.2. The Number and the Diversity of PAC Domain Proteins Increase Along the Green Lineage

**Figure 2:**
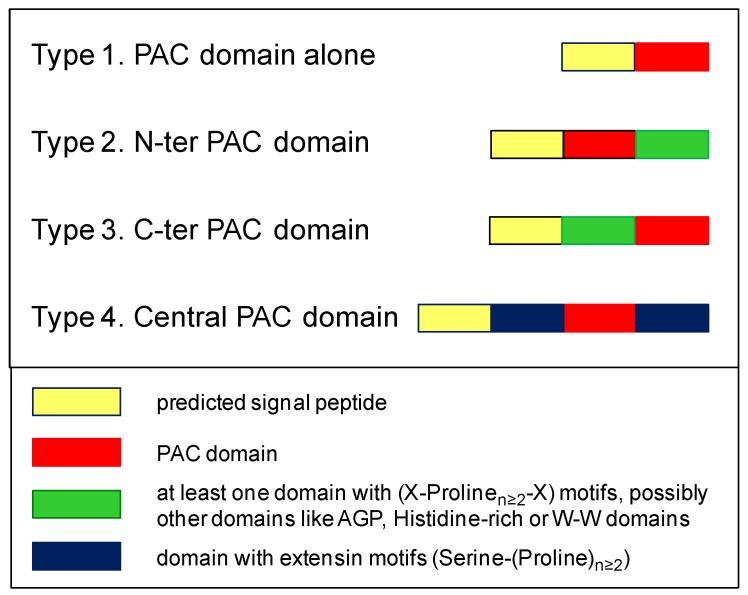
Classification of PAC domain proteins according to the location of the PAC domain and associated domains. **Type 1.** Proteins containing the PAC domain alone. **Type 2.** Proteins containing the PAC domain at their N-terminus together with another (several other) domain(s). **Type 3.** Proteins containing the PAC domain at their C-terminus together with another (several other) domain(s). **Type 4.** Proteins containing the PAC domain in a central position flanked by two domains with extensin motifs (Serine-(Proline)_n ≥ 2_).

The PDPs have been classified according to the domains associated with the PAC domain. Four types were distinguished (Figure 2). Type 1 corresponds to proteins only containing a PAC domain. The corresponding genes could exhibit either no intron or one intron between the sequences encoding Cys 1 and Cys 2 and those encoding Cys 3 to Cys 6. Type 2 includes proteins with an N-terminal PAC domain, which could be associated to (i) a Proline-rich domain or (ii) a well-conserved domain of unknown function usually encoded by a specific exon and starting with the following amino acid motif: Tryptophane-X8-Tryptophane (W-W domain) (S7). As an example, At2g16630 (FOCL1) is a type 2-PAC domain protein with a W-W domain at the C-terminus. Type 3 encompasses proteins with a C-terminal PAC domain. The PAC domain could be associated with a Histidine stretch, a Proline-rich domain, and/or an AGP domain. For example, AtAGP30 and AtAGP31 are type 3-PAC domain-proteins. Finally, type 4 corresponds to proteins containing central PAC domains flanked by two extensin domains. Although a few proteins with Serine-(Proline)_4_ motifs typical of extensins at their C-terminus were found in *Anthocerophyta* and Lycopodiales, the first bona fide type 4-PDP was found in Psilotales. There is no such PDP in *A. thaliana*.

**Figure 3:**
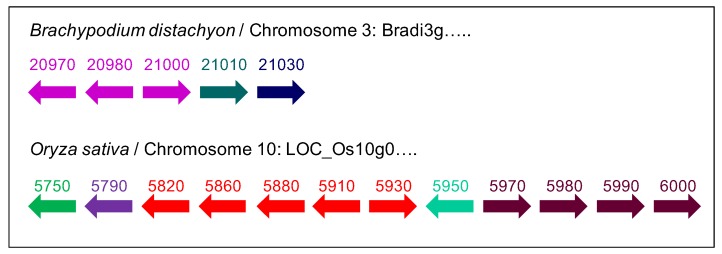
Examples of domain containing-protein (PDP) genes organized in tandem in the *B. distachyon* and *O. sativa* genomes. The orientation of the genes is indicated by arrows. The names of the genes are abbreviated, e.g., 20970 stands for *Bradi3g20970*, and 5750 for *O. sativa LOC_Os10g5750*. Genes sharing more than 85% identity in their PAC domain coding sequences at the amino acid level are represented with arrows of the same color.

In Bryophytes and Anthocerotophyta, only one to three PAC domain proteins were found for each species (S1). The number of PDPs was higher in Psilotales and Equisetales as well as in all the plant families, which have appeared later in the green lineage. Eleven PDPs are present in *Amborella trichopoda,* which is considered as an ancestor common to angiosperms [25]. The highest numbers of PDPs, i.e., between 17 and 23, were found in Poales, *Brachypodium distachyon*, *Sorghum bicolor*, *Zea mays,* and *Oryza sativa*, as well as in *Linum usitatissimum*, *Populus trichocarpa,* and *Gossypium raimondii*. In Poales like *B. distachyon* and *O. sativa*, the genes encoding PDPs could be found in tandem (Figure 3). The PAC domains of these genes could show a high degree of identity (more than 85%), supporting the recent tandem duplication events [26]. In addition, PAC domains with various numbers of Cys residues were also found in Poales (S1). The functionality of those PAC domains has not yet been established.

**Figure 4:**
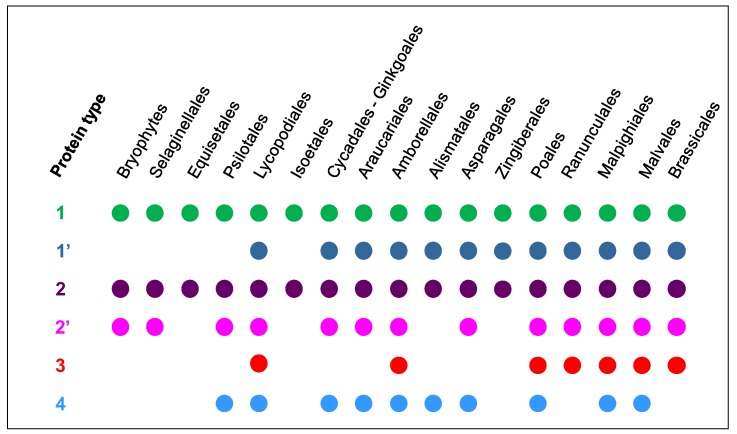
Distribution of the different types of PAC domains within the plant families. The different types of PAC domains are represented in Figure 2. Among type 1-PAC domains, those having a highly conserved amino acid sequence are distinguished (1’). Among type 2-PAC domains, those that are associated to a C-terminal W-W domain are highlighted (2’).

The different types of PDPs are unevenly distributed within the different plant species (Figure 4). Only type 1- and type 2-PDPs were found in all plant families. Among the type 1-PDPs, one sub-type should be distinguished. It corresponds to highly conserved sequences throughout the green lineage since Lycopodiales with an overall percentage of identity ranging from 60% to 88% and a percentage of similarity from 69% to 92%. For comparison, the percentage of identity and of similarity between two PAC domain sequences can be rather low (15.4% and 20.7%, respectively). Among the type 2-PDPs, those including a C-terminal W-W domain are present in nearly all plant families from Bryophytes to Brassicales. They could appear as ancestors of PDPs. Type 3- and type 4-PDPs seem to have appeared more recently in the evolution of the green lineage since the most ancient type 3- and type 4-proteins were found in *A. trichopoda* and in Psilotales, respectively. Of course, one cannot exclude that some PDPs are missing in this collection since only a few complete genomes are available for plants from Psilotales to Amborellales.

### 2.3. A Possible Origin for the PAC Domain

We have performed an extensive search of PAC domain sequences in the available databases dedicated to ancestors of the green lineage using both the script described above and BLAST queries using several PAC domains in case the spacing between Cys residues would be slightly different. Mining was done in the following families: Stramenopiles (*Synura petersenii*), Cryptophyta (*Chroomonas sp*), Chlorophyta (*Asteromonas gracilis*, *Chlamydomonas rheinardtii*, *Nephroselmis olivacea*, *Volvox carteri*, *Scenedesmus dimorphus*, *Scherffelia dubia*), Streptophyta (*Chara braunii*, *Coleochaete orbicularis*, *Klebsormidium flaccidum*, *Mesotaenium caldariorum*, *Penium margaritaceum*) (S4). In many cases, the proteins were incomplete either at their N-termini and it was not possible to predict a signal peptide, or at their C-termini, and they could not be classified. Whenever possible, the presence of predicted functional domains associated to the putative PAC domains was checked, and the proteins comprising functional domains associated to intracellular functions were not retained. 

We could only find PAC domain-related sequences in *Chlorophyta*: 10 proteins were found in *C. rheinardt*ii and one in *V. carteri* ,which both belong to Chlamydomonales. The Glycine residue located upstream the first Cys residue was always missing, and the PAC domains were associated with Proline-rich motifs of two types: either Serine-(Proline)_n_ or (Proline)_n_ and up to three of them could be found in a given protein. However, the secondary structures of these domains were predicted to be α-helices. In *C. rheinardtii*, the GP1 and GP2 proteins, which both have Serine-(Proline)_n_ motifs, were described as proteins rich in Hydroxyproline resides forming the insoluble glycoprotein framework of the cell wall [27,28]. Furthermore, in *C. orbicularis*, we could find another interesting PAC domain candidate, which was associated to Proline-rich motifs but contained seven Cys residues. The highest level of identity/similarity was found with two PAC domains of *Musa acuminata*: GSMUA_Achr4T17330 (45%/51%) and GSMUA_Achr7T01790.1 (39%/50%). The highest level of identity/similarity with a *Marchantiophyta* PAC domain was found with the *Conocephalum conicum* PAC domain ILBQ_2004952 (30%/46%) and the *M. polymorpha* Mapoly0014s0128 PAC domain (33%/45%). Altogether, the sequence showing the highest level of identity to bona fide PAC domains was found in *C. orbicularis*. This is consistent with the assumption that the Coleochaetales could be one of the ancestors of the green lineage [29].

### 2.4. Three-Dimensional-Modeling of PAC Domain Proteins

Three-dimensional-models were calculated for 41 bona fide and 9 putative PAC domains, based on the crystal structure of the *P. lanceolata* PAC domain [24]. The sequence identities between the template and the PAC domains varied between 9.6% and 30.4% (median 15.9%). A sequence identity of 30% is generally seen as a lower limit for reliable models predicted by homology modeling algorithms, but the assumption of disulfide bridges somewhat lowers this limit. However, the low sequence similarities were still an issue. In addition, in 6 out of the 50 PAC domains, the 3D-modeling software I-Tasser was not able to find conformations enabling the formation of the three disulfide bridges between the predefined Cys residues (S8). In all these cases, either the proteins were predicted to have α-helices, or they were missing the Glycine residue upstream Cys 1.

**Figure 5:**
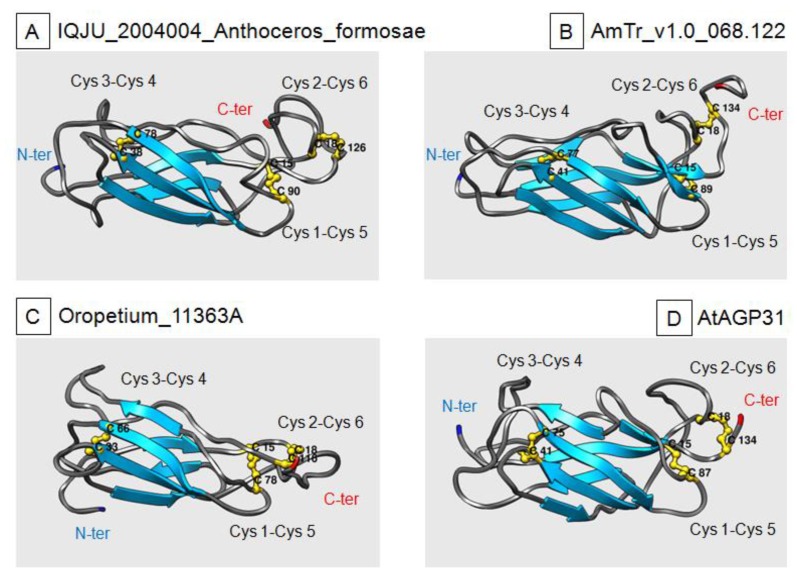
3D-modeling of four PAC domains. (**A**) A representative PAC domain of Bryophytes: IQJU_2004004_Anthoceros_formosae. (**B**) A PAC domain of *A. trichopoda*: AmTr_v1.0_068.122. (**C)** A representative PAC domain of the *O. thomaeum* monocot: Oropetium_11363A. (**D)** A representative PAC domain of the *A. thaliana* dicot: At1g28290. The N-terminus (N-ter) and the C-terminus (C-ter) of the proteins are indicated in blue and red, respectively. Blue ribbons represent β-sheets. The three disulfide bridges are drawn in yellow, and the names of the Cys residues involved are indicated.

For the bona fide PAC domains, it was possible to propose relevant 3D-models fitting with the typical structure experimentally demonstrated for the *P. lanceolata* PAC domain [24]. Four selected PAC domains from different plants are shown in Figure 5: an *Anthocerophyta* (*Anthoceros formosa*), chosen as an ancestral plant, *A. trichopoda* as the common ancestor to flowering plants, and two higher plants, *Oropetium thomaeum* and *A. thaliana*. All four 3-D models show the expected parallel β-sheets forming a β-barrel and the three disulfide bridges. They also contain loop regions as the *P. lanceolata* PAC domains. The 3D-structure of bona fide PAC domains seems to have been conserved through the evolution of the green lineage. However, the *C. orbicularis* protein, which was assumed to be an ancestor of the PDPs in the green lineage, only had three β-sheets, but the three disulfide bridges were at the predefined positions (S8).

The PAC domains that have been considered apart because of the prediction of α-helices showed completely different 3D-structures (S8). They exhibited less β-sheets or only α-helices, and as mentioned above, the three disulfide bridges were not at the expected positions. The 3-D modeling, thus, brought an additional criterion to confirm bona fide PAC domains. Interestingly, such a β-barrel structure has already been described for a mannose-binding lectin family of red algae, the *Oscillatoria Agardhii* Agglutinin-Homolog (OAAH) mannose-binding lectin family [30]. In this case, two β-barrels associate perpendicularly to build up the complete 3D-structure of the molecule, and the interaction with cell wall polymers occurs at two crevices symmetrically located at its two ends [31]. This role would be consistent with the finding that the PAC domain of AtAGP31 can interact with cell wall polysaccharides and *O*-glycans in vitro [11].

To test the role of the conserved Cys residues and, therefore, that of disulfide bridges in 3D-structure stability, in silico mutation experiments have been performed. Possible 5 Cys-PAC domain variants have been tested for the *P. lanceolata* PAC domain, and for each of the eight A. trichopoda PAC domains, which were considered as representative of the eight phylogenic clades (see below). Each Cys residue has been replaced by a Ser residue, and the change in stability was determined by MAESTRO (S11). In all cases, positive values of the ddG parameter indicating changes in unfolding free energy were found, indicating destabilization of the 3D-structure. Altogether, it seems that the conserved Cys residues are critical for the stability of the β-barrel. This could indicate that the domains lacking one Cys residue could be impaired in their biological activity or more sensitive to changes in their physiological environment. The presence of a seventh or even an eighth Cys residue could have different consequences depending on the position(s) of the additional Cys residue(s). Such residue(s) could be involved in different disulfide bridges or not. Only experimental work could allow showing any change in the biological activity of the PAC domain.

### 2.5. Phylogenetic Analyses Reveal the Presence of a Few Clades Grouping the PAC Domain Proteins According to their Associated Domains

Based on all the criteria described above, 300 PAC domains have been selected for the building of phylogenetic trees (S1,S2). They have been chosen from plant families representative of the green lineage from Bryophytes to Brassicales based on a phylogenetic tree established using plastid gene sequences [32]. When several species were available for a given plant family, only one or a few of them were selected to represent it. For each plant family, the PAC domains sequences were analyzed for their percentage of identity, and the most representative plant species was retained. When the sets of PAC domain sequences were too different between plants of the same family, several species could be maintained. In addition, only PAC domains showing less than 85% of identity inside a given plant species were conserved. As a first step, the sequences were aligned according to their predicted secondary structure. Such a strategy was used in previous studies where the conservation of the primary sequences of the proteins was not sufficient to ensure relevant alignments [33,34,35]. The PROMALS3D software was used, and the resulting alignment was introduced in the MEGA7 software to build up a maximum likelihood tree using 500 bootstraps. Due to the low level of conservation between amino acid sequences and especially between the PAC domain sequences of the older lineages, we have decided to build up two independent trees to avoid bias due to long-branch attraction: the first one (Tree I) including plants from Bryophytes to *A. trichopoda*, and the second one (Tree II) from *A. trichopoda* to Brassicales.

Regarding Tree I, it is difficult to define clades grouping all the PAC domain sequences because most of the bootstrap values were low (S9). We only considered clades corresponding to bootstrap values higher than 30. We could define seven clades grouping 71% of the retrieved PAC domains, six of them containing one *A. trichopoda* PAC domain: clade A (AmTr.v1.0.061.7, mostly type 1-PAC domains); clade B (AmTr.v1.0.066.9, type 4-PAC domains); clade C (AmTr.v1.0.062.88, type 1-PAC domains, highly conserved sequences); clade D (AmTr.v1.0.041.161, type 2 W-W domains); clade E (AmTr.v1.000047, type 2-PAC domains); clade J (AmTr.v1.0.041.169, type 2-PAC domains); and clade K (*Equisetum sp* PAC domains). The distribution of the PAC domain sequences of the other species was not clear. PAC domains of Bryophytes were represented in clades A, D, and E, whereas a *Tmesipteris parva* (Psilotale) PAC domain was found in clade B, and a *Phylloglossum drummondii* (Lycopodiale) PAC domain in clades C, and J. Of course, one cannot exclude that PAC domains of plants, which have divergent earlier than Amborellales are still missing since only a limited number of fully sequenced genomes are available. Despite the presence of the key Cys residues and of conserved 3D-structure, the large evolutive distance existing between Bryophytes and *A. trichopoda* together with a relaxed selective pressure could explain the low sequence identity observed between sequences of Tree I. Indeed, whereas terrestrialization is assumed to have occurred 450 MYA [36], the age of angiosperms emergence was estimated to be between 169-199 MYA [37]. Based on the putative interaction with cell wall polysaccharides and *O*-glycans, the PAC domain sequence variability could be correlated with the variability of the cell wall composition from Bryophytes to angiosperms [38].

**Figure 6:**
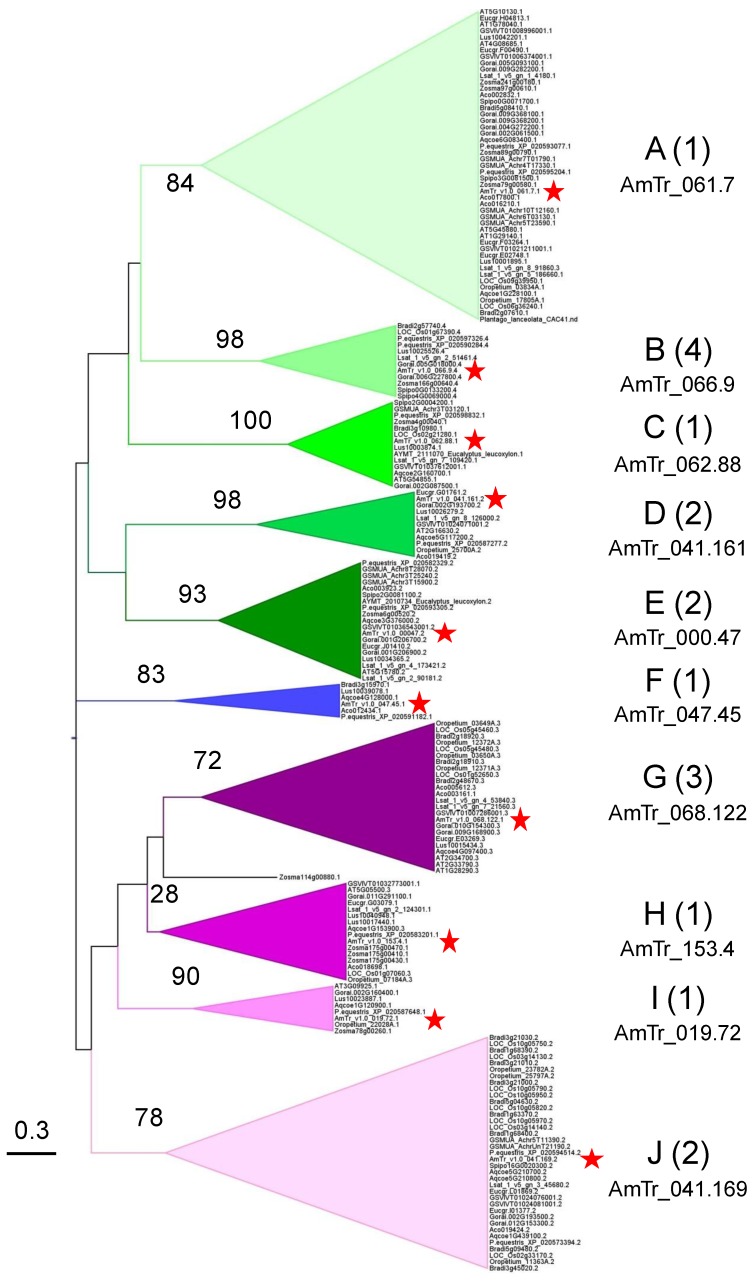
Phylogenetic Tree II. Tree II was built up using 196 PAC domains sequences from *A. trichopoda* to *A. thaliana*. Ten clades (A to I) were defined according to significant bootstrap values (higher than 72, with the exception of clade B). The type of PDPs (e.g., Type 1 is 1, see Figure 2) found in each clade indicated between brackets. The name of the *A. trichopoda* PDP found in each clade is indicated and highlighted with a red star.

In Tree II, the PAC domains were distributed into 10 clades with high confidence bootstrap values (from 72 to 100) with the exception of clade H (28) (Figure 6, S9). An *A. trichopoda* PAC domain was found in each of them. Four clades were specific to higher plants, each of them, respectively, comprised the following *A. trichopoda* PAC domains: AmTr.v1.0.047.45 (clade F); AmTr.v1.0.068.122 (clade G); AmTr.v1.0.153.4 (clade H); and AmTr.v1.0.019.72 (clade I). Monocot and dicots were represented in all the clades, but clade J comprised a high number of grass PAC domains originating from gene duplication (see above). Interestingly, although the tree has been built up with PAC domains only, they grouped according to their association to other domains: type 1-PAC domains were found in clades A, C, F, H, and I; type 2-PAC domains were grouped in clades D, E, and J, with type 2 W-W domains in clade D; type 3-PAC domains were found in clade G with the exception of three of them in clade H with short Proline-rich motifs at their N-terminus; and type 4-PAC domains were only found in clade D. Thus, it seems that there is a link between the amino acid composition of PAC domains, their secondary structure, and the associated domains. Finally, it seems that all the PAC domains of higher plants have a counterpart in *A. trichopoda*, meaning that the modern multi-domain structures of the PDPs found in the ten angiosperm clades preceded the emergence of angiosperms.

### 2.6. Conserved Amino Acids Motifs Inside Clades

**Figure 7:**
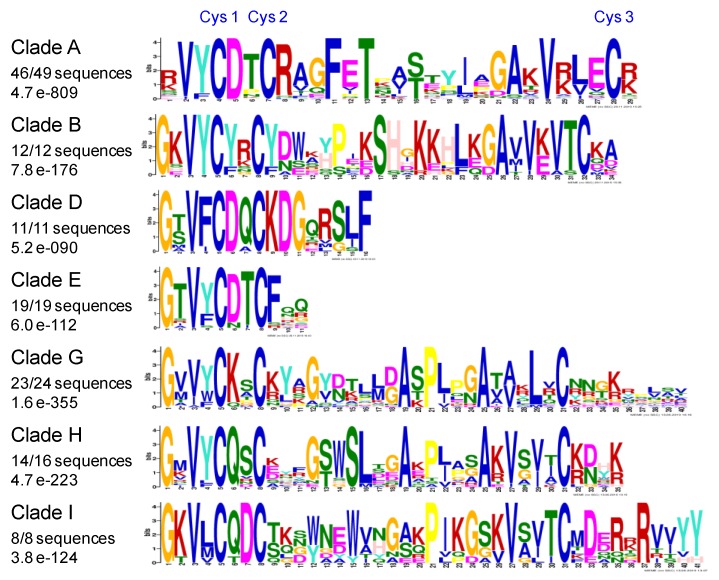
The most conserved motifs of PAC domains inside clades A, B, D, E, G, H, I in PDPs of *A. trichopoda* plant families appeared subsequently. The number of PAC domains in each clade is indicated as well as the score of the conserved motif according to the MEME software.

**Figure 8:**
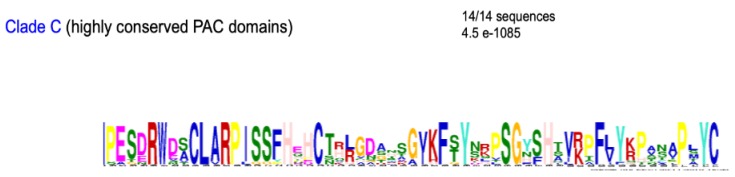
The most conserved PAC domains from clade C. The comparisons have been made between 18 PAC domain sequences from Lycopodiales to Brassicales.

A search for conserved amino acid motifs was done for the PAC domains of each clade of Tree II. The most significant results were found for clades A, B, D, E, G, H, and I (Figure 7). In each clade, the most conserved motifs were detected at the N-terminus of the PAC domain. This was consistent with the definition of the pollen Ole e 1 motif in the Pfam and Prosite databases (PF01190 and PS00925, respectively). However, the consensus defined for the PS00925 domain only exactly fitted with that of clade A PAC domains ([EQT]-G-x-V-Y-C-D-[TNP]-C-R). Furthermore, the most conserved PAC domains were found in the C clade (Figure 8). Their degree of conservation in the green lineage from Lycopodiales to Brassicales is impressive. Finally, the C-terminal W-W domain present in all the proteins belonging to clade D was also very well conserved from the Bryophytes to the Brassicales with common motifs mostly located in its N-terminus half (S10).

The combination of sequence conservation with the accessibility of conserved residues on the protein surface shall hint to functional important sites while conserved residues located in the protein core are more likely important for maintaining the fold. Also, conserved residues in the loop regions may have a functional role, although they are less accessible in the static 3D-structural model as loops are often flexible and may move considerably. We, therefore, defined a representative 3D-model for each clade and obtained the solvent accessibility and secondary structure for each residue and aligned this information with the sequence profiles (S12). Indeed, many of the conserved sites are inaccessible to the solvent and located within or close to the β-sheets and, thus, are expected to maintain the fold. Candidates for the functional role are, for example, in clade A a Phe-x-Thr pattern (profile position 11–13); in clade B, a cluster of basic residues at position 18-22; in clade D, the conserved charged residues Lys and Asp at position 9 and 10; or in clade H, the amino acids Lys and Arg at position 35. The reliability of such assumptions depends on the quality of the structural models. We calculated a model quality score with MAESTRO and related the scores of the models to scores of experimentally determined structures (S13). The scores of the models are in the range of the modeling template structure (PDB code 4Z8W), indicating that none of the models should be largely wrong.

The conservation of motifs in PAC domains suggests common biological activities. It is possible to infer that their interactions with cell wall polysaccharides or *O*-glycans assumed from in vitro studies have been conserved and that the distribution of PDPs in the different plant families reflects differences in cell wall polysaccharides. Regarding the W-W C-terminal domain of the clade D PAC domains, its role remains to be unraveled. It is encoded by a distinct exon and could originate from exon shuffling [39].

## 3. Materials and Methods

### 3.1. Databases

The sequences used in this study have been retrieved from different databases, such as Orchidstra 2.0 ([40] http://orchidstra2.abrc.sinica.edu.tw/orchidstra2/orchid_blast.php), genome annotation Databases ([41], http://genome.microbedb.jp/blast/blast_search/klebsormidium/genes), Phytozome ([42], https://phytozome.jgi.doe.gov/pz/portal.html), OneKP ([43], https://db.cngb.org/onekp/) (see S1). When necessary, nucleotide sequences have been translated into amino acid sequences using EMBOSS transeq ([44], https://www.ebi.ac.uk/Tools/st/emboss_transeq/).

### 3.2. Comparisons and Alignment of PAC Domains

The BLAST (https://blast.ncbi.nlm.nih.gov/Blast.cgi) program has been used for sequence comparison. Similarities between PAC domain sequences have been calculated using either Blast2seq (https://blast.ncbi.nlm.nih.gov/Blast.cgi) or needle (http://www.bioinformatics.nl/cgi-bin/emboss/needle). The sub-cellular localization of proteins has been predicted with TargetP-2.0 ([45], http://www.cbs.dtu.dk/services/TargetP/) and the presence of β-sheets and/or α-helices using SABLE ([46], http://sable.cchmc.org/) and NetSurfP ([47], http://www.cbs.dtu.dk/services/NetSurfP/). The selected PAC domains starting at the Gly amino acid located three amino acids upstream of Cys 1 and ending at Cys 6 have been aligned using PROMALS3D ([48], http://prodata.swmed.edu/promals3d/promals3d.php) to take into account the prediction of α-sheets. The phylogeny has been calculated using MEGA7 ([49], https://www.megasoftware.net/) with the maximum likelihood option and 500 bootstraps. The presence of the PROSITE (PS00925, [50], https://prosite.expasy.org/) and Pfam (PF01190, [51], http://pfam.xfam.org/) domains have been checked in the retrieved sequences. Inside clades, conserved motifs have been identified using MEME ([52], http://meme-suite.org/tools/meme) or WebLogo3 ([53], http://weblogo.threeplusone.com/).

### 3.3. Three-Dimensional Modeling

For a subset of PAC domains, models were generated utilizing MODELLER [54] and I-Tasser [55]. Thereby, disulfide bridges were defined beforehand based on alignments with PDB entry 4Z8W corresponding to the *P. lanceolata* PAC domain [24]. Subsequently, these models were scored with MAESTRO [56], DOPE [57], and ProSA 2003 [58]. Then the top-scoring models were relaxed with Rosetta [59], and finally, the relaxed models were scored with the same three methods.

We consistently used PAC domains from *A. trichopoda* as representative models for each clade. The relative solvent accessibility of these models was calculated by an adaptation of the Geometry library algorithm [60]. The secondary structure assignment was obtained by DSSP [61,62].

Both MODELLER and I-Tasser depend on template structures. MODELLER is a homology-modeling tool, which assumes significant sequence similarity between target and template structures in order to create a reliable alignment between them. Loops and sidechains are modeled with respect to the target sequence. The overall fold, however, is largely determined by the template structure. I-Tasser is a fold-recognition approach, where sequence similarity between target and template does not play a major role. Moreover, I-Tasser uses structural fragments rather than complete protein (domain) folds, from which the overall fold is built. The final model is not determined by a single template. As such, it should be better applicable for PAC domain sequences with low similarity to the Pla I 1 PAC domain.

## 4. Conclusions

This study has allowed better defining PDPs by combining amino acid sequences features, secondary structures, and 3D-modeling. This protein family has appeared early during the evolution of the green lineage. It has, however, not been possible to identify with certainty a PAC domain ancestor in the presumed precursor organisms of the green lineage even if the *C. orbicularis* PAC domain appeared as a possible candidate. The association of the PAC domain with Pro-rich sequences seemed to be an ancient event, the most ancient sequence carrying both a PAC domain and a Proline-rich domain being found in Bryophytes, and those carrying both a PAC domain and extensin domains in Psilotales. Despite a great amino acid variability between PAC domains, the tertiary β-barrel structure strengthened by three disulfide bridges has been conserved in all bona fide PAC domains. Finally, the subset of PAC domains belonging to Clade C is intriguing. Their very high level of conservation at the amino acid sequence level suggests that they play critical roles in plant cell walls. Defining the specificity of interaction of the different PAC domains with other cell wall polymers will be one of the next challenges to fully unravel the roles of PDPs in the cell wall architecture.

## Figures and Tables

**Table 1:** Five features of a bona fide PAC domain.

1. Present in a protein with a predicted signal peptide
2. Presence of six Cys residues downstream a Glycine residue and with a defined spacing ^1^: **Gly** (3) **Cys 1** (2) **Cys 2** (10,30) **Cys 3** (20,50) **Cys 4** (8,20) **Cys 5** (25,60) **Cys 6**
3. Prediction of β-sheets according to the crystal structure of the *Plantago lanceolata* (www.rcsb.org/structure/4Z8W) PAC domain protein
4. Possibly associated to AGP, extensin, X(Proline_n≥2_) X-rich, Histidine-rich, or W-W domains
5. No prediction of additional functional domains

**^1^** The number of amino acids between two successive Cys residues is indicated between brackets.

## Supplementary Materials

Supplementary materials can be found at https://www.mdpi.com/1422-0067/21/7/2488/s1 S1 Number of PAC domain and PAC domain-related proteins in different plants from Bryophytes to Brassicales; S2 Amino acid sequences of PAC domain proteins in the green lineage; S3 Some examples of PAC domain-related proteins containing predicted functional domains suggesting intracellular functions; S4 Amino acid sequences of PAC domain-related proteins in ancestors to the green lineage; S5 Amino acid sequences of putative PAC domains with only five Cys residues, more than six Cys residues, or no Gly residue upstream Cys 1; S6 Amino acid sequences of putative PAC domains with six Cys residues, but predicted α-helices; S7 Amino acid sequences of the PAC and W-W domains of Type 2-PDPs including a C-terminal W-W domain; S8 Top-scoring 3D-models of PAC domains and the corresponding scores. Some PAC domain 3D-models; S9 Expanded phylogenetic trees of PAC domains from Bryophytes to *A. trichopoda* (Tree I) and from *A. trichopoda* to Brassicales (Tree II); S10 The conserved W-W domain from PAC domains belonging to clade D from Bryophytes to Brassicales PDPs; S11 *In silico* mutagenesis experiment to test the stability of the 3D-structure of a set of PAC domains mutagenized on one of the six conserved Cys residues; S12 Solvent accessibility and secondary structure for each residue and alignment of this information with the conserved sequence profiles of PAC domains; S13 MAESTRO scores for PAC domain models in relation to MAESTRO scores for experimentally-determined structures taken from the PDB database.

## Abbreviations

PAC	Proline-rich, Arabinogalactan protein, conserved Cysteines
PDP	PAC Domain Protein
