# Supplementary material for: The Cell Wall PAC (Proline-Rich, Arabinogalactan Proteins, Conserved Cysteines) Domain-Proteins Are Conserved in the Green Lineage
Source: Int J Mol Sci. 2020 Apr 3;21(7):2488. doi: 10.3390/ijms21072488 (PMC7177597; doi:10.3390/ijms21072488)
Supplement: Supplementary file 1 [file ijms-21-02488-s001.zip › Supplementary files_R1_Nguyen-Kim, San Clemente et al/Nguyen-Kim, San Clemente et al._Supplementary file 11.pdf]

Supplementary file 11. *In silico* mutagenesis experiment to test the stability of the 3D-structure of a set of PAC domains mutagenized on one of the six conserved Cys residues.

The sequences of the PAC domains are given in Supplementary file 2.

In silico mutation experiment for possible five Cys-PAC domain variants. The six Cys residues have be replaced by Ser and the change in stability was determined by MAESTRO.

ddG ... change in unfolding free energy in kcal/mol, positive values indicate destabilization. Confidence ... values closer to 1 indicate better prediction confidence

Xray/Model	Mutation to Ser at	ddG	confidence
4z8w (template)	Cys 1	3.57549	0.77027
	Cys 2	2.12721	0.86736
	Cys 3	2.45255	0.8131
	Cys 4	2.75184	0.82611
	Cys 5	2.45704	0.82707
	Cys 6	1.32267	0.83781
AmTr_v1.0_061.7.1 (clade A)	Cys 1	3.39842	0.78146
	Cys 2	2.68815	0.83047
	Cys 3	2.98417	0.79527
	Cys 4	3.44757	0.78292
	Cys 5	2.76256	0.79688
	Cys 6	2.60587	0.81457
AmTr_v1.0_066.9.4 (clade B)	Cys 1	3.87618	0.76273
	Cys 2	2.55631	0.8366
	Cys 3	3.06021	0.80979
	Cys 4	3.81503	0.79575
	Cys 5	2.68014	0.82918
	Cys 6	2.14382	0.85645
AmTr_v1.0_062.88.1 (clade C)	Cys 1	3.42963	0.8024
	Cys 2	2.2582	0.88253
	Cys 3	3.03229	0.79154
	Cys 4	3.99712	0.76382
	Cys 5	2.79181	0.82795
	Cys 6	0.77256	0.91274
AmTr_v1.0_041.161.2 (clade D)	Cys 1	2.56958	0.84224
	Cys 2	1.6971	0.88547
	Cys 3	3.90044	0.76834
	Cys 4	3.90877	0.77109
	Cys 5	1.2632	0.89708
	Cys 6	2.3301	0.82932
AmTr_v1.0_00047.2 (clade E)	Cys 1	2.90614	0.80333
	Cys 2	2.78046	0.80662
	Cys 3	4.21589	0.73165
	Cys 4	4.33569	0.71088
	Cys 5	2.80004	0.80999
	Cys 6	1.45091	0.90019
AmTr_v1.0_068.122.1 (clade G)	Cys 1	3.65844	0.76977
	Cys 2	2.69138	0.81918
	Cys 3	2.82805	0.82595
	Cys 4	3.36102	0.81705
	Cys 5	2.72987	0.83278
	Cys 6	1.35604	0.89068
AmTr_v1.0_153.4.1 (clade H)	Cys 1	3.44759	0.80186
	Cys 2	1.70675	0.88404
	Cys 3	3.41888	0.78206
	Cys 4	3.40637	0.75515
	Cys 5	2.37172	0.8465
	Cys 6	1.24852	0.89123
AmTr_v1.0_019.72.1 (clade I)	Cys 1	3.30608	0.78534
	Cys 2	1.52879	0.87337
	Cys 3	3.37773	0.78849
	Cys 4	3.4131	0.78992
	Cys 5	2.40839	0.84372
	Cys 6	0.88844	0.92186
