# Supplementary material for: The Cell Wall PAC (Proline-Rich, Arabinogalactan Proteins, Conserved Cysteines) Domain-Proteins Are Conserved in the Green Lineage
Source: Int J Mol Sci. 2020 Apr 3;21(7):2488. doi: 10.3390/ijms21072488 (PMC7177597; doi:10.3390/ijms21072488)

**Supplementary file 12.** Solvent accessibility and secondary structure for each residue and alignment of this information with the conserved sequence profiles of PAC domains for each clade.

The conserved motifs are described in Figures 7 and 8.

Clade A

46/49 sequences
4.7 e-809

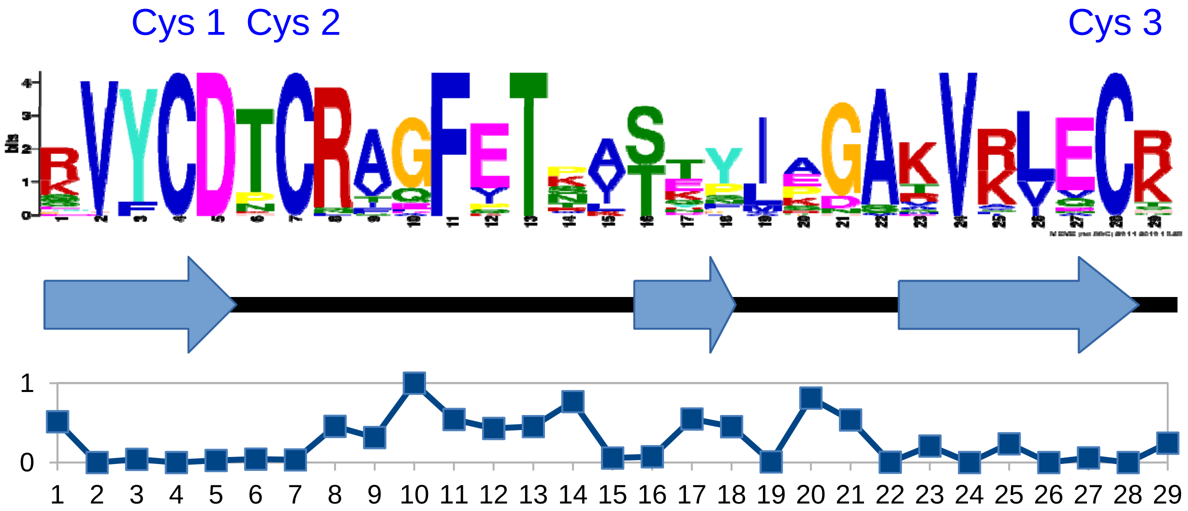


Clade B

12/12 sequences
7.8 e-176

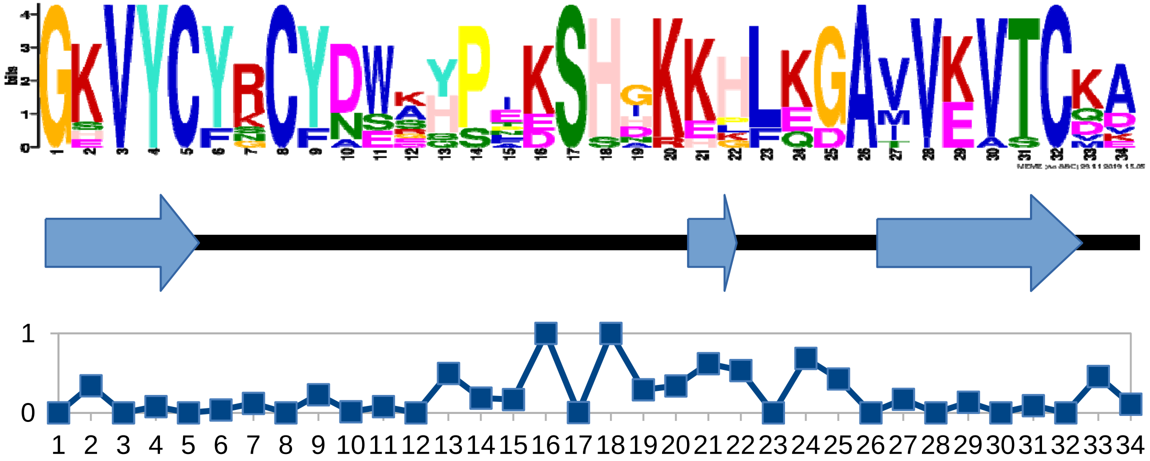

Clade C (highly conserved PAC domains)

14/14 sequences
4.5 e-1085

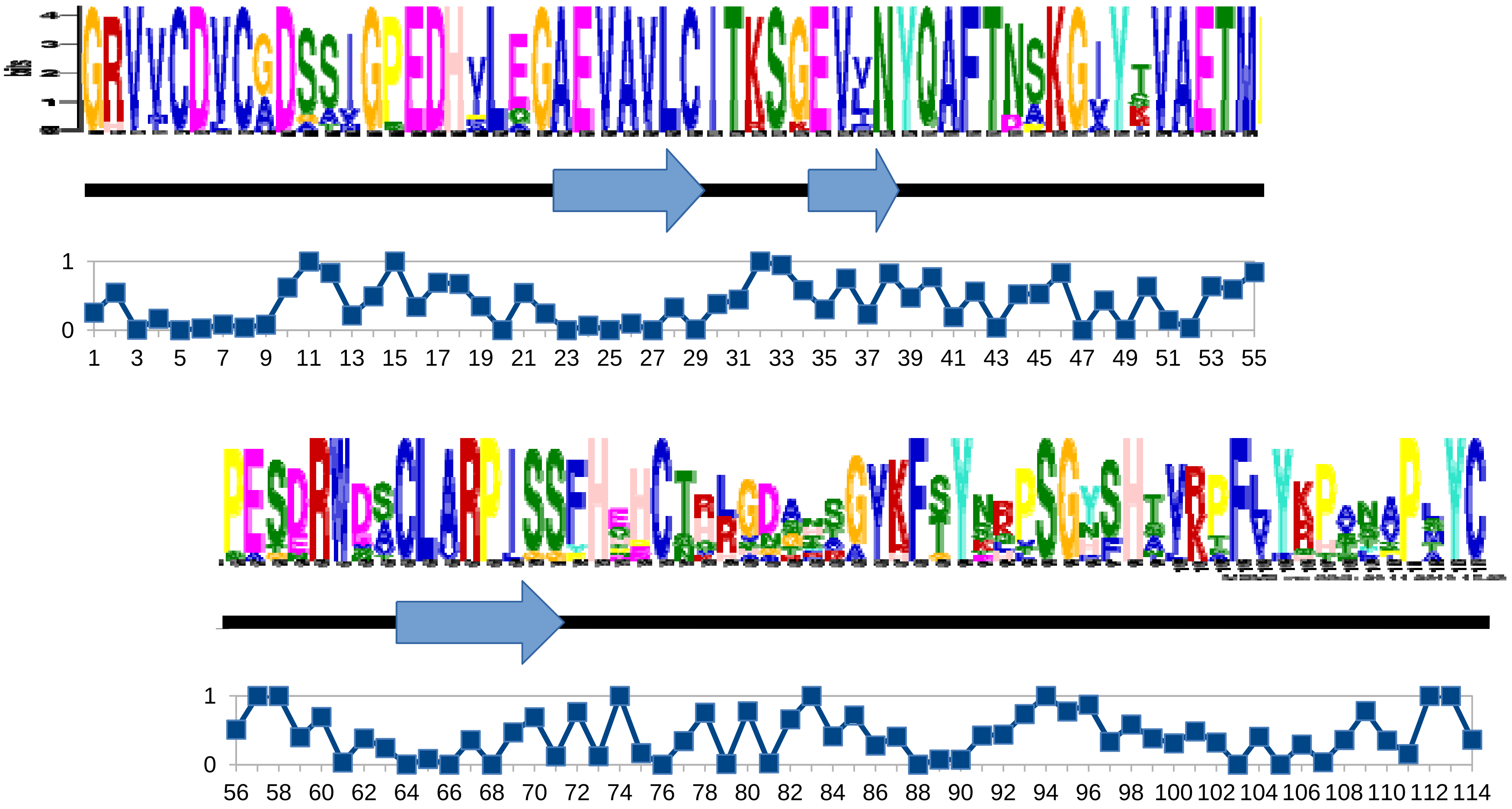

## Clade D

11/11 sequences
5.2 e-090

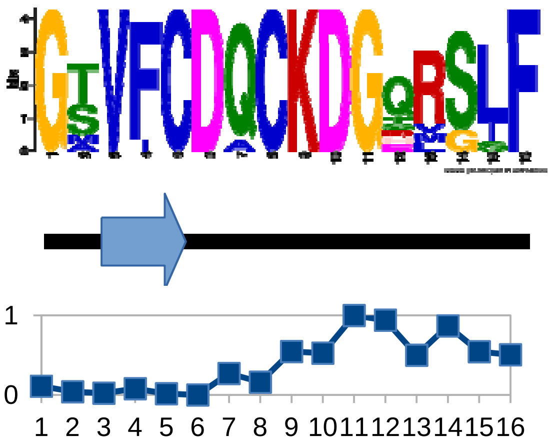


## Clade E

19/19 sequences
6.0 e-112

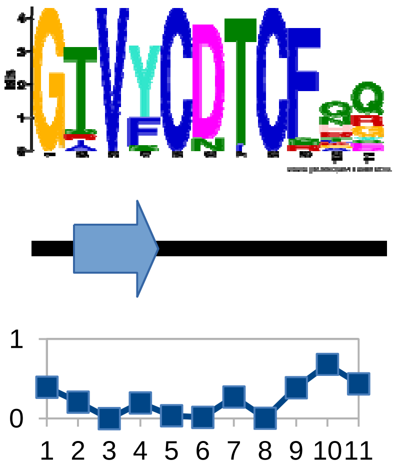

Clade G

23/24 sequences

1.6 e-355

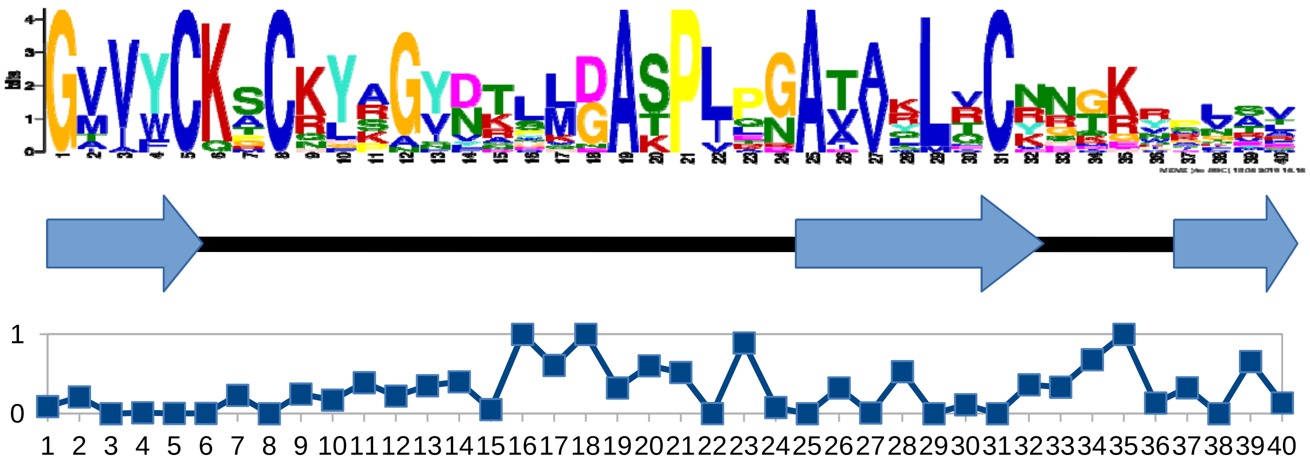

Clade H

14/16 sequences

4.7 e-223

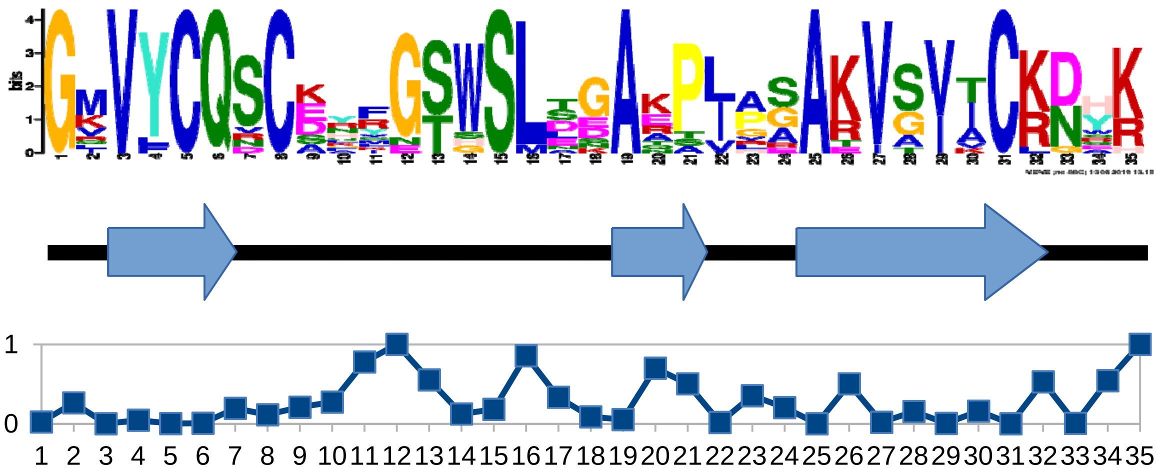

Clade I

8/8 sequences

3.8 e-124

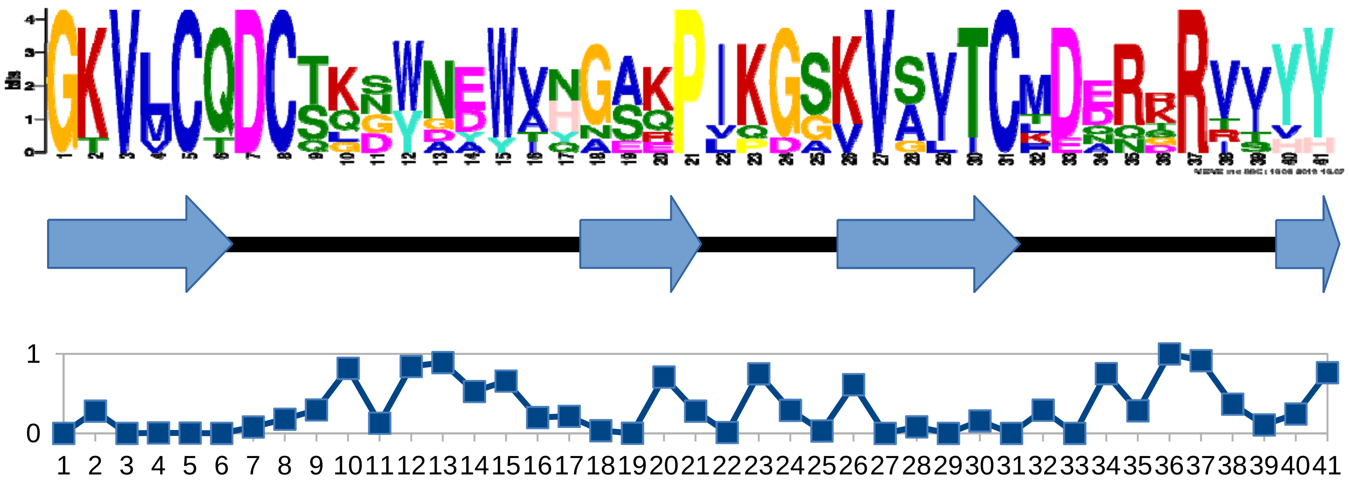

Supplement: Supplementary file 1 [file ijms-21-02488-s001.zip › Supplementary files_R1_Nguyen-Kim, San Clemente et al/Nguyen-Kim, San Clemente et al._Supplementary file 12.pdf]
