# Supplementary material for: The Cell Wall PAC (Proline-Rich, Arabinogalactan Proteins, Conserved Cysteines) Domain-Proteins Are Conserved in the Green Lineage
Source: Int J Mol Sci. 2020 Apr 3;21(7):2488. doi: 10.3390/ijms21072488 (PMC7177597; doi:10.3390/ijms21072488)

Supplementary file 13. MAESTRO scores for PAC domain models (large dark blue diamonds) in relation to MAESTRO scores for experimentally-determined structures taken from the PDB database (small diamonds). Only single-chain PDB entries with a chain length up to 500 amino acids were taken from the PDB database as of March 11, 2020. The score of the modelling template 4Z8W is drawn in yellow color.

MAESTRO scores for PAC domain models in relation to scores of experimentally resolved structures.

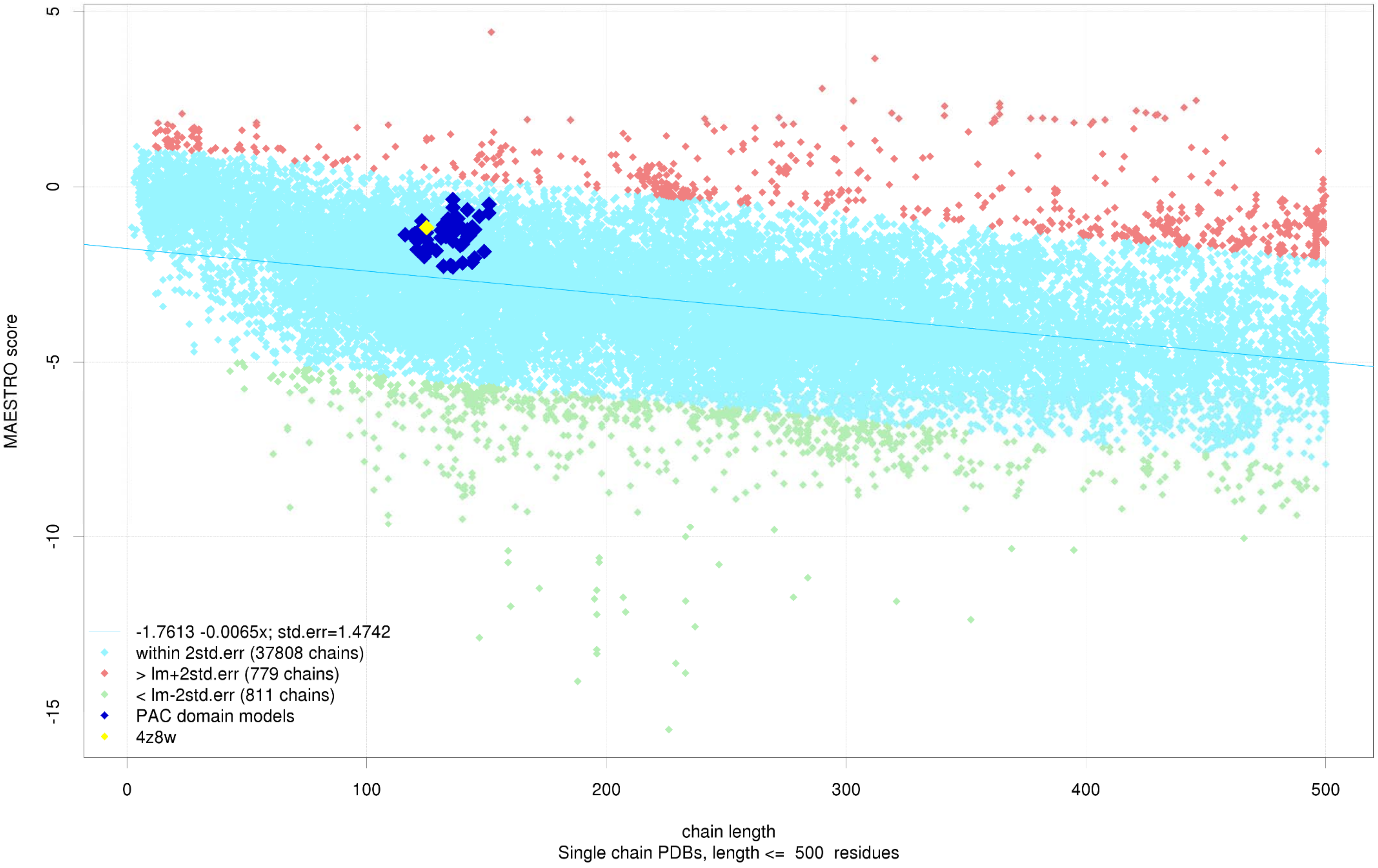

Supplement: Supplementary file 1 [file ijms-21-02488-s001.zip › Supplementary files_R1_Nguyen-Kim, San Clemente et al/Nguyen-Kim, San Clemente et al._Supplementary file 13.pdf]
