# Supplementary material for: The Cell Wall PAC (Proline-Rich, Arabinogalactan Proteins, Conserved Cysteines) Domain-Proteins Are Conserved in the Green Lineage
Source: Int J Mol Sci. 2020 Apr 3;21(7):2488. doi: 10.3390/ijms21072488 (PMC7177597; doi:10.3390/ijms21072488)
Supplement: Supplementary file 1 [file ijms-21-02488-s001.zip › Supplementary files_R1_Nguyen-Kim, San Clemente et al/Nguyen-Kim, San Clemente et al._Supplementary file 2.pdf]

Supplementary file 2. Amino acid sequences of *bona fide* PAC domain proteins.

Originally, the accession numbers of the onekp database were of the following type: #onekp:WZYK_scaffold_2017325 Bazzania_trilobata. They have been simplified for more convenience, as follows: >YFGP_2017325_Bazzania_trilobata.

In the protein name, “.nd” means that the type of protein could not be determined because the amino acid sequence of the protein is not complete; “.1”, “.2”, “.3”, “.4” refers to the type of protein (see Figure 2).

Sequences in blue were not retained for the phylogenic analysis because their percentage of identity with other PAC domain sequences of the same plant was higher than 85%.

Sequences in violet were not retained for phylogeny analysis because in some cases, only one/a few species were selected for a given plant family.

Sequences in brown were not retained because they were incomplete and it was not possible to predict either their sub-cellular localization or the presence of additional functional domains.

Marchantiophyta

>Mapoly0014s0128.2
GNVFCDQCMKNTVFPFALPMLGAKVSVECKAADGSMMASAEATTDFLGGFVASFKGMADLEGCKAY
MKSSPDSSCSIAGYEGGGELKLRSKFFFTSFYGVEPMFFKPAAPKSFC
>WZYK_2017325_Bazzania_trilobata.2
GSVFCDECLKQEAFPFGVPLKDATVKIQCYDGTGANVVASATATTDFFGGYGATFKGVSDLGDCQAF
LANSPDTSCNLSEGGGKTLDLKSKFLSDAFYIVDPLFYKPVQPKDFC
>WZYK_2010801_Bazzania_trilobata.1
GCVYCDNCAIGFETPVSTHIAGAEVKLECRDRHTHEVVYEAFATTDKAGNYEIPVKEDQRTRVCDTVL
VKSPMKGCSKADPGRDRSRVTLTSFNGMISSYRYANNMGFLRDEPLAVC
>RTMU_2181156_Calypogeia_fissa.2
GSVFCDQCLTHEVFPFSYPLKDARVSVQCYDSSGVHVVASATATTDFFGGYIATFKGVSDLGGCKAF
VSSSPDSSCNLVDGGGKTLSLKSKFLWDAFYIVDPLFYKPAHPKDFC
>ILBQ_2004952_Conocephalum_conicum_2
GNVFCDQCMKNEIFPFALPMHGAKVSVECMGADGRMAASAEATTDIFGGFWVSFKNAENMDGCRV
YMKSSPDSSCSMVGDQSGRAVQLKTKLFFSSFYAVEPMFFKPPAPKSFC
>CHJJ_2136182_Lejeuneaceae_sp_2
GSVFCDQCLKQAVFPFGLPLKGAKVTVQCYDASGKIVASGGATTDFIGGYAVTFKGASDLTGCKAFAT
SSPDSSCVLTESSGGQAVALKTKFIHNAFYVAEPLFFRPPQPKNFC
>TXVB_2074716_Lunularia_cruciata.2
GNVFCDQCMKNAVFPYARPMLGAKVSVQCYGADGSMLASADASTDFLGGFLASFKGVSDL
EGCKVFMKGSPDSSCNFADQSGQGVKLRTKFFFSSFYGVEPMFFKPPTPMGFC
>TFDQ_2117810_Monoclea_gottschei.1
GKVYCDNCAAGFPTRISPPIPGATVAVECKDNAGKRSFYNEDRTDDKGLFSIAVKGEHEREACEAYTV
SSPTSCNIQSDSSRGPVYLTHNNGIESDERKTGPFAFKSSQIHSAC
>TFDQ_2004419_Monoclea_gottschei.2
GKVFCDTCLEHKLSEHGYVISGASIAVECDLNRKAMSVSVTGETDENGEFKVEIPSTILQLADHELNKC
RVISRNSPYDSCKIPSTSAPSKLTLQSDSNGVLTYSARPFSYRHETVPPSC
>TFDQ_2013818_Monoclea_gottschei.2
GNVFCDTCFQQQFSEKSYFISGASVAVQCRINKQQKPYFHIEAKTNEYGEFKVELPPSIFYSHEQVKR
CSAKLLKNPQGSCKVASMAKSSSFSLKSKQGGIYTYSAGPFTYRPPSLPPSC
>YFGP_2104693_Pallavicinia_lyellii.2
GSVFCDQCLKHEVFPFGLPLNGAKVSIQCYGASGANVVMSSEATTDFLGGYVATIKGVSDLGGCKAV
VTASPESSCNLVDGGAQALVLKTKFFFNAFYVVDPLFYMPAKPKDFC
>UUHD_2145467_Porella_pinnata.2
GSVFCDQCLKQEVFPFGLPLRGAKVSVQCYDGTGVNVVASAEATTDFFGGYAATFKGASDLGGCKA
FVTSSPDSSCSLIEGGGLTPVVKTKFFFNAFYIVDPLFFKPPQPRDFC

>HPXA_2020358_Ptilidium_pulcherrimum.2
GGVYCDQCLKQEIFPFGLPMNDAKITVECYDGTGVNVVASATATTNFLGIYETSFKGVSDLSGCKAFV
TSSSQSSCSLAEGNGQTLDLKTKFFNNAFYSVDALFYKPAQPKGFC
>WJLO_2035800_Riccia_berychiana.2
GNVFCDQCMKNTVFPFALPLHGAKVSVQCAGADGKMMAAADANTDIFGGFLVTFKGVEDMGGCRV
YMKSSPDSSCNIVGNQGGLAVQLKTKLFFTSFYGVEPMFFKPPAPKSFC
>IRBN_2161692_Scapania_nemorosa.2
GSVFCDQCLKQEVFPFGRPLKGATVSVQCYDGAGVNVVASATATTDFFGGFIATFKGVSDLGGCKAV
VSSSPDSSCSVSDGGGQTLALKSKFLNHAFYFVDPLFYAPAKPDDFC
>HERT-2003915-Sphaerocarpos_texanus.2
GNVFCDQCMKNAVFPYALPMLGAKVSVQCYGAGGSMVASADASTDFFGGFVATFKGVSALDGCKV
VMKGSPDSSCSINADSGGKALELKTKFFFSSFYGVKGPMFYKPSAPKTFC

Bryophyta

>Pp3c1_27500V3.2
GKVFCDRCIQNKVFPFAHPMSKAKVSVQCKDWSGRVVGYARTSTNFLGDFIVQFKGKKDLSGCSVYL
DGSSDRSCNIIGGGRRAISLKSKFLFQAFYVADPLFYKPARPMGFC
>Pp3c14_8250V3.2
GKVFCDQCIQNKVFPYAHPMSKAKVKVECKDASGNVVDYADASTNFLGDFIVSFKGKEDLTGCSVSL
AGSPDSKCSIVGGGGKTVTLKSKFLFKALYVVDPLFYKPALPMEFC
>Sphfalx0052s0052.2
GSVFCDQCLQDRVFPFSEPMDGAKVTVACKDNNGNVVDSVQGSTDFLGNFLVTFKGKQDLSGCTVS
LAGSSRSDCNIAGGGGKTLTLKSRLLFEALYVVDPLFYQPAKPLDFC
>WNGH_2084316_Aulacomnium_heterostichum.1
GRVYCDTCRAQFVTKLSEYMEGAKVRLECRNREGGDLTYSVEGETDTTGTYQLGVDGEHENEVCEI
VLVKSSKPDCSEISTDSYTRKASRISLTSNNGIVSGKRLANALGFMKKEALPEC
>BGXB_2078424_Plagiomnium_insigne.2
GKVFCDQCIQSKVFPFAHPMNKAKVSVQCKDQSGKVVDSAQASTNFLGDFMVSFKGKEDLAGCYVY
LSGSADSKCNIVGGGGKTLSLKSKFFFKAMYVVDPLFYKPAKPMGFC
>SKQD_2012127_Takakia_lepidozioides.2
GSVFCDQCIQGRVFPFSYPLDGAKVTVQCKDAYGRVVDSAQGSTNFLGDFLITFKGKEDLSGCQAILS
GSPEAKCNIVGGGGRTMTLKSKLFFQALYVVDPLFYKPAQPAGIC
>YWNF_2047246_Hedwigia_ciliata.2
GKVFCDQCIQNKVFPFAHPMHDAKVSVKCKDKYGKVMDYAQASTNFLGDFIVSFKGKEDLSGCYVYL
SGSSDRKCNIVGGGGKTLNLKSKFLFKSVYVVDPLFYKPWKPMGFC
>TMAJ_2023569_Neckera_douglasii.2
GKVFCDQCIQSKVFPFAHPMNKAKVSVQCKDKSGKVVDYAQASTNFLGDFMVSFKGKEDLSGCYVY
LSGSSDRKCNIAGGGGKTLSLKSKFLFKAMYVVDPLFYKPATPMGFC
>WNGH_2083088_Aulacomnium_heterostichum.nd
GKVYCDTCRTQFVTKVSWFMKGATVKIECRDREGGSVTYSSQAETNEKGAYTISVDGEHEEEICEVV
LVKSSDPECSEVSKDPFLKKSARVSLTMNNGIVSPVRLANPLGFMKKKPIPEC

Anthocerotophyta

>RXRQ_2140319_Phaeoceros_carolinianus.4*
GHVFCDQCRQGLSSQLTVPLQDAEVGVTCVDVYGFVTYNAVAYTSPDGSFKIPLDDLPPLYGVKGCK
AHLVASGDFNCNLASSVGGGAAGAKLVLNSRSLEEVIFTSGPFAYHPSRPLRLC
>RXRQ_2139239_Phaeoceros_carolinianus.4*
GHVFCDQCKMGLNSLFTVPLQDAEVGVTCVDVYGFVTYNAVGYTSNDGAFQIPLDDLPLIYGVKGCK
AHLVSSSDFQCNMASSVGGGNSGAKLVLNSRSLEEVIFTSGPFAYRPSKPLLVC
>IQJU_2004004_Anthoceros_formosae.nd
GTSECLDCAEKNLKAEDAYQGLQVAIKCKTSNEDYIIISSDKLDKNGNYKVQLPTELIGDDGEPKQECF
AQLHAVTNAPCPVKNGLSPTKLVLKSKKDGVHAFSAATKLQFSSTTC
>IQJU_2076581_Anthoceros_formosae.nd
GRVYCDTCRAGFETTKTAHIAGARVKVECRDRKTMDLVYSKEGTTDSKGSYSIFVDEDHEDQLCDAV
LVSSPQKDCSTPSAGREHARVVLTRSNGIVSGDRYANAMGFMKDEAEPGC

Psilotales

>ALVQ_2002139_Tmesipteris_parva.2
GRVYCDVCAAGYFTKSSSYIQGAQVAVECTSKDGTPTLSVQGKTDSDGVFEIALHGDHQTELCAARL
VHSPSESCNALTDRAASPVYLTRHAGLAVNVIATGPFSFKPQQPLSA
>ALVQ_2024672_Tmesipteris_parva.2
GSVYCDQCLDGRRSVWSLPLDGAKVTVECNNSKGQEILSREGDTGLFGDYVIRFDGSPNLGGCAVR
VLSSPKRTCNIVAIGSKSISLSWKLFGLSSYSADALSFRPSKAMSFC
>ALVQ_2015603_Tmesipteris_parva.2
GTVFCDQCIQQRFTKHSAILHGASIVIECKPVKSTGGTVRAEILTDEHGMFKAVIALSPRRVRQCIVRLV
SSPIKSCSAQPSNPSTFTSASLKSKQGRRYFYNAGFLSYRPDSPPPSFC
>ALVQ_2015640_Tmesipteris_parva.2
GVVYCDRCLEHTYTKNGVLLKGASVAIDCKDKDHDAPYRVVALTDESGSFKVRLSPSLHHSEAQLDC
TAHLLSSSSSQCKVLGISTKKSSDFLHHSRDADHVIYTAGDFTFLPQAVPSFC
>ALVQ_2102199_Tmesipteris_parva.2
GSVYCDQCVEGHRSIWGLPLDGAKVAVECKNSNGQISLSKQGTTNILGLYSVEFDGSPGMEGCGVR
ALSSPSGSCNISAVASQPVSLSWKMFSLAFYSGNALSYKPSKPMAFC
>ALVQ_2000815_Tmesipteris_parva.4
GQVYCDICRTGKFKKPLMGVKIGVLCWSGKEQLSFNGSTSITGRFRIELQGYDYAKWGGEHCKSKLM
EAQPGTSCTIPTNLHDGEAGAKLHIKSKSEAETVLETGPFAYVSHKKHKQC
>ALVQ_2022237_Tmesipteris_parva.nd
GHVYCDQCGEGLWGRMTVPLEEVEVSVKCKDLYGFVTFSAIGYTNDHGVYKIPMDEIPSIFGVEGCK
ARIMRSTNPFCEVPTSLSHGDSGARLELKSKTIKEVVFISGPFAYKPFERPSKC
>QVMR_2006336_Psilotum_nudum.2
GSVYCDQCLDGHRSVWSIPLDGAKVAVECNDSKGQKVLSREGETGLFGDYAIRFDGSPHLGGCVVR
VLSSPKRTCNIAATGSKSISLSWKLFGLSFYSVDALSFRPSKAMSFC
>QVMR_2012184_Psilotum_nudum.2
GSVYCDRCIEGHRSVWGLPLDGAKVAVECKDSSGQMSLTKEGTTNMLGLYSVEFDGSPEMEGCGV
RALSSPSESCNVAGVTSQPVSLSWKLFSMAFYTGNALSYKPSKPMSFC
>QVMR_2014545_Psilotum_nudum.2
GSVFCDQCAQQRFTKNSSILHGAIIAAECKAKRSTVRTVRVTATTDQNGMFKLEIPTTIARSPRRLRRC
VVLLVSSPIESCSAQPSIPMPFSSMSLKSKLGNHYFYSAGFLTYTPDSPASFC
>QVMR_2053533_Psilotum_nudum.nd
GRVYCDTCAAGHFTKSSTYLPGAHVAVQCTSAEGRQTVHAEGKTDANGVFAIEVAGDHQNELCVAA
LLRSNSASCSALSKRAIARVYLSRYNGLSTNVINTGPFSFKPEQVSAAC
>QVMR_2000503_Psilotum_nudum.nd
GLVYCDACKDGKYKKPLTGIKVVASCLDGSNQTSFDGITDSTGRFRIELDGYDYQKWGGKNCNVKLA
EAPPSCNIGTGLPSGETSAQLHIKSTSKDEVVLESTPFAYVSENKFDEC
>QVMR_2054588_Psilotum_nudum.nd
GRVYCDVCASGYFTKSSSYIQGAQVAVECTSKDGTPTLSTQGKTDSDGVFEITLHGDHENELCAARL
VHSPSESCNVLTDRAVAPVHLIRNAGPAANVITTGPFSYKPQEPVSAC
>ALVQ_2022237_Tmesipteris_parva.nd
GHVYCDQCGEGLWGRMTVPLEEVEVSVKCKDLYGFVTFSAIGYTNDHGVYKIPMDEIPSIFGVEGCK
ARIMRSTNPFCEVPTSLSHGDSGARLELKSKTIKEVVFISGPFAYKPFERPSKC

Equisetales

>JVSZ_2007333_Equisetum_hyemale.1
GRVYCDACGAGFFSKSSRYMAGAHVVVECTSKGGEKTLKAEGVTNAEGVFQIEVAGGHEEDSCTTG
VVRSESNACSSFSAKTFTSVYLTRNNGIANNIVNTGPFSFRPDQVASSC
>JVSZ_2123642_Equisetum_hyemale.2
GTVFCDQCHAQRLTLDSIFLPGTTISIQCVARRTNAQRFSYIHLKTRTNKFGQFKLKFPPDFSGEQIQG
CTLELVRTPFKECNGILGYSTKNSLKFVLKKNGAYFYTFGAIGVKPKVAPSAC
>E.giganteum_11472_Transcript_23135.2
GYVYCDQCLNLKLTDNAIFLQGASVFIKCQDPQGYNWNTIIATTNEKGYFQAKLHADFNPTCYAYIESS
PNAECNSMGLNMKNSLDFELTNSIGNNVTYSIGEFTFIPSIIPSLC
>E.giganteum_7703_Transcript_16468.2
GHVYCDQCLDHNRTNNAIFLKGALIFIKCKDPERYHWKTIMATTNEKGYFQAQLHGDFNPTCYAHIKS
SPSTLCNVKGLNMKKSSNFEFSYSIDNNVTYSLGEFTFTPSILPSLC
>E.giganteum_18690_Transcript_33322.2
GNVYCDQCLDHNLTNNAFFLQGASIFIKCKDTEGYNWKIIIATTNEKGQFQAKLHGDFNPTCYAHIKSS
PYSLCNEKGLNLKKPSDFKLVYLVDNNATYSVGEFTFTPSILPSLC

>E.giganteum_9519_Transcript_19781.2
GYVYCDQCLNHNLTNNAFFLQGASIFIKCKDSEGYNWKTLIATTNEKGHFQAKLHGDLKPICYARIKSS
PDSFCNVKGLNMKKPSDFKLSHVVDNNATYSIGEFIFTPSILPSLC
>E.giganteum_2589_Transcript_5803.2
GNVFCDQCIQHKLTKNAIFLQGVSISIKCKDHEGYNFKTIIATTNEKGYFQANLQGDHAISTCYASTQSN
PNVDCNLMGLNMKKPLDFELAHSNGNNLTYSLGYFTFLPLNVPSFC
>E.giganteum_7962_Transcript_16915.1
GTVFCDKCHENRVTHNSIFLPGATISMQCIAKRMHKHHSSHIHLKAKTDKFGRFKADLPPHFKAKRMQ
GCTLQLVRSPFESCNIFSKHLTSPSLLYISKHKGTSFFTLGAIDVQPKVRPIAC
>CAPN_2037336_Equisetum_diffusum.1
GVVFCDQCHQQRLTSNSIFLPGSSVSINCIASRMNTNHSSHINLKAKTDTYGQFMMKLPPSFSTNNVQ
GCKLELVKSPFKECNIFSQLSTTNSFTWAAKHEDADFYTFGAISITPKVVPNAC
>CAPN_2004677_Equisetum_diffusum.2
GHVYCDQCLNHNLTNNAFFLEGASIFIQCKDTKGFNWKTIIATTNEKGYFQAKLHGDFNPTCYAHVKS
SPNTLCNVNGLDTKKSSDFKLAYSVDNNVTYSVGEFTFTPSSVPSLC
>CAPN_2038971_Equisetum_diffusum.1
GTVFCDKCHENRVTHNSIFLPGATISMQCIAKRMHKHHSSHIHLKTKTDKFGRFKADLPPHFKAKRMQ
GCKLQLVRSPFESCNIFSKHLTSPSLMYISKHKGTSFFTLGAIDVKPKIRPNAC

Marattiales

>NHCM_2008486_Angiopteris_evecta.1
GKVYCDACAAGHFTKSSTYLPGAHVGVRCASPQGVETAYVEGATDKNGLFQIQVTGDHERDLCVAA
LLRSPSATCNVLTQKTLDHVQLTRNNGLATNVVNTGPFSFKPAQVSAAC
>NHCM_2064417_Angiopteris_evecta.2
GSVYCDQCLNGHRTMWSSPLNGAKVMVECKNSNGQVTLSKQGETDLLGMYSIKFDGSPDMQGCTA
KVVSSPKGPCNLAGTASNSVTLSWKLFGLAVYAAEALSYKPAKPSSIC
>NHCM_2003839_Angiopteris_evecta.2
GRVYCDACDAGRFTKSSSYLRDAKVAVQCTSREGAETVYVEGRTNRDGVFKIKVAGEHEHDLCVAA
VLQSSSQACNLLTDKSMATLLLTSKKLANAVVQTGLLSFKPLQLSSAC
>NHCM_2007797_Angiopteris_evecta.nd
GGVFCDQCSERRFTRNSAILSGATIALECKSRKIHRTAFRVETSTDQRGIFKVNLPSSALRSLRSCTLQ
LLSSTVESCSAHVSMAPVSTSPLIAPKSKHGGRYIYNAGWLTFHPSSLPSFC
>UXCS_2010798_Marattia_sp.nd
GKVYCDACAAGHFTKSSTYLPGAHVGVRCASPQGVETAYVVGATDKNGLFQIQVTGDHERDLCVAA
LLRSPSATCNVLTQKTLDHVQLTRNNGLATNVVNTGPFSFKPAQVSAAC

Polypodiales

>VITX_2095101_Blechnum_spicant.1
GRVFCDACGAGHFSKSSFYLAGAKVGVQCGSGQVRGHVTAYAEVKTGPDGEFSIPVAGEHENEFCV
ALVLHSAHPTCNLLSQRGAFATVLLTRNAGLADNVINTGPFSFAPNRLATSC
>VITX_2014902_Blechnum_spicant.2
GKVFCDSCHVGRLTESSSVIEGADVALRCTSPTGEETAYVEGKTDKAGLFSIHVAGHGTSDACVAAVL
RSSTSACNVLTLQSEAAVNFNGVAEGEILAGPFSFKPSKMSAAC
>AFPO_2010170_Blechnum_spicant.2
GKVFCDSCHVGRLTESSSFIEGADVALRCTSQGGVETAYVEGKTDKEGDFSIQVAGHDTSDACVAAL
LRSSTSACNVLTVQTAAAVFFNEEADGEILTGPFSFKPSKKSAAC
>VITX_2011943_Blechnum_spicant.2
GSVYCDQCLNGHRTIWGLPLNDAKVLVECKDSNGKVTLSKEGSTNMIGAYAISISGSPKMDGCSTQL
VGSSPRSSCAVAGVSTRPVELSWKLFGMAMYTAEGLFYKPSKPMSFC
>AFPO_2008830_Blechnum_spicant.2
GVVSCQDCKYVGTKSLTDAKPLAGAKVKLVCQAGPRYKTFYTTATTDENGFFFLRVPHFDFHKFTPL
DDCAILLLSSPLRECSNPTNINYGKTGAPLRPIPTFAPSSEAFFSVGPLAFAPQPC
>AFPO_2068240_Blechnum_spicant.2
GVVLCQSCKFVGSGSVAHATPLPGAKVKLACQTGFRYNTFFSATARTDYNGFFIVRVPSFDFKTFSPL
KDCVVSLVYSPYAQCSTPTDINYGISGAPLC
>VITX_2005384_Blechnum_spicant.4
GNVICELCHKKEALEGAKVGVICQNGESELSFYDTTNGKGGFRIELVGFEYRKYGGNACKAKLMAAS
SENPCNTPTTSSMRHLHIKSKSDTEVVLFAGPFSFTSHSIKC
>VITX_2093320_Blechnum_spicant.4

GNVYCDICKNGNIKKPLQGVKVGVFCWSGKSKKTFYGITNERGAFKILLTGFDYEKWGGASCQAKLIS
PSYATSCTLPTNLHGGKQHAHLHIKSKSPNEVVLLAGPFSYTSPLTHLKC
>VITX_2098387_Blechnum_spicant.4
GHVDCHLCHEKKHLAGAKVGVSCWNGDSEVLFYGVTNDKGNFRIELDGFEYRKYGGKACEAKLIAAP
YDSPCSTPTSSTAAHLHIKSKSDIEVVLFSRPFSFTSTLKC
>AFPO_2066516_Blechnum_spicant.nd
GLVLCQNCNYVGTSYYSYGKPLQGATVKITCESGDKSSFEAKAETDDHGYFLIEVPYFDFEKYYVVDD
CKMYLVSSSTSTCNTPTDINSGISGSPIRLQNTSTDKSLYSAGPLVFAPKYC
>AFPO_2069671_Blechnum_spicant.nd
GVVFCQNCSYVGIGSLTDAQALTGAKVQLACRDWRYKTYLYDTATTDENGYFFLKVPNFNFVKHDPI
KTCRVFLVSSPSPTCNVRTNINSGKFGSFLHKENEFPDQILYSVGPFAFAPPSPC
>VITX_2016183_Blechnum_spicant.nd
GFVVCDSCRENRLSNFSLPIPGATITLTCKITSKHHSTSTPFTTKASSDKYGAFSFDLPFSKPRLLRRCK
VELTSSPFEACIKKTPYSWEVLSPILSLKSKEKGHLAYFSGWLSYQPNILPSFC
>VITX_2095258_Blechnum_spicant.2
GVVSCQDCKYVGSKSLTNAKPLAGAKVKLVCQAGPRYKTTFYTTATTDKNGFFFLRVPHFDFSKFTPL
DDCAIFLISSPIKTCSNPTNINYGKTGAPLRPIPTFAPSSEAFFSVGPLAFAPQPC
>AFPO_2068324_blechnum_spicant_1.
GRVFCDACGAGHFSKSSFYLTGAKVGVQCGSGQVQGHVTAYAEGKTGPDGEFSIPVAGEHEKDFCV
ALVLHSAHPTCNILSHRGAFATVLLTRNAGLADNVINTGPFSFAPNRLATSC

Lycopodiales

>ZZEI_2006869_Phylloglossum_drummondii.1
GRVVCDVCGDSSIGPEDHVLEGAEVAVLCLTKSGEVLNYQAFTNSKGIYSVAETMPESDRWASCLAR
PISSFHEHCTKRGDAHKGVKFTYNMPSGYSHTVKPFLYKPASVPVYC
>ZZEI_2006244_Phylloglossum_drummondii.2
GTVFCDQCLIGEPSIWGPPMHDASVTLDCQNSGNSDQAKTSFIGTFVFKVGGDADLSSCTARLTGSS
SASCSLLGNAEKPLKLDWKIFGESFYSVAHVLLPHSGPTPPLVLPKPPPLSLPPSPFQDPSSC
>ZZEI_2019056_Phylloglossum_drummondii.2
GTSECLDCAEKNVKTELAFKGLHVAVKCKTANGMYETKAIGELDENGSFKVQLPHELLREDGELMKE
ECFAQLHGVSDAPCPGKDGLLDPSKIVLSTSKADEKQAFVAEVGKLSFSSSTC
>PQTO_2078178_Lycopodium_deuterodensum.2
GSVFCDQCLMGKPSIWGSPMHDANVTVDCKNSNNAIQSLHHASTSVLGTFVFTVSGDADLSSCSARL
TGSSSSSCSLFGNAEQSLKLEWKMFGEAFYSVEPMFFKPEKSMPFC
>PQTO_2013256_Lycopodium_deuterodensum.2
GSVFCKQCVQSSLAIIDHFNTPLAGAQVSLRCNDGSNLVGLSSLESTAVTNKKGKFKISLNADYYQKHGAGRCWVEL
LRSPDEQCNIPTGSEREVNSASVSTQLSDSFSNKLIYYVGPFTYKPASC
>GTUO_2011273_Huperzia_selago.2
GMVFCDCCLEGSYTQNAAFLPDVTVAVECKNSSGSEIFAWKAASNRKGSFVLLIPPQETIQKCWIQLLNNPHGVCN
SISKVKEASQFITTSLKSSIGFTVGEFSYQPKSPFSFC
>PQTO_2082336_Lycopodium_deuterodensum.3
GTVYCDTCWDGNFQTETHFLPGVTLIMQCDTIIGEQFFFGDTDAEGHFSITVTSSDYEKWNLDECKVKLLKSSHGLC
NVATDFNNGQSGAETRLESISSTAIILKSGPFSFKPDQPYPEC
>ZZEI_2124425_Phylloglossum_drummondii.4*
GFVLCKDCGPLTTFPLRGATVDVICSDANGTFFSNTATTDAVGHFEDNIPGIAGNIGLIQDCLVTLGSSSVAQCNVKT
NVGNGISGVHLGTGGTKDLYIVGPFAFTTQKC
>GTUO_2005379_Huperzia_selago.4
GKVYCSKCVIKAPRAHHHHEGLKGAKVEISCGDIIAYGYTNKYGHFKIKLKYYDYEKRGIKQCQAKIVN
SANLNKSCNVLATHHKYKSGYPLKLKYAHENKVLLVARSFVYTSNEPDVEC
>PQTO_2010640_Lycopodium_deuterodensum.nd
GTVLCDCCLEGRYTQNTAFLSDVSVAVECKNGSGGENFIWQGVSDRKGNFAIDIPSLGTNQKCWVYVLNNPHGSC
NSKQASAPKGTISSHPLTLQSLHKSSIVYTVGKFSYQPKAPFWFC
>UPMJ_2079990_Pseudolycopodiella_caroliniana.nd
GSVFCDQCLMGKPSIWALPVHDATVTLDCKNGVKENDASTSMIGTFSFKVSGDNDLSSCSARLTGGA
ASSCSEMGSAEQSLKLDWKIFGEAFYSVEPMFFKPDKPMSIC

Isoetales

>PKOX_2004461_Isoetes_tegetiformans.1
GRVFCDRCLEGRYTKAGVFMQGAKVGLECRRSLDGSSETFEGVTDHSGSFRIVLEGLDGLCSLKLTH
SPFSVCHIPSNDDAEFQILPKHLRPPSMLLYAGRFSFRPSLPLPIC
>PKOX_2094197_Isoetes_tegetiformans.2
GSVFCDTCLDNRYTNNSIIFHDANAVLECKNKDGIFQAHAAANTNRRGEFVMELMSQVSIRYCSVKIV
VDDAINECEISSSSHALSLQATRGSFTTYEVPPFIYKPKEPLSFC
>PKOX_2095629_Isoetes_tegetiformans.2
GYAACDRCLDGGFTNNISPLSGVKVQIQCKNNAGRITYSTTQVTDKQGSFSVDLPTSIAQAQAIKNCA
SKVVEALPHPCNNVVGNLQSTPVPLYIKESMKTSHQIYAAGLFMYKPDKAMSGC
>PKOX_2095893_Isoetes_tegetiformans.2
GSVFCDSCLENRFTKNSILLKDAKAVLECRNKDGRLEQHGVAATNDKGEFEMELRQNSSKISIQDCST
RIILGHKTKQCEIASTSQALSLQAIRGGIVTYNVDPFIC
>PKOX_2099470_Isoetes_tegetiformans.2
GRVYCDRCVEGRFSKFSSFLEGAKVAVQCQTAEGKIKSFEGSTNEAGRFSIAVDAHEKDACAVKLLS
SPSSECAIPFTNAASQIELSKTIAAATGLKYGGALSFRPEKAEPAC
>PKOX_2007667_Isoetes_tegetiformans.2
GAIFCDRCFDEALVQHKTFPLSGAQVILVCKDESGKVVSSTHGYSNKNGVFELKTVQNHASECKLQL
GESTDAECKTATNINKGIAGVDVSERGLSSSKHELEYTVGPFAFRPSNPSPSC
>PKOX_2008463_Isoetes_tegetiformans.4
GKVYCDKCYGTDNVHHVTHHHKGLKGGKVKISCGKDVYYGYANKHGIFRIELSPFYHPTQLKGCKATI
VKTSHVVRSCSTPTNYHHGKIGAGLKLKSHSMKEIVLTTGPFKYTSPHPVPEC
>PYHZ_2071985_Isoetes_sp.4
GKVFCDACGHKHVVDMVPLQGARLSLVCGGRMLSYGYSNMDGVFHIMINGLKYLNLNNSSCVATITK
TSHVSKCPIPSEYNNGTKGGRLYFKSYNSKEVLLGAGPFTYVPLNALRKC
>PYHZ_2008147_Isoetes_sp.1
GRVFCDRCLEGRYTKAGIFMQGAKVGLQCRRSLDGSSQTFEGVTDHSGSFRIVVEGLDGLCSLKLTH
SPFSVCHIPSTDDAEFQILPKHLRAPSMLLYAGRFSFRPSLPLPIC
>PYHZ_2072068_Isoetes_sp.2
GSVFCDSCLENRFTKNSILLKDAKAVLECRNKDGRLEQHGVAATNEKGEFEMELRQNSSKISIQDCST
RIILGDKTKQCEIASTSQALSLQAIRGGTVTYNVDPFIC
>PYHZ_2009504_Isoetes_sp.2
GRVYCDRCVEGRFSKFSSFLEGAKVAVQCQTAEGKIKSFEGSTNEAGRFSIAVDAHEKDACAVKLLS
SPSSECAIPFTNAASQIELSKTIAAATGLKYGGALSFRPEKAEPAC
>PYHZ_2012705_Isoetes_sp.2
GTIFCDRCFDEALVQHKTVPLSGAQVILVCKDESGKVVSSTHGYSNKNGVFELKTVKNHASECKLQLG
ESTDAECKTATNINKGIAGVDVSERGFSSSTHELEYTVGPFAFRPSNPSPSC
>PYHZ_2013089_Isoetes_sp.4
GKVYCDKCYGMNNVHHITHHHKGLKGAKVKISCGKDVYYGYANKHGVFRIELSPFYHPAQLKGCKATI
VKTTHVVRSCSTPTNYHHGKTGASLKLKSHSMKEIVLTTGPFKYTSPHPIPEC
>PYHZ_2067647_Isoetes_sp.nd
GSVFCDTCLDNRYTNNSIIFHDANAVLECKNKDGIFQAHAAANTNKRGEFVMELMSQVSIRDCSVKIIV
EDAVNECEISSFYHALSLQAIKESFTTYEVPPFIYKPKEPLSFC

Selaginellales

>S.moellendorfii_429316.1
GFVSCQDCDGNFDKPSPGALVRVECRDGSYGEAASNRDGFFQIKFPQTYKSVYDPPESCKAMILASP
DRSCDTATDLGGGKKGAALEFEKIFMGEAFYKTGPFAFTPYC
>S.moellendorfii_422150.1
GYVFCQNCYKDWSRHLGVHPGAEVSVECTAISTGKLVALGKGVSRENGYVEIRLDGYDYSSKLKIHR
CLAKIVRSRDPSCDYRTNVNSGYSGAKPQFSGVAGNIAYFTTNAFSFTPMC
>S.moellendorfii_409806.2
GYAICDTCWEMQITANSISLPNVTAAVECFDAGGRIIFRETSSTDNTGAFRVELPFQIADSVSMLSTCT
SRVVRGPLNHLCTYPLAHAYNPFLSPRSLPDGRQVYGVGPFVFRPRAKLPHC
>S.moellendorfii_405039.2
GYVVCDACREKNLTSNSVFLSGMTVSLTCKAVGGQRPFFTAQQATDEQGRFSLDIPSQISGSGDGFK
LCAASVAAGEKGITPLQRSCNVAAMEKSHSSLTPYTTIEGHRIFSTGPFVYTPSKSLQSIKSC
>S.moellendorfii_443385.2
GTVYCDSCLEARLTRNSIVFSEAEIQIECVGSRGNPDHSTVVSTNETGQFQAQIPPQFDACSVQFLSK
ALDSCATPAAAFATSASLDQPRDLVFRPSQALAFC

>S.moellendorfii_409805.2
GAIFCDRCMDKTFSFATIAIPNVTAIIRCGDFQASNTTSEFGVFTIALPLEVGTSPAKLASCSVKVASDFS
RVEGDLGTCNTPVKFQFQPWSPPSPPSMPAFQSSLKIARPWPFYSAGPFFFQPPEPLPSC
>S.moellendorfii_430897.2
GNVFCDQCLHGRPSMWGIPIRGAKVLVHCRNRQGMAMAVPAMSNFLGAFVANFPGEHDLQHCTSR
LMWSPLKQCNVVANAQQSLRLEWKILDNAFYTVKPLYFTPVKPLAIC
>S.moellendorfii_443961.2
GRVTCERCSTKHKAKTHGLSGVSVELSCPQANLTMVTQHHGQFSMGFHHLEPLGFPGSCAVSVITS
SLPSTCNVPVGSATNSFTPTLHSTSSKETAYVVPAFEFTNGDNC
>S.moellendorfii_438958.4
RVFCDRCIEGRFSEYAIFVKGAKVALECTQQDGGIHVVEAVTDSAGAFAIPVDEPGSSCAVKLLSTPTP
QCNIAAKHAQTLNANMRTKDNVIYVGLLSFRPEEPLPIC
>ABIJ_2038764_Selaginella_lepidophylla.2
GSVYCDSCLESRLTHNSIVLAEAEIQIKCTGGSGIRDHSAVVTTNQTGQFQAQVPAQFDATVCSAELL
SKPFDTCSIAAPSPTSPSYNRDFVFRPRQALGFC
>ABIJ_2038663_Selaginella_lepidophylla.2
GTVFCDQCLEKTFTFNTIPIPNVTATVECGDFQASNITSEFGTFIIQIPADVATSKLPSCTVKVASDFSSV
QGDLETCTTAVQLQLGFPGSSSSPPSSLKAAQDGWPFYAAGPFFFQPPQALPAC
>ABIJ_2004715_Selaginella_lepidophylla.2
GYVVCDACLEKSLTSNSVFLSGMTVSLKCQAAGGQQPFFAAEQQTDSQGRFSFDVPSQISGSIEVSK
LCSTGIAANKGDTSSLAGSCGSASQALASSSLTLYAAIQGHPIYSTGPFVYTPSQALESKTC
>ZFGK_2039051_Selaginella_kraussiana.2
GNVFCDQCLHGRPSMWAIPIQGAMVVVHCPSRQGVALQRRAYTNFLGAFIASFPTATLQECTSHLVW
SPMSECNNVVGSGRQQSLRLEWKMFDDSYYTTKPLYFKPLSPMSHC
>ZFGK_2040668_Selaginella_kraussiana.2
GRVYCDQCKEGRFSEFSSLLQGAKVNVECNEGRILHQGITNNAGLFSIKVPNALATSGCIVKLVSSPLE
SCSIASHNQQQLIELMKVAKASTSVKYTGPFSFAPENALSFC
>ZFGK_2038534_Selaginella_kraussiana.nd
GTVFCDTCHERHLSESSIIFKDAKVLVECDSSFSEILRTDVKGQYTMQLHRGVTAPKHCKSTLLSSTDS
ALWTSCSTTLSYHLDQSQVSKVQDLIFKPKTSTFC
>ZFGK_2008341_Selaginella_kraussiana.nd
GFVACDACLERNFTKNTVLLPGVTVSVTCKDGNGDPFFSSIERTDKHGRFFVNIPEQLANSEASHITTL
CSSAVVNNPVPSCSIPLTSSLGSSLVPYAMQNGHKIFTSGPFIYSPRQAHNSC
>ZFGK_2039185_Selaginella_kraussiana.nd
GFVACDACHEMKYTKNTAFLPGVTTSLTCKDGNGNPFYSALQITNKQGMFIFDIPSQLVHLNASHLTT
TCSTDIVKVSTRTSCRIPLSSSSSSKLFPFEIKGHKFFITGPFVFKTSKVEQRSC

Ginkgoales

>SGTW_2003638_Ginkgo_biloba.1
GQVFCDTCAVGFITRVSKPIQGAKVAVQCKDHAGKQLYYTEGTTDFQGYFNIPVNGDHSHEKCEAKAI
SSPTSCNIPSYTNIGPVFLTSNNGVVSSERITGPFGFKSTEILAVC
>SGTW_2037819_Ginkgo_biloba.1
GRVFCDVCADGSMGPEDHFLEGAEVAVLCMTKSGEVLNYQAFTNSKGIFTVAETMPESNRWDTCLA
RPISSVYQYCNLLGDFATKFSYSLSSGRSYTVRPFSYQPLKTPMYC
>SGTW_2037953_Ginkgo_biloba.1
GKVYCDTCAAGFVTRVSTYIPRARLAVQCKDHVGKQLSYTEGETDQSGTFRIPVKGDHAHDLCETVA
LSSPTSCNIPTDSNRGPVFLTHNNGINSDERITGPFAFKSTQTLAAC
>SGTW_2038568_Ginkgo_biloba.2
GNVFCDQCRDGHRTMFDLPINGAKVAVECTGSDGQITMSKQETTDLIGNFMMKFDGTPDLSGCTAR
VTDSPQDSDCKIVGSSSKPLSLSFRLFSMEVYTVESLFFQPAKPMSFC
>SGTW_2037611_Ginkgo_biloba.2
GTVFCDTCFEQQFSKSSYFISGATVAVECRIKKHGPSFRVEAESNEYGEFKVELPSSTFHSEKQVKRC
SVRLLRNSESSCKVASIVKSSSLTLKSKQGGIHTYSAGSFTYRPQSPPALC
>SGTW_2037362_Ginkgo_biloba.2
GSVKCHDCSEQRLDKRLALSGASVAINCKTGQEVSTRGVGIIDETGSFSVKLPSSVLNEKGELGQPCF
AKLVSGNKKLCSFTNSAPSKLVFATEEGKKNVLTTSDELSFSSLSC
>SGTW_2033762 Ginkgo_biloba.nd
KVYCYYCWDWSNPKHSHGKHHLKGATVVITCKAGYKEITAYGVTKDNGKFKALVEGYDYGKWGGAE
NCKAKLYKPPPHSSCNIPTNLHGGKTGAWLKVKSKSEEEIVLIARPFAYAPAKPYAEC

Cycadales

>GNQG_2082130_Encephalartos_barteri.1
GRVFCDTCNAGFVTRASTYIADAKVAVECKDHVGKRLTYKEGVTDADGSFRIPVAGEHVHDLCEAKA
LSSPTACNIPAYTNTGPVFLTHNNGIASNERTTGPFGFRSGSTLAVC
>GNQG_2084485_Encephalartos_barteri.1
GQVYCDTCAAGFVTRVSTYISGAKLAVQCKDHAGKLLSYAEGQTDGSGTFRIPLKGDHSHELCEATV
LSSPAPCNIPTASNRAPVYLTHNNGIDSDERATGPFGFKSTETLAAC
>GNQG_2012735_Encephalartos_barteri.1
GRVFCDVCADGSMGPEDHFLEGAEVAVLCMTKLGEVLNYQAFTNLKGTFTVAETMPESNRWETCLA
RPIGSVYRYCNILGVNNSAIKFTYSLRSGNSYTVGPFSYQPSKAPMYC
>GNQG_2079100_Encephalartos_barteri.2
GVVTCDDCSEKNGKSGVGLSGASVAIECKNSRGDRLHTRAEGSIDENGAFSVNLPQHILNAQGELQE
ECFAKVRSGSKRPCPAADVAATKLVLQSKRDGTNIFSPSDRLSISSSSC
>GNQG_2008332_Encephalartos_barteri.2
GSVFCDTCSDRQFTESSYFISGATVAVECRVKSPARWIRIEGKTNQYGEFQVQLPSSNFRSGGAESL
KRCSARLLPNPRGSCKTASIGKSSSLALKSKKGGIYTYSAGALSYRPQSTPPMC
>GNQG_2015302_Encephalartos_barteri.2
GRVFCDTCSEHQFSETSSYYLPGALVAVECSINRKATSTVSIEGETDEYGEFRVEIPSIFQPLRRCSVR
MLSSPERSCSIPSTTTSFPLRFTKSKSKLNVNFRGDRTYNAGSFSYRPLNAPSAC
>GNQG_2082056_Encephalartos_barteri.2
GSVFCDICLQHRFSETSYFLPGALVAVECSLNRKATSVSSEGETDEYGDFRVEIPSIFHAEDRVNRCS
VRLLSSPEGLCNVIQSASTPRPFTLTSNFDGVRTYDAGSFSYRPKSVDPRLC
>GNQG_2085018_Encephalartos_barteri.2
GNVFCDQCLDGLRTMFDLPLYGAQVRVDCRGDDGEITFSKEDSTDLSGNFVVKFDGIPDMSGCAAQ
VIGSPRGSDCNIVGSPARPLDLTFRLFAMEVYSVDPLFFHPAKPMPFC
>GNQG_2084302_Encephalartos_barteri.2
GSVECDGCLEQNLDRRLALSGSSVAIKCKTGKEETTIHGVGSVDENGSFSVKLSSSALNERGELAFPC
FARLVSGNQKLCSFANTPSSKLVFKSPHTLTTSEVLSFSSSSC
>GNQG_2008166_Encephalartos_barteri.4
GKVYCYECWDWSNPKNSHQKKHLGGATVVVMCKTGDGKTTEAYGVTENNGKFKVIIDGYKSGKWE
GAENCKAKIHKAPKSSLCNLPTNLHGGKTGAWLKVKSKSPEEIVLIAKPFAYAPAEPYAKC

Araucariales

>IZGN_2070361_Dacrydium_balansae.1
GRVFCDVCADGSIGPEDRFLEGAEVAVLCMTISGEVLNYQAFTNLKGIFTVAETMSERSRWDMCLAR
PISSIDQDCNILGNNNAATKFSYTLSSGHSYTVRPFSYQPVKTPMYC
>IZGN_2069360_Dacrydium_balansae.1
GSVYCDNCHAGFPTRISPAIAGAKLAVRCRNQAGAETVYTEGVSDENGEFKISVKGEHDQDFCETVA
LSSPTTCNIPSESNHGPVFLTHNNGINSDERITGPFAFKSAQKNSEC
>IZGN_2069612_Dacrydium_balansae.1
GKVYCDNCHAGFPSRISPSIGGAKLAVRCKNNVGTQTLYSEGVSDSEGQFEISVKGERAGEFCETVA
LSSPNRECDIPTDSNHGPVFLTHNNGIPDDTRATGPFAFKSTEKHPEC
>IZGN_2072474_Dacrydium_balansae.2
GSVFCDKCFRQEFSKHSYFIKGAPLAVECGTGKEGLNFRTEANTNEHGQFEVELPASVFHSETHLKR
CSAMLLQKEGPCGVTSMANASSFTLKSKQGGIHTFSAGFFTYRPQTIPPSC
>IZGN_2003971_Dacrydium_balansae.2
GSVFCDTCFRQEFSKSSYFIKGAPVAVECESGKGGPNFQIEANTNEHGQFEMELPEFEFHSETHLKR
CSARLLQKKGPCRMASIANSSSFTLKSKQGGIHTYSAGFFTYRPQTIPSSC
>IZGN_2009542_Dacrydium_balansae.2
GKVFCDTCLQNHFSNTSYFLPGAVVAVECKISRNPPTIIAEESTDENGEFVVQITPPSIKTTNTNNMTGL
RKCWVQLVRSPQSSCNVPSITKSSQIGLALDFNGILTYESPSLTYRPQDLPTSLC
>IZGN_2072084_Dacrydium_balansae.2
GNVFCDECQDGRRTLFDLPVNGAKVAIECNGQSNQVTTDLVGNFVMKFDGIPDLSGCSARVVQSPP
GSECNIVSSPAKPLTLTFSLFGMEMYTVESLSLEPAKPMSLC
>IZGN_2000214_Dacrydium_balansae.4
GKVYCYYCWDWSNPKLSHGKHHLKGATVVVSCKAGYKEITAYGVTKDNGKFRVQVEGYDHEKWGG
AENCQAKLFKPPTDSHCNIPTDLHDGKTGAWLKVKSKSEEEIVLYAKPFAYAPAKPYAEC
>IZGN_2068225_Dacrydium_balansae.4

GKVDCYKCWNWKQPNASYDKGGLEGAEVVVKCKAGEETVTAYGVTIKHGKYKVVLKGYGYTKWDIA
KSCTAKLYKPPKNSTCSLPTNLNKGISGAMLKFKSKSQKAIVYSVKPLAYAPSKPYSKC
>IZGN_2069230_Dacrydium_balansae.nd
GRVFCDTCFHRKLTTTSHFIPGASVAVGCNIKRDGVKSKAEGKTDERGFYRIKIPVHFRYEKDIEKCSV
ELVRSSIIWCSVPFIRNRSRLVIKSKIKDGYLYRADPLSFRPPRLPAFC
>IZGN_2005472_Dacrydium_balansae.nd
GSVFCDTCSHHSLHSPTTYFITDATVAVECSLNRKARALIRVEGRTDESGEFKIELPSFFYNTYSGCSV
EVINSPDGLCNVPSIGKLAFTLNWRQPATVYGAGHLSYRPELTPSAC
>JRNA_2010185_Phyllocladus_hypohyllus.1
GKVYCDNCHSGFPTRISPSIAGAKVAVQCKTREGKQTLYSEGVSDSEGQFEISVKGDRAQELCETVA
LSSPTRECSLPSESNRGPVFLTHNNGIPDDTRITGPFAFQSTEKHPQC
>JRNA_2064064_Phyllocladus_hypohyllus.1
GRVFCDVCADGSMGPEDHFLEGAEVAVLCMTISGEVLNYQAFTNSKGIFTVAETMSESSRWDMCLA
RPISSIDQDCNIPGNNNSASKFSYALSSGRSYKVRPFSYQPVKTPMYC
>JRNA_2003839_Phyllocladus_hypohyllus.2
GRVFCDPCLHHQFPSDTAHFLPGAVVAVECSTINNRKLRITILSNKAETDEYGAFRVEIPSGIHNLNLNP
ITSCSVRLLNSPHQSCNIPSISTSSLITPSNASGLETIYTSAPLSYRLQDIPDSLC
>JRNA_2012549_Phyllocladus_hypohyllus.2
GRVFCDTCFHQKLTTTSYFIPGASVAVGCKLRGNGKAEAKTDERGFYRIKIPLRFRSERDIEKCSVKLL
RSSIIWCSIPFIRNRSRLSIKSKIKDGYLYRAAPLSFRPSKLPAFC
>JRNA_2066102_Phyllocladus_hypohyllus.2
GNVFCDECQDGRRTLFDLPVSGAKVAVECNGQSNEVTTDLVGNYVLKFDGTPDLSGCSASLVQSPP
NSECNIVGSPAKRLSLTFSLFGMEMYSVESLSLQPAKPMSFC
>JRNA_2006993_Phyllocladus_hypohyllus.2
GSVFCDTCFRQEFSKSSYFIKAAPVAVECESEKERPNSRIEVKTNEHGQFEVELPASLFHSETQVKKC
SARLLQKEGPCRVTSMANTSSFTLKSKQGGIHTYSAGFFTYKPQIIPASC
>JRNA_2006992_Phyllocladus_hypohyllus.2
GSVFCDTCSRQEFSKSSYFIKGAPVAVECESEKERPNSRIEVKTNEHGQFEVELPASLFHSETQVKKC
SARLLQKEGPCRVTSMANTSSFTLKSKQGGIHTYSAGFFTYKPQIIPASC
>JRNA_2001594_Phyllocladus_hypohyllus.4
GKVYCYYCWDWKNPKLSHGKHHLKGATVVVSCKAGEKDITAYGVTKVNGKFKVQVEGYDHEKWGG
AKNCKAKLLKPPTGSACKLPTNLHYGETGAWLKVKSKSEEEIVLYAKPFAYAPEKPYAEC
>JRNA_2003193_Phyllocladus_hypohyllus.nd
GVVKCSECTQKQQNAGHEASPYQGAFVGIQCKTSKGEVHESGVAIIDDNGSFKMNLPQYLVNDKGEL
AGECHAKLLTRYSLKPCSTNGDGDMSSKLVLQTKSKESESDVDTIIFGTSSDLSLSLSTC
>JRNA_2003196_Phyllocladus_hypohyllus.nd
GVVKCSECTQKQQNAGHEASPYQGAFVGIQCKTSKGEVHESGVAIIDDNGSFKMNLPQYLVNDKGEL
AGECHAKLLTRYSLKPCSTNGDGDMSSKLVLQTKSKESESDVDTIIFGTSSDLSLSLSTC
>JRNA_2003194_Phyllocladus_hypohyllus.nd
GVVKCSECTQKQQNAGHEASPYQGALVGIQCKTSKGEVYESGVGIIDDYGSFKMKLPQYLSFKMNLP
QYLVNDKGELAGECHAKLLTRYSLKPCSTNGDGDMSSKLVLQTKSKESESDVDTIIFGTSSDLSLSLST
C

Amborellales

>AmTr_v1.0_153.4.1
GMVYCQSCELKGTHSLSEAKPIAGAKVGVACKDEKGRVKWYQAHKTDSYGYFYASVIGFAPSMDAL
SSCTAYLLSSPDPECNLLTNINGGFEGSPLRYKGNLKAGKVLLYATGPLAFRPARC
>AmTr_v1.0_019.72.1
GKVLCQDCTQGWNEWVHGAKPIKGSKVAVTCMDEQKRTVYYASDETDEQGEFEIPLKKEYVKGKSL
STVGCLVRLVSSPDPLCNVPTDFGRGHSGVTLEWPSIVYKDLIKHIVGPFYFTSSMC
>AmTr_v1.0_061.7.1
GRVYCDTCRAGFETSATTYIKGSTVRLECKDRATHQLTYSIEGVTDATGTYRLPVSDDRQHEICEVVV
VSSPEAGCGKAMLGRERARVLLTHNNGIVSNTRYANALAFLKDEPLPRC
>AmTr_v1.0_068.122.1

GVIYCKSCNYSANDTLLGATPLQGATARLECNSSKQVVTAEGKTDKNGYFFIEAPRNKVTNYAFHKCK
VFLASSPLATCQKATDLHGGITGSPLRFHQVLDSGYPLAVFSTGPLAYAPTNATVC
>AmTr_v1.0_047.45.1
GTVTCDACRDTGKSPFVSGATVAVRCQTNGGKRSRHFRVRGKTDEYGDFMIDIPSHMHATPRLENT
CHVTVVQIPENSDCRRNRAGRQAFRLRVSSVGNGIRVYTAGFLHFQPRGAAAIC
>AmTr_v1.0_062.88.1
GRVMCDVCADSSIGPEDHILEGAEVAVLCITKSGEVLNYQAFTNSKGVYSVAETMPESNRWDSCLAR
PISSFHHHCTQLSNSNRAIHFSYSRPSGYSHSLKPFLYKPNTTPLYC
>AmTr_v1.0_00047.2
GTVFCDTCFNGDFSESSHFIQGASVAVECRDRTVKKTFTKEVKTNQHGQFEVKLPFSVSHHIKRIKRC
SVKLVSSSEPYCGVASMAESSSLHLKSKRAGLHIYSAGFFTFKPLKQPDLC
>AmTr_v1.0_041.161.2
GTVFCDACKDGQRSSFDYPLSGAKVAVACAGNDGQVTVWKEESTNWFGNYGMRFEGSPDLSRCFA
QVVANPQGSNCGITGGPARGLNLVFDMFGMEMYVVDSLLSQPSQPMAFC
>AmTr_v1.0_041.169.2
GVGECLDCAKKSFPTDHAFSGLHVSIDCKTDGGKFHSKAAGDLDKNGKFLVKLPSDLLNEEGDLTEE
CFAQLHSGPSSPCPSQAGLEAQKIMLKSKEDGKHEFGTKGKLSFSRSTC
>AmTr_v1.0_066.9.4
GKVYCYRCYDWRYPNKSHDKKHLKGAIVEVSCDADGKKVMVYGVTKNNGKFRVNVEGFDYEKWGG
AKACKAKLYKAPKGSLCSIPTDLHDGKKGASLKVKSKSHEEIVLKAKPFAYAPKTPYHEC

Alismatales

>Zosma4g00040.1
GRVVCDVCGDSSIGPEDHFLEGAEVAVLCITKSGEVVNYQAFTNSKGIYSVAETMSATDRWDSCLAR
PISSFHQHCTRRGNSWSAVRFSYDQTSGHSHIVKPFIYKPTSSPTYC
>Zosma241g00180.1
GRVFCDNCRAGFETAATKYLKGANVKVECRERTTAKLTYSAQGVSDETGTYNIPVSNDHQNDICEALI
MESPENECSQVIKGRERAQVVLTHNDGIPSIKRFANNMGFQTYTPMSGC
>Zosma114g00880.1
GIVYCQRCSAVGTSSLLNARPLLSAKVAITCRDYKNRVRVYLVAPVDFTGYFYTQLDGNDMTSDGIGN
PEESCKVQLVSSGDMTCNRLTNVNKGITGTDIKFEKTLEKGTSFEINLYTASSLAFSPSHC
>Zosma78g00260.1
GKVVCTDCSKDWNEWVNGAKPLQGSKVAVICMDNNDRVVYHTMNITNEEGMFKLIVDRHINGKKLM
VNGCVVKLVSSTHSDCNVLTDFSGGKTGVKLRSPSLVYQDITEYTIPPFSFTSPMC
>Zosma79g00580.1
GKVYCDTCRSGFETSVTNYIEGAKVKLQCKNATTHVLILTMDGTTDASGTYTMDIKDDYSDEVCSVEL
VSSPQSDCKEMKKDRNSVQVMITENSGMSSNVRYANSLGFLRTQAMPIC
>Zosma97g00610.1
GKVFCDTCRAGFETPASTYISGATVKVECRDRMSGDLKYTVQGKTDVTGVYNLVVKNDHKNHICESS
LVSSPDSGCAEVVSGRDRSRIVLTYENGIPSSTRLANSMGFQMQTALAGC
>Zosma89g00790.1
GRVYCDNCRAGFETTISEYLSGARVKLDCQNETTHKVIQTIEAVTDSKGSYHIEISHDHEDETCEVSLV
SSPKPDCSEIKSSRNSARVVLTENSGMASDTRNANSLGFLKKEPLKIC
>Zosma175g00430.1
GQVFCQDCSDSWSLTAAKPLPKVRLEVKCHDYSGKEVFNLAMRTNRLGWFYEPLKGLDLRSYHMDT
FPHFCKVRLLHSIHRNCNAVTNVNYGITGAPLRYEKTLFNGTRYEADLYSTGPMAFRPRHC
>Zosma175g00470.1
GRVFCQDCARLESWSMNGATPLGEARVAIACRDHRHRQFYYKVVRTDEWGFFNQPLKGLDVGRYHI
DTPAHACAAHLLHSNSPNCNVATDTNFGNGGSPLRYEITLFNGTKYESDVYSAGPMAFRPKNC
>Zosma175g00410.1
GQVLCQVCERMGSWSLLDAQPLVRAKVGIKCRDYRYGKIYSMSTETNYAGWYYEPIRKIDLKIHHIET
PADSCTVHLISSDDQNCNVITNVNFGLTGASVRFEKTEFVGTREQSDLYSPGILAFRPKNC
>Zosma6g00520.2
GTVSCDTCFGYKINLITGAVIAVECHSTVDDDRVFRKEVKTSIDGVFRFHLKGVGLRVCSAKLLSSTHP
FCSIGETSSNSDSSLRQLKKLGIDGVVHVYSVGFFTFRPINQPNMC
>Zosma166g00640.4
GEVYCYGCYDSEHSPKSHHKKKLEGAVVKVTCMKGSKDVIAYGKTMRNGMYEITVEDYDVDKYGHE
NCKAMLHAPPKGSSCNMATDIHNGNTGAKLHIKSRNHVEVVYKSKKFAYAPKTPYKDC
>Spipo0G0071700.1

GGVFCDTCQAGFETPASTNIAGAKVRVECRDRASGVVKYVAAGSTDGQGKYRIFVAGEHENDICESV
LVSSPDRWCSSPLAGRERARVVLTHHNGVVSDQRIANNLGFRSDSALAGC
>Spipo2G0004200.1
GRVVCDVCGDSSIGPEDHALAGAEVAVLCITKSGEVVNYQAFTDPKGAYTVAETMPEGDRWSSCLV
RPIGGPHPDCSRRADARAGVKFSYTRPSGYSHAVRPFLFKPSLAPAYC
>Spipo3G0081500.1
GRVYCDTCRAGFETTATEYLAGSKVRLECTHQESDELVHTVEGVTDATGTYHLTVADDHEDEICEVV
MVESPRADCSEIKAGRDRARVLLTHNSGLATNVRLANSLGFLKEEPLPSC
>Spipo2G0081100.2
GTVYCDTCFQQEFSRTSHFISGAVVAVECGEEEAAAAAAAKPIFRAESRTDQDGVFAVRLPPRISKHL
RSIQSCSVKLVSSSEPFCAVASSATPSSSSLRLKSAKPGLHIFSAGFFTFKPLEQPKLC
>Spipo16G0020300.2
GIGECTDCASRNINSADALKGLRVAIGCKDGKGKFQVRGTGKLDRNGKFTVRLPSKLLRRDGELRED
CFAQLHGGPRNSPCRLAASKITLKYRTGHKHTFSTAMKLTFSSSETC
>Spipo4G0069000.4
GKVYCYKCYAWGSPIKSHIKKGFQGAVVKVTCKDVVAFAKTKTNGKYSVTIKGYDYQKYGAAQCKAQ
LYAPPVGSICKIATDLNNGKKGAKLHVKSKSHELIVLEAKAFAYAPKKPYEEC
>Spipo0G0133200.4
GKVYCYRCYDWDHSIKSHGKKHLKGAVVKVTCVDGYKEIVAYGKTKSNGKYSISVKGYDYWKYGAK
HCKAELHAAPKGSVCNIPTDLNGGKKGAKLHVKSESYGLVVLKAKPFAYAPKKPYKEC
>Spipo1G0107300.1
GRVFCDTCRAGFETTVTEYLPGAKVRLECTHQESDELVHTVEGTTDATGTYHLKVADDHEDELCEVV
LVESPRADCLEIKPGRDRARILLTQNSGLATNVRLANSLGFLKEEPLPSC
>Spipo1G0107200.1
GRVFCDTCRAGFETTVTEYLPGAKVRLECTHQESDELVHTVEGTTDATGTYHLKVADDHEDELCEVV
LVESPRADCSEIKPGRDRARILLTQNSGLATNVRLANSLGFLKEEPLPSC

Asparagales

>P.equestris_XP_020587648.1
GKVLCQDCSQGWNYWAHAAQPVKGGKVAVTCTDARRRVVYYGSDETDDKGNFDLPVGPATGDAA
KRIRPEGCSVRLLSSPDSACNVLTADFAGGRSGMRLVRPSHVYPGRIKYTVGPFFFTSPMC
>P.equestris_XP_020595204.1
GRVYCDNCRAGFVTNITEYIEGATVRLECKHFQNDAVEHSIEAVSGADGYYTFIVPNDHEEEICYVKLI
KSPRADCAEFVENREQARILLTENSGQANNLRYANALGFLKDVALPVC
>P.equestris_XP_020591182.1
GSLVCEACLSAGSELTVSHVQGAAVAVSCRTEQRRRRRIRYANGTTDEFGEFIIDLPSQLHAIPKLEEA
CVVRILHMPRNSLCRHLSRGLNPAAKRIRLSSVGNSIRVYTTGMMRTRSLGTGKLSHHEC
>P.equestris_XP_020583077.1
GRVYCDTCRAGFETNITEYIPGAKVRVECTHFLTNKVEHSNEGVTDSSGSYKITVSNDHADEICEVVLV
ESPQENCMEIEKGRDRAQVLLANDGGISGNTRHANSLGFLRNEPLPFC
>P.equestris_XP_020598832.1
GRVVCDVCGDSSIGSEDHPLEGAEVAVLCITKSKEVLNYQAFTNSKGIYKVAETMPESDRWDSCLAR
PISSFHQHCTVRVDAYSGVKFSYNQPSGNSHSVKAFLYKPVSAPAYC
>P.equestris_XP_020583201.1
GKVYCQSCEHIGSWSLEGAKSLASAKVGIACKDYKKRIWFYKSFEADKNGYYYAPIPEMDGTEGVRK
KEISGCSIHLLDSPDSACNLLTNINFGIAGAGVGERKKAVKEKGYEVVVYAAPPLAFKPVNC
>P.equestris_XP_020573394.2
GSVSCMDCSPSHDLSGISLAVNCGGQKTQSYALTNINGQFLISVPNPPPPPSDCSVNLLGARTQLCGF
KNSITSKIIAINSSIKSSNSSYALQTPLAFFTSC
>P.equestris_XP_020582329.2
GSVYCDTCSQQEFSTFSHFIPGASVAITCNRIGKDKLSFQEEVKTNNQGAFKIRIPVEVASRTGKEIKS
CSVKLVRSSEPFCSVASPLTSAVPQLKSIRNSVPVFSAGFLTFRPMKQPDVC
>P.equestris_XP_020593305.2
GTVYCNTCFHQNSSRHNQVLSGASVLVDCNNQITKSGFQNFVKTDKDGVFRVQLPSEISKHIKTIKQC
SVKLISSNDPFCAVAAPPTSSSFHLKSTKNKVHVFSSGFFAFRSLNQSELC
>P.equestris_XP_020587277.2
GSVFCDQCKDGSRGLFDYPLSGARVAVSCGDQYREESSNILGTYSVRFNGNPDLGGCLARVVRGPD
NCSVASGPAQSLKFIFRMFDMEAYSVDPLLAQPSKVMDFC
>P.equestris_XP_020594514.2

GSVECVDCGPKSLRNDEIYKGLKVGIKCKTSDPNEYTTKATTNLNKDGTFHVHLPTHLLTTDHCFAQL
HDSTNKPCSHDDSTEHSKLVYKDDGTYFIVDKLKFSSAAC
>P.equestris_XP_020590284.4
GSVYCFNCYDWKQPLDSHIKKPLEDAIVKVTCDAGDKTYVSYGKTKHFGKYSVNVKGFPFWKHRARA
CRVELHAAPKGSKCKIPTNLNKGAPLKVIVKSHELVVLKAETLAFAPKIPYEDC
>P.equestris_XP_020597326.4
GHVYCFKCYNWKHPFDSSHRKLFKGAVVKATCKAGEKEYIGYGKTKAYGKYSINIEGYPYGKYGAEG
CKVKLHAAPKGSICNIPTKINKGSPLKVEHKSDHEIILRAKSLVFAPSPHKYYEEC

Zingiberales

>GSMUA_Achr4T17330.1
GRVYCDTCRAGFETTATEYIAGAKVKLECKNYTTGEIIHTNQATTDATGKYQITVVDDHQEETCQVTLI
SSPRSDCSEISEGRNSALVVVTHNVGITSGVRYANSLGFLKEKPLETC
>GSMUA_Achr3T03120.1
GRVVCDLCGDSSVGPEDHVLQGAEVAVLCITKSGEVFNYQAFTNSKGIYKVAETMPESDRWVSCLAR
PLSSFHEHCTRRGDAHSGIKFSYNLPSGHSHTVKPFLYKPAAAPMYC
>GSMUA_Achr5T23590.1
GRVYCDTCRAGFQTAVSQYIAGAKVKLQCRNYETDAVEHLVEAVTDSTGTYHIEVEDSHEEEICEVSL
LQSPQPGCTEVAQSRNRARILVTGRSGLASDIRYANSLGFLKDEPLQQC
>GSMUA_Achr7T01790.1
GRVYCDTCRAGFETKATEYIEGAKVKLECKNYTTGASTLTAVAVTNNKGTYQIPVSDDHQEESCAVML
VSSPRSDCSEISDGRNHAAVVLTHNVGITSSVRYANSLGFLKDVPLASC
>GSMUA_Achr10T12160.1
GRVYCDKCRAGFETSATDYIEGAKVKLECRQYDTGVVEQTATAVTDASGTYTLEVEDNHEEEICEVVL
VESPRPGCSEIMTGRDRARVLISAESGLTTSVRYANSLGFLNAAPLPEC
>GSMUA_Achr6T03130.1
GRVYCDTCRAGFETVVSQYIPGAKVKLQCRHYETDSIEHTAVGVTDGTGTYNIEVEDNHEEEICEVSL
VQSPVPGCSEVSGTRNRARVLVTGRNGLASDVRYANSLGFLKDEPLKEC
>GSMUA_Achr5T11390.2
GATECLDCERKSIKHENAVKGLRVAIKCRDGNEKYEIKATGDLDSNGSFNVKLPTELLQDNGELKHEC
LAQLHSASNAPCPDKNGLNPFSKLILKSREKGKHTFTAAAGKLSFSSATC
>GSMUA_Achr3T25240.2
GTVFCDTCFRQELAESSYFISGASVAVECGDAANQLGYRKVATTDRRGVFGVRLPPRISRNLGLVEA
CSVKLLRSNEPFCAVAASATTAGLRLQSRRNGVRVYSAGFFSFKLLNQPELC
>GSMUA_Achr3T15900.2
GTVYCDTCFHRELSKPTHFISVECGDGANKFSYRTVATTNQRGVFRVPLPPRISKHLHLIQACSVKLM
KSEEPFCAVASSATTAGLRLKSWSHGVHVYSAGFFKFKPLDQPELC
>GSMUA_Achr8T28070.2
GTVYCDTCFHQEFSKFSHLISGASVAVECGDAASGRGYRKVVTTNRRGVFGVRLPPRISKHVHLIEAC
SVKLLESNEPFCAVASTATAAGLRLKSRRRGVHVYSVGFFSFKPLNEPELC
>GSMUA_AchrUnT21190.2
GATECLDCAQNSIKTEDAVKGLRVAIKCKVRNEKYDTKAAAELDSNGDFNVKLPSELLQDNGELKHEC
FAQIHSKSDAPCPDMNGLNPSKLVLKSKEGGKHTFVAAPGKLSFSSVTC
>GSMUA_Achr2T20120.1
GRVVCDVCGDASVGPEDHVLQGAEVAVLCITKSGDVLNYQAFTNLKGIYKVAETMPESDRWVSCLAR
PVSSFHEHCTQRGDAHSGIKFSYNRPSGHSHTVKPFLYKPASTPLYC

Poales

>Oropetium_22028A.1
GTVLCQDCTKNWNAYAYNAKPIPGSVVGITCLEQRTRRTVYHGRDATDEKGVFNIEVPAEVSGGGKL
DPSDCLVRLASSGDAGCAVLTNFNGGKTGEKPYRPVKTFPGEVTYAVGPYYTTLPKC
>Oropetium_17805A.1
GEVYCDPCRAGFKTNVSTPLAGAAVKLECRPFLNGPESLKADATTDAFGWYKLEVDQDFQEDICEVV
LVKSPDAECAEVDMFRDRARIPLTKNNGLKQHGVRYGNPIAFLRKVPLQEC
>Oropetium_03834A.1
GSVYCDTCRAGFVTNVTTPIAGARVRLECRHFMSASGAVERSAEGATDAAGKYKIELVDNRGAEEVC
VVALVTSPVAGCAEKEAGRDRAPVALIQDAGLATAVRRANPLGFLKATPLPVC
>Oropetium_11363A.2

GFVACLDCAAGHDLSGVIVAVKCAAGDDGTALLHAAETDAHGAFDVPIPASSGSPCAARVLGATQRL
CAPPGLTVARVVVPERAPADSSSSYQLQLGSRLALFTRC
>Oropetium_25700A.2
GMVFCDQCKDGTRGLFDYPLYGARVAIQCGGGDTPLTVRESNTNWFGGFSIRMEGSPDMNRCTAR
VVQGTGHCGSSGAARELTLAFRMLGLALYTVPPLLSQPEESMDFC
>Oropetium_25797A.2
GLAKCVDCTRKNVMAEAVFKGLNVAIKCKNVNGEYESKAVGKLNGSGAFSVPLAAADLHGADCHAQL
HSAATGKPCPGQEPSTIVPQSDEGTFVAVPGKTTTHYGQEQQSPEC
>Oropetium_23782A.2
GLAKCADCTRKNMKAEAVFKARAGLEVAIKCKNVKNGEYESKATGKLDGSGAFSVPLSADLHGADCH
AQLHSTAAGKPCPGQEPSAIVPQADGTFVAVPGKMTPYQQAEAPAEC
>Oropetium_12372A.3
GVVYCKSCKGRGYNDHMDASPLEGATAMMVCYGRKVVNATGTVTDANGYFVIFFYDLKNFNVRTCK
MYMVSSPSPKCNKPVYPPSQWIGLSLVKEGRTIPPVGFQGLYRPTSVLYYGPAVKGQC
>Oropetium_03650A.3
GMVWCQSCRYKGYFKPMDASPLPGAAVYLQCRHGRRRGATFRGVTGPSGYFLIQTSRQVAAFTSQ
ECRVYVPRSPVRACSVPAYPTGNRGLPLKFQEFVKRGNGLQGLYSVGNRLFRPESPKRC
>Oropetium_12371A.3
GVVWCKGCRYRGYNKSLDASPLPNAAARLRCRRGEWQLSLWNTTGADGRFMIQTDREVTPYTSSD
CKVYVPRSPALGCPVPVKPARKKGAPIKFRRFVPLSDKLQARYSAGNFTFAPEDAAKC
>Oropetium_03649A.3
GVIYCKSCKLRGYNRNMDASPLRNATASLVCYGGEASKYRVLNLTSTATSKEGYFMVMVYDVDMFD
VRSCRVYLRSSPTPLCAAPFVPANVKLGLTLVKEPAGRAPLPKGARGAYHPETALMYKPAAGGKC
>Oropetium_07184A.3
GLVYCQSCAGRGSGSLDGAAPLPGARVTVTCRDRKNRVMAWRKATADENGYFLAKFGVERLRDFM
GDPRKACFARLLSSPNAKCNEVTNVNGGMVGAPLRDEGKRWTGSEGYENVAYAAGPLAFRPSKC
>Oropetium_22738A.1
GEVYCDPCRAGFKTNVSTPVAGAAVKLECRPFLNGPESLEADATTDANGWYRLEVDQDFQEDICEV
MLIKSPDPDCAEVDEFRDRARVPLTRNNGIKQNGVRYGNPIAFLRKVPRNDC
>LOC_Os02g21280.1
GHVVCDVCGDAAIGPEDHVLEGAEVAVLCITRSGEVINYQAFTNSKGVYIVAETMPESDRWESCLARP
ISSFHQHCTKRGDTHSGVKFTYSKPSGNSHTVKTFLYKPANAPLYC
>LOC_Os06g36240.1
GRVYCDPCRAGFETNVSKSIPGATVSVECRHYGAGRESLKAEATTDEKGWYKVEIDQDHQEEICEVV
LDKSSDPACSETEKTRDRSRVPLTSNNGLKQNGIRYANPIAFFRKEPLADC
>LOC_Os09g39950.1
GDVYCDTCRAGFQTNATTAIQGARVRLECRHFMSASGAVERSAEGTTDAAGHYRIELVDNRGAEEV
CAVALLSSPDPECHETEVGRDRAPVTLVQDAGLATMVRRANPLGFLKTRPLPIC
>LOC_Os03g14130.2
GLAMCSGCTRKNMNAEAAFKGLQVAVKCKNSRGEYDKMAVGKVDKSGAFSVPLAADLVGEDGVLK
QDCFARLHSASSAPCPGQEPSMIVAAQQPGHDGAKTFVALAGKVHRPSAEC
>LOC_Os01g67390.4
GKVYCYRCFNEAHPEESHGKEHLKGAMVKVTCQANDQALVGFGYTQDNGKYSVSITGLPLSSTYGA
DSCKVELHSAPGGSDCNVPIELNLSGLSVYSKSNEEVVLQANQVMAFASQKTFGFC
>LOC_Os02g33170.2
GYVACLDCAPGHDLSGVVVAVRCGGGDGGVGQLRAAQTDERGGFDVAVPAAGGDDVDSWRSHPR
CAARVLGGAEQLCAPGGLAVAPVVAAGGREKHGSYALASSLAVFTRC
>LOC_Os03g14140.2
GSIKCLDCSPDDVKAEDAFRGLQVGIMCNSGAGEAYETKMLSGLDENGGFSIPLAADLLRDDGELDK
DCFAQLHSAPETPCAGQTPPRIAKAGPGNDTIAAAAADAAPTYLAVSDDTLFSPVAC
>LOC_Os10g05750.2
GLAKCADCTRKNMKAEAVFKGVRVAIKCKNSNGEYETKATGEVGKSGAFAVPLAADLLGDDGELRQ
QCFAQLHSAASNQPCPGQEPSWIVNAAADKKKTFVAVAGDTHFPSSEC
>LOC_Os10g05790.2
GAAKCAGCGRKNMDAETAFKGLKVAIKCKNGSSEEYESKAVGELDGAGAFAVPLAADLRGADCVAQ
LHSAATDAPCPGQEPSKIEPLSSEGETGTFVAVAGKTHLPSSTSSSPEC
>LOC_Os10g05820.2
GQAKCGECTRKNMKAQDAFKGLQVAIKCKNSDGVYESKAIGDLDGDGAFSVPLAADDLHGAADCFA
QLHSAASSTPCPGQEPSKIVPLSSTTDNGVDKANTFVAVAGKRMYSSTSPAEC
>LOC_Os10g05950.2

GLAKCGDCTRKNMKAKAAFKGLRVAIKCKNGADGEYETKAAGKLDGAGAFRVPLAADLRGADCVAQ
LHSAAHNNAACPGQEPSRVMQLSERTFVAVAGKTHYVSPVC
>LOC_Os10g05970.2
GTAKCADCTRKNMKAEDAFKNLQVAIKCKNGNGEYESKATGKLDGTGAFSVPLDADLHSSDCIAQLH
SATNEPCPGQEPSKIVPMSEGTFAAVAGKTHYRSALC
>LOC_Os01g52650.3
GAVWCKTCRYAGYVKSKDASPLPNAAALLRCRRGKWALSVWGATDARGYFLIQTGTQVAAFTSKDC
RVYYVPRSPSRAACGVALQPGRKTGSPLKFRRFVALPDGLQGRYSAGNFVFGPRDPKKC
>LOC_Os05g45460.3
GTIYCKSCNLSGYNRYMDASPLPNATAQLVCYGDKVLNMTSTATDKNGYFLVMVYRLDVFRRSRCRV
YLGSSPSPLCAAPFIPSNKWLGLTLERERVASLPKGVRGVYRPKSTLMFGPGTGGKC
>LOC_Os05g45480.3
GMVWCKTCRYPGYLAAMDASPLAGAVAYLRCRHGHRRVASIRGVAGSGGYFRIETSQLTSFTSQEC
RVYYVPRSPSRACAVPGHGRRGLPLKFEEFVKRDNGLQGLYSVGNFVFSPKYPNKC
>LOC_Os01g07060.3
GKVYCQSCEHRNSWSLDGARPLRGAEVSVTCRDAKNRAAWWRLAVADESGYFLAEFGVTRASDFL
GADPRGACYARLLSSPDRKCDGLTNINAGMVGAPLRDEGKRWPGQGYDNVVYAAGPLAFRPANC
>LOC_Os10g05910.2
GLAKCGGCSRKNIKAQDAFKGLQVAIKCKNSDGEYESKAVGDLDGDGAFSVPLAADDLHGAAGCFA
QLHSAASSAPCPGQEPSKIVPLPSTTDNGGNKANTFVAVAGKRMHYSSSAECTSAFLC
>LOC_Os10g05860.2
GQAKCGECTRKNMKAQDAFKGLQVAIKCKNSDGVYESKAIGDLDGDGAFSVPLAADDLHGAADCFA
QLHSAASSTPCPGQEPSKIVPLSSTTDNGVDKANTFVAVAGKRMYSSTSPAEC
>LOC_Os10g05880.2
GQAKCGECTRKNMMAQDAFKGLQVAIKCKNSDGEYESKAVGDLDGDGAFSVPLAADDLHGAADCF
AQLHSATSSTPCPGQEPSKIVPLSSTTDNGGDKANTFVVVAGKRMHSSTSPAEC
>LOC_Os10g06000.2
GTAKCADCTRKNMKAEDAFKNLQVAIKCKNGNGEYESKATGKLDGTGVFSIPLDADLHSSDCVAQLH
GATNEPCPGQEPSKIVPMSEGTFIAVAGQTHYPSALC
>LOC_Os10g05930.2
GLAKCGGCSRKNMKAQDAFKGLQVAIKCRNGDGEYESKAVGDLDGDGAFSVPLAADDLHGAADCFA
QLHSAESSTPCPGQEPSKIVPLSSTTDNGGDKANTFVAVAGKRMRYSSSAEC
>LOC_Os10g05990.2
GTAKCADCTRKNMKAEDAFKNLQVAIKCKNTNGEYESKAAGKLDGTGAFSVPLDADLDSSDCIAQLH
SANNEPCPGQEPSKIVPMSEGTFVAIAGKTHYPSALC
>LOC_Os10g05980.2
GTAKCADCTRKNMKAEDAFKNLQVAIKCKNGNGEYESKAAGKLDGSGAFSVPLDTDLHSSDCIAQLH
SATNEPCPGQEPSKIVPLSEGTFVTVAGKTSYPSALC
>Bradi2g07610.1
GRVYCDPCRAGFETNVSKNIGGATVAVDCRPFNGGDSKLKAEATTDQYGWYKIDIDQDHQEEICEVL
LARSPDPACSEIEEFRDRARVPLTRNNGLKQQGTRYANPIAFFRKEPLKDC
>Bradi5g08410.1
GRVYCDTCRAGFETNVTTYIKGAKVRLECKRFGTEKVERALDGVTDETGTYKIELKDSHPEDICEVVLI
QSPLADCNKIQALRDRARVELTRNIGISDNLRLANSLGYLKDVPLPVC
>Bradi3g10980.1
GRVVCDVCADSAIGPEDHVLEGAEVAVLCITKSGEVINYQAFTNSKGIYSVAETMPESDRWESCLARP
ISSFHHHCTRRGDAHSGVKFTYNKLSGNSHNVKTFLYKPVNVPLYC
>Bradi3g15970.1
GQILCVLCLRPGSDLLTFQLPGSKVAVTCKSEGPNTSTMAANSAFATTDESGNFTIELPSRLHATPNLE
DACSVTVLTLPPDSACHAGHSPGSPYRLQPSSSEEDGVRTYTTGSIWLQHNDTPSEEC
>Bradi3g21000.2
GLAKCADCTRNNMKAEQAFKGLHVAIKCKNSNDGEYESKAVGTLDGAGAFSVPLASDLHGADCLAQL
HSTANGPCPGQEPSKIVPLSKAGTFGVVAGKTHYPSVVC
>Bradi1g63370.2
GLAKCADCGRKNLKAEDAFKGLQVAIKCKNDDNGEYESKAVGELDSTGAFSIPLDTDLHSSDCFAQL
HSEENTPCPGQAPSKIMPLSKGDHDGDVHEKNTFVAVAGKTHYSLSTAC
>Bradi3g45020.2
GSVTCLDCAAGHHLSGVVVAVKCTGGAGLHAAQTDGSGSFDVAVPAASGSRCAARVLGGAEQLCA
PQGLAVARVVAAAGGSYALGSRLAVFTRC
>Bradi5g04630.2

GLAKCADCTRKNMKAEEAFKGLQVAIKCKNSKGEYESKAVGGLDGTGAFSVPLASDNAAADCVAQL
HSAASNAPCPGQEPSKVVPVSKGTFGIVTGAATAKTTTASDAAPEC
>Bradi1g68400.2
GSVRCLDCSPNDVNAEDAFKELQVAIKCKSGAGETYETQSLTHLDGTGAFKIPLAAGLVREDGELDRD
CFAQLHSAPDTPCAGAAPPKIAPAGGLNSEDTANTYLAVTDDTILSPVAC
>Bradi5g09480.2
GTVACLDCAQQRNLSGVVVAVKCANGTGVRAAETDGQGRFEVAVPASRSKPGSPCAARILGGPEQL
CAPPRFAASRVVVAHARPGGGSYALTSPLGVFTQC
>Bradi1g68390.2
GLARCSECTRKNMNAEAAFKGLQVAVKCKNSKGEYESKAVGQVDKSGAFSVPLAADLVGEGGELKL
DCFAQLRSASSAPCPGQEPSRIISAQPPSHDGKMTFVALAGKVHRPSAEC
>Bradi3g21010.2
GMAQCVHCARKSMDAEATFKGLGVRIKCKNGNGEYESKAMGKLDSSGAFAVPLPADIDLRAAECFA
QLHSASSSEPCPGQEPSKIVPLSEPDGTFVAMAGKTTELQSHSHHPGSEC
>Bradi3g21030.2
GLAKCADCTRKNMKAEAAFKGLKVAVKCKNAAGEYETKTVGDVDKSGAFSLPLGAELLHEDGELKQE
CFAQLHSAPGHPCPGQEPSQIVRPSSTDAVDKKTFVAVAGKMHYSSKEC
>Bradi2g18920.3
GVIYCKSCKLANYNSGMDASPLPNATAKLVCEEMGGSGRRALEMTSTATDGNGFFLFMVYDVAAFT
RNSGSCKVYLRSSPTMLCDAPFLPEDPTIGMSLAKEAEEEAEHDIYSIQSGALMYKPRSGVSC
>Bradi2g18910.3
GMAWCKTCHYRGYYAPMDASPLPGAVAYLRCVRGRRGVSVRGVAGKGGYFLIQSAKMASFTSSDC
RVVVRASPKRACGAAEFPAADLEGLPLKFERFLTLGDGIQALYSVGNFLFRPNSPGKC
>Bradi2g48670.3
GAVWCQGCRYAGYDKSKNASPLPNAAAQLLCRRGKKWALFASGSTDEGGNFRILTPKQVAPFTSKD
CTVYVQRSPVGACGVALKPSGGKAGSPLKFRKFVPLSDDDLQAIYSAGEFLFGTGPSGKC
>Bradi2g57740.4
GKVYCYRCFNEAHPEESHGKEHLQGAMIKVTCQANDQALVRFGYTESNGKYSVGITDLPLSSAYGAD
SCKVELHAAPGGSDCNVPMELNLSGVNVYSKSNKEVVFQANQVMAFGSKKTFAGC
>Bradi3g20970.2
GLAKCADCTRKNMKAEQAFKGLHVAIKCKNSKGEYESKAVGHLDGTGAFSVPLAADLHGADCLAQLH
SAANNAPCPGQEPSKIVPLSEDTFGVVGGKTQYPSAECASATLC
>Bradi3g20980.2
GLAKCADCTRKNMKAEQAFKGVHVAIKCKNSNGDYESKAVGHLDGTGAFSVPLAADLHGADCLAQL
HSTANNAPCPGQEPSKIVPLSEGTFGFVAGKTHYPSAVCASATLC
>Sobic_001G156300.1
GQLTCELCLRPGSQLLALDMPGAKVAVTCKSDRTPSNQLDSFAFATTDEYGNFTIDLPPQLHATPDLE
KACTVKVLQLPADSCRLRHRTGDTYGLRLSSVEDGVRAYTAGVIRLQDSDTPSDHC
>Sobic_001G308600.1
GKVQCQDCTKNWNAYAYNARPIPRSKVSITCRDKNSRVVYHGSDDTDVQGVFNIEVPKTANVEASRC
LVRLASSGDAACAVFTDFNRGRTGQVPSRLTRASPTKETYAVGPYYC
>Sobic_003G282100.1
GVVWCKSCRYPGYVESRDASPLRNASVLLRCRHGHRALSVWNTTNAQGYFLIQTGTQAAPFTSNNC
KVYVPRSPARGCRVAVSPRRKKGSPLKFRRFVTLTDGLQARYSAGRFTFAPQDPSKC
>Sobic_007G096700.1
GRVVCDVCGDAAIGPEDHALEGAEVAVLCITKSGEVINYQAFTNSKGMYTVAETMPESDRWDSCLAR
PISSFHLHCTRRGDAHSGVKFTYNKPSGNSHTVKTFLYKPATAPLYC
>Sobic_002G140500.1
GSVYCDTCRAGFETNATTPIAGAKVRLECRHYMSASGAVERSAEGATDAAGRYRIELVDNRGAEEVC
SVVLLSSPVPGCAEKEVGRDRAQVELVTDAGAGLATTVRRANPLGFLKSQPLPNC
>Sobic_003G009600.1
GRVYCDPCRAGFETNVSKSVAGATVEVVCRHFEASKETLKAEATTDDFGWYKLEIDQDHQEEICEVV
LKKSPDPACAEIEEQRARARVPLTSNNGIKQKGTRYANPIAFFRKDPLKEC
>Sobic_006G059100.1
GRVYCDTCRAGFVTNVTEYMAGAKVRLECKHFGTGEVERAIDGVTDATGTYTIELKDSHEEDICQVVL
VQSPRKDCDQTQPLRDRAGVLLTRNVGIADSLRPANPLGYFKDVPLPVC
>Sobic_003G282200.1
GMVWCKSCRYDAPSMDASASPLPNADALLQCQRGDDGRAVSVWNTTDAGGHFLILADWQSAAFKS
KDCRVKVYVPRSPAAGACTVAVKPNPKGAPLKFRRFVPLSGEMQARYSAGDFTFAPEDPAKC
>Sobic_001G265900.2

GLAKCADCTRKNMKAEAAFKGLEVAIKCRNSKGEYESKAIGKLDVSGAFSIPLSTDMHAADCVAQLHS
AAGTPCPGQEPSRIVPQSSDGNFVVVPGKTDYPSAEC
>Sobic_001G266100.2
GLAKCADCTRKNVNAEAAFNGLKVAIKCKNSKGEYESKAVGELQSTGAFSVPVSVNLAGCVAQLHNA
AGTPCPGQEPSRITPLSDGTFVAIPAGNTQQASSAVC
>Sobic_001G438500.2
GLAKCSDCARRNMNAEAAFKGLQVAVKCKNSKGEYESTAVGQVDKSGAFSVPLAADAVGEDGELKS
ECFAQLHSASSAPCPGQEPSKIVAAPPGGHDGNEKTFVALGGKVHRSSPEC
>Sobic_004G167000.2
GSVACLDCAAGHDLSGVVVAVKCAGNGDGGGAGMHAAQTDGGGNFDVAVPGASASSASQPCAAR
VLGATEQLCAPQRLTVARVVPARARAPGSAAAASYVLGSRLAFFTRC
>Sobic_001G266300.2
GLAKCADCSRKNMKPEAVFNGLQVAVKCKNADGVFVTKALGEVDRTGAFSVPLAADLLREDGELKQ
DCFAQLHSAANQPCPGQEPSWIVRSATSEYDDDKTKKTFVAVAGKVHYPSKEC
>Sobic_001G438400.2
GCVKCLDCSPNDVNAFKDLQVAIKCKSGAADDESYEMRALGTLDDTGVFRIPLAAELLRDDGSMDRD
CFAQLHSSLGTPCVGQAPPRIAPTTSQSDGGSATSDTTYLAAAADTVLAPVAC
>Sobic_003G390500.2
GKVYCYRCFNEAHPDESHGKKHLEGAMVKVTCQANDQALVAFGYTKSNGKYSVILKGLPISNNYGAD
SCKVELHGAPGGSDCNVPIELNLSGLSVYSKSSEEVVFKANQIMAFASKNTFGC
>Sobic_004G001700.2
GMVFCDQCKDGARGLFDYPLYGARVAIQCGGGDTPLTVRESNTNWFGGFSVRMEGSPDMNRCTAR
VVQGTGHCGAGAGAGGGGAPRELTLAFRMLGLALYTVPPLLAQPEEAMDFC
>Sobic_001G266200.2
GLARCADCTRKNMKSEAAFKGLQVAIKCKNTNGEYETKAVSELQSTGAFSVPLAADLHGAECHAQLH
SAANAPCPGQEPSRIAPMSGGTFVAIPGKTHYNPSAEC
>Sobic_003G282300.3
GVIYCKSCKGKGYNTGIDASPLQGATAMMICYGRKVVNATGTVTDANGYFLIMFYDVKNFNARTCKM
YLLSSPTPQCSKPYYPPNQWIGLSLVRETRTIPPAGLQGIYTPTSVLFYAPGAKGQC
>Sobic_009G208600.3
GLIYCKSCKLKGYNSGMDASPLPNATASLVCYGDEDSKFRVLNQTSTATDKNGYFLVMVYDVDMFDR
HSCRLYLRSSPTPLCAAPFVPSNPKLGLTLVRDRKATAPRGARGIFHPKTALMYAPATAGKC
>Sobic_009G208700.3
GVVWCKSCRYHGYFAPMDASPLPGAKVYLRCKHGRRAVTVAGQSGASGYFLIQTSQQVSAFTSQQ
CRVYVPRSPVRACGVPAYPSGRKGLPLKFQEFVKRGNGLQGMYSVGNRFFRPKYRGKC
>Zm00008a001995.1
GKVQCQDCTKNWNAYAYNAKPIPGSKVSITCRDDRSRVVYHGSDYTDGQGIFNIEVPSKATNGCDLA
PSRCLVRLASSGDAACAVFTDFNRGQTGQVPARLTHASPTKVTYAVGPYYCTLRQCDDVKGDDDDA
C
>Zm00008a037818.1
GRVYCDTCRAGFVTNVTEYIAGAKVRLECRHFGTGKLERAIDGVTDATGTYTIELKDSHEEDICQVVLV
ASPRKDCDEVQALRDRAGVLLTRNVGISDSLRPANPLGYFKDVPLPVC
>Zm00008a007539.1
GRVYCDTCRAGFVTNVTEYIAGAKVRLECKHFGTGKLERSIDGVTDANGTYTIELKDSHEEDICEVVLV
ESPRKDCDQVQADRDRAGVLLTRNVGISDNLRPANPLGYLKDVPLPIC
>Zm00008a002529.1
GRVWCDNCRAGFETPASTYIAGAKVRVDCKSKTTGANTCSFEGHTDRTGTYNILVIDEHEHELCESVL
VSSPDKGCANVVAGRERAPVFLTSNNGVASNVRLANALGFQKDVALPRC
>Zm00008a016045.1
GRVVCDVCGDAAIGPEDHALEGAEVAVLCITKSGEVINYQAFTNSKGVYTVAETMPESDRWDSCLAR
PMSSFHLHCTRRGDAHSGVKFTYNKSSGNPHTVKPFLYKPATVPLYC
>Zm00008a027487.1
GSVYCDTCRAGFETNATTPIAGAKVRLECRHYMSASGAVERSAEGTTDAAGKYRIELVDNRGAEEVC
SVALVSSPVPGCAEKEVGRDRAQVSLFTDAGAGLATTVRRANPLGFLKDKPLANC
>Zm00008a002384.2
GLAKCADCTRKNMKADAAFNGLQVAVKCKNADGVFETKALGEVDKSGSFSVPLPADLLREDGELKQ
DCFAQLHSAANKPCPGQEPSWIVRPTTDNDDDDKMKNTFVAVAGKVHYPSKEC

>Zm00008a035700.2
GSVKCLDCSPSDVDAFKDLQVAIKCKSGADEGYETKARGPLDDTGVFRIPLAAELLRDDGSTDRDCF
AQLRSSPDDTPCVGQAPPRMIAPASQVDGSSSTNTTYLAAAAAADTALSPVAC
>Zm00008a002390.2
GLAKCADCTRKNLKAEAAFKGLEVAIKCKNSKGEYESKAMGKLDGFGAFSIPLSTDLHGADCVAQLHS
AAGNPCPGQEPSRIVPQSSKGNFVVVPGKTDYPSKEC
>Zm00008a035701.2
GLAKCSDCARRNMNAEAAFKGLQVAVKCKNSKGEYESTAVGQVDRSGAFRVPLAAADVVGEDGELK
SVCFAQLHHSASSAPCPGQEPSKIVAAPPGHDANGEKTYVALGGRVYRSSPEC
>Zm00008a021942.2
GSVACLDCDEEHDLSGVVVAVKCAGDDDGGAGLHAAQTDGRGNFDVAVPAAASASGAPCAARVLG
ATEQLCAPWGLTVARVVPARAPGSASYPASYVLGSRLAFFTRC
>Zm00008a000921.2
GLAKCSDCARRNMNTEAAFRGLQVAIKCKNSKGAYESAAVGQVNKSGAFSVPLAADAVGEDGELKS
ECFAQLIHSASSAPCPGQEPSRIVAAPPGHTAHTNGNGKTFLALGGRVYQPSPEC
>Zm00008a018944.2
GMVFCDQCKDGARGLFDYPLYGARVAIQCGGGDTPLTVRESNTNWFGGFSVRMEGSPDMNRCTAR
VVQGTGHCGAASSSGGGAVPRELTLAFRMLGLSLYTVPPLLSQPEEAMDFC
>Zm00008a013040.2
GKVYCYRCFDEAHPDESHGKKHLQGAMVKVTCQADDQALVAFGYTRSNGKYSVILKGLPISSSSNYG
AADSCKVELHGAPGGSDCNVPIELNLSGLSVYSRSSEEVVFKANQIMAFASKNAFGC
>Zm00008a013934.3
GVIYCKSCKGKGYNTGIDASPLQGATAMMVCYGRKVVNATGTVTDANGYFLIMFYDMASFSSKTCKM
YLVSSPTPQCSKPFYPPNQWIGLSLVRETRTVPPAGLQGIYTPTSVLFYAPAAKGQCPY
>Zm00008a013935.3
GTVWCKSCRYAGYVQSSMDASPLPDAAALLQCRRGDGRAVSVWNTTGADGHFLIQADWQSAPFNS
KDCRVKAYAPRSSPPAARACTVAVNPSPNKGAPLKFRRFVPLSGEMQALYSAGDFTFAPEDPASKC
>Zm00008a011025.3
GLVLCQSCAARGSQSLDGAAPLPGAQVLVTCRDRKNHVMAYRRGVADHNGYFHAEFGVEPAADFL
DKDPRGACFARLLSSPDARCNGVTNVHGGMEGAPLRDEGKQWTDARGFRNVVYAAGPLAFRPGKC
A
>Aco018698.1
GVVYCQNCSFRGTWSLESAATVAAARVGVTCLGWKNQVIIRRAASTDAAGYFYAALDAAGDFYKGD
PARACYVRLLASPNAACDDPTNINYGFTGAALKDEGKRAAAKLLDVMIYSAGPLAFQPASC
>Aco003161.1
GVVYCKMCQLPGYVKSLDASPLPGAVAQLRCNNGGRKNAALLIAATTDVRGYFKIQTTKVTSAMARN
CRLFLVSSPFQGCSIPVYADGEGAATGFPLKFETNAAAGGAAAKAIFAAGFFEFAPVDIASC
>Aco002832.1
GRVFCDTCRAGFETPATTYLAGAKVRIECRARSTGIQTYNAEGSTDERGVYRIPVTGEHEHEICESVL
VSSPHSGCAVALKGRERARVHLTRNNGIASETRFANSLGFEKDTPLPAC
>Aco012434.1
GALVCDACFLPGPHLRTSNIRGAKVAVACEIEEKRRKINWAYGTTDEYGEFIVDLPSHLHAIPRLDKDC
AVRILHIPNDSHCRPTSHVNPRRIWLSSVGNSIRVYTTGAVRLSNKQTPANAC
>Aco017800.1
GRVYCDTCRAGFETSATEYIEGAKVRLECKHFATGAVEHAVEGVTDKTGTYKIALTDNHEEEICEVVL
VDSPLAGCSEVTPGRERARVLLTQDSGIATNVRYANPLGFLKEQPLPVC
>Aco016210.1
GRVYCDTCRAGFETSATQYIEGAKVRLECKHFATGVVEHAVEGVTDGTGTYRIELADSHEEEICEVAL
VASPLSGCAEIAAGRDRARVLLAHDSGLASNVRYANPLGFLKEKPLAVC
>Aco003923.2
GAVFCDTCFLRKLSESSRFISGATVAIECRRDESARPSFRKEVLTDRRGVFRIRLPAEVTKHVKSVRPC
SVNLIRSSDPFCAAPSTAALLLHRFKSRWQAADVRVLSAGIFAFKPLQRPEFC
>Aco019419.2
GTVFCDQCKDGQRSIFDYPLSGATVAVECGSGGAVLKEDTTNWLGFYTVRVDGSQDLSACTARVVA
APSSCGAAAGPPRPLALMFRMLGMALYAAEEPLLSEPREPVGFC
>Aco019424.2

GSAVCSDCATKKIEHERAFNGLHAAVKCKAATGLYETKAMGELDNTGSFNIPLPADTLAADADDLNHD
CFVELHSTASPEATTPCPAKPSKLIPAAKANGGERVFTAASGPLSFASATC
>Aco005612.3
GIVFCKRCKFPGYNRDMDASPIPGAIATLRCYNGRRAVSLKARTDNNGYFLIQTSSLTTFTSNKCRVFV
KPSPTTSYCNILVYPRSPGASLKFEKVLKLPFGLEGLYTAGVFVFAPSVPTC

Ranunculales

>Aqcoe4G128000.1
GTVVCEACLARETGVYAYPVSSAMVAVSCKNGRGIMKKNNWVQETTNEYGDFMIDLPSRLHAIPSLH
KACVVKVLRLPKDSPCKRFPIGKAKTIKLLSNRNNLRTYSVGSIRLQQTSKLSRSC
>Aqcoe1G228100.1
GEVYCDTCRVLFPTTVSTPAKNAEVLLECRDRENENNRTLSQSTTDIDGNGRYAIEVDGDHEDEICEV
RLVQSLDTECNQIIPGLDRARVLISDNSGIASNVRYANPLGFIKAVPLPEC
>Aqcoe1G120900.1
GKVLCQDCTGDWNDWVQGSKPIKDSKVSVTCFDDRGRVVYYESDKTDERGEFDMIVQKSINGKELK
PNQCSVRLVSSPDNVCNLLTDFAGGRSGVKLSRPSHVLPDLVKYTLGPFFFTTPMC
>Aqcoe6G083400.1
GQVYCDTCRTAFVTKASNLIEGAKVALNCRDRAAGDLTYSLEGVTDSTGSYKLAVEGDHEEEICEIKLI
SSPREDCNETIPGLDNARVLVTSNNGISELSRFVNSLGFLKKEPLPEC
>Aqcoe2G160700.1
GRVVCDVCGDSTIGPEDHVLEGAEVAILCITKSGEVLNYQAFTNSKGIYTVAETMPESERWDVCLARPI
SSYHNECTHHGDANVGVKFTYKLPSGYSHTIRPFLYHPTNVPMYC
>Aqcoe5G210700.2
GVGECSDCAQNNIENKHAVSGLPVAIDCKAADGTFKTRGVGELDEEGKFKVVLPSEIVKDNGELEEEC
FAQLHSASSSPCATNNGLEALKIVFKSKDNEKITFHPAGKLVFSPNTC
>Aqcoe5G210800.2
GTVECADCAVNNIKTKHSPSGLQISIDCKVADGELKTVGVSQLDEKWQYKVILPSGMVKDGQLKEDCF
AQLHSASSIPCPSNNDLEASKLIFKSNSNGKLTFSPAGKLAFSPITC
>Aqcoe3G376000.2
GTVYCDTCFQQDFSKTSHFISGASVAVKCGNRNLKTSFYKEVKTNQHGVFEVHLPTGVSKHVKKIKG
CSVRLINSNEPFCAVAASAKSSSLNLKARKEGTHIFSAGFFTFKPLKQPEFC
>Aqcoe1G439100.2
GSVSCLDCAKHDDLSDVNVLVKCDKVKQFATTTTEKDGSFETKLNVDPSSTNCQAQLLGGPEQLCSY
KKSLVSKIVKDHDSKSYTISTPLAFFTSC
>Aqcoe5G117200.2
GTVFCDQCKDGHRSLFDYPLSGMKVRVTCDSMEKEETTNWLGSYTVSFEGSPDLSGCYAHLLEDGQ
GSTKCGAVAGPAQQLKLMFKIFGMEMYAVDSLLSQPARPMSIC
>Aqcoe1G153900.3
GMVYCQSCKYYGTWSLTDAKPIPSAKVSVTCKNHKNIVTFYKIFVTDEGGYFYAPLDGYKMDDYVLD
HPLHSCEVRLVASPLPTCNSPTNINYGLNGASLRYENKRIFGSNYEAVIYAAGPLAFRPGYC
>Aqcoe4G097400.3
GVVFCKPCQHKGVDTLHGATPITGAVVRLQCNNTRIPFELQAKTDKNGYFFLHTAPKLSNYGIHKCKA
FIVSSPLNSCRTPTDLHQGVTGAVLRYERPSPKPNQYALFTVGPFAFEPTSC
>Aqcoe1G135900.2
GTVFCDQCKDGHSSLFDYPLSGMKVRVTCDSMEKEETTNWLGSYTVSFEGSPDLSGCYAHLLEDGQ
GSTNCGASQQLKLMFKIFGMEMYAVDSLLSQPARPMSIC
>Aqcoe3G375900.2
GTVYCDTCFQQDFSKTSYFISGASVAVNCANRNLKTSFYEEVKTNEHGVFEVHLPTAVSKHLTSIKGC
SVRLIDSSEPFCAVASSATSSSLHLKARKEGTHIFSAGFFTFKPLKQPEFC
>Aqcoe4G227000.2 / JZ012168
GTVYCDTCFQQDFSKTSHFISGASVAVNCANRNLKTSFYEEVKTNEHGVFEVHLPTAVSKHLKSIKGC
SVRLIDSTEPFCAVASSATSSSLHFKARKEGTHIFSAGFFTFKPLKQPEFC
>Aqcoe4G047900.1
GQVYCDTCRTAFVTKASNLIEGAKVALNCRDRAAGDLTYSLEGVTDSTGSYKLAVEGDHEEEICEIKLI
SSPREDCNETIPGLDNARVLVTSNNGISELSRFVNSLGFLKKEPLPEC

Asterales

>Lsat_1_v5_gn_1_4180.1

GRVYCDTCRAGFETSATTYIPHAKVRVECKNRQQKVLYSMEGMTDATGTYHIRVNEDHGDETCDVVL
VSSPLGNCNKADPGRDRARVVLTSYNGIVSDTRFANAMGFMKDEVMSGC
>Lsat_1_v5_gn_7_109420.1
GRVVCDVCADGSVGPEDHVLEGAEVAVLCITKSGEVLNYQAFTNSKGIYTVAETMPESERWDACLAR
PISSFHEHCTHLGDGFSGVKFGYNHVSGYSHAVRPFVYRHASIPMYC
>Lsat_1_v5_gn_2_124301.1
GKVYCQSCKYFGSWSLTGAEPIPAAKVSVICKNHKKRVSYYNTFATNEEGYFYAELKDFKMTHYLLDH
PLHACHVKLVSSPLATCNLLSNVNNGINGSPLKFENKVLHGKDYEAVVYGSGPLAFRPSNC
>Lsat_1_v5_gn_5_186660.1
GKVYCDPCRIQFPTKISYPIPNAKVVLVCRAREQSGESYNVEGTSDANGMYSITATGDHEEEICDVHV
TESPDSKCPEVMDDERSARVSLTDKNGVRGNSRAANPIGFMVKEVDPRC
>Lsat_1_v5_gn_2_90181.2
GIVYCDTCFNRDFSHSSHFISGASVVVECGGNGVGASGMESQFREEVKTNWKGEFTVKLPYAVTDKI
RECSVKLIRSNQPDCPVVATSTESSITLKAKTPGNRIFSAGIFTFRPRKQPREC
>Lsat_1_v5_gn_4_173421.2
GTVYCDTCFRQKVSKSTHFISGAKVAVECGGDGGKQSFRVEVKTNEKGEFEAKLPVSVGRSVEKIKG
CSVRLISSGQPYCAVAATATSSEIRFKSKKAGTHVFSAGFFTFKPELC
>Lsat_1_v5_gn_8_126000.2
GSVFCDQCKDGQVSLFMDYPLTGVKVAMACPGQGGQLKVISEETTNFLGSYVMRFDGAPDMSGCR
AQVSGDGQGCRAVAGPAQSLNLVFQMFDTSIYTVGHLISQPAQPMPNC
>Lsat_1_v5_gn_3_45680.2
GIGECADCKDINIKSTHALSGLKVTVDCKLENGKLKTKGVGELDGEGNFKISLPQEILKDGKLTEECYA
QLHNAANKPCAVHDGLEATKITFLSKSDQKHTFQPVGKLKFSSAVC
>Lsat_1_v5_gn_7_21560.3
GMVYCKACKYKGIDTLVGATPLQGAVVLLTCNNTKYPLRVKSTTDKNGFFFIMPPKTLTTFGVHKCKV
TLLSSPKATCNTPTNLHYGIKGATLVPTPKPKIVLTPVLPFDVFTVGPFAFEASKKTRC
>Lsat_1_v5_gn_4_53840.3
GVVYCKACKYKGVDTLLGATPLPGAEVLLTCNNTKYPLRVKGTTDKNGYFFIKPTKTLTTYGSHTCRV
SLLTSPMATCNAPTDLHAGVKGSLLLPNKKPPLSSPDAHPLPYDVFSVGPFAFEPSSKTPC
>Lsat_1_v5_gn_8_91860.3
GKIYCDPCRIQFPTKISFPLRDVKVILACHKENSNVETYRVEGMSDENGNYKLKAVGDHAEEICEISVT
ESPEINCPDLMDDENHVRVSLTTKHGIKGRARYANPLGFMAEVADPRC
>Lsat_1_v5_gn_2_51461.4
GKVYCYSCYDWKYPIKSHAKHHLKGAVVEVTCKAAGEKEISVYGKTKINGKYAITVEGLDYSKYGGAK
GCIAKLHMAPNGTKCNIPTNLHGGLKGAELKVKSKNAYEIVLQAKAFAYAPKTPSKIC
>Lsat_1_v5_gn_2_50521.4
GKVYCYSCYDWKYPIQSHAKHHLKGAVVEITCKAIGQKEIFAYGKTKINGKYAITVDGLDYSKYGGAKA
CTAKLHMASTGTKCNIPTNLHGGLKGAVLKVKSKNAYEVVLEAEPFAYAPKTPSTLC
>Lsat_1_v5_gn_2_51640.4
GKVYCYSCYDWKYPIQSHAKHHLKGAVVEITCKAVGQKEIFAFGKTKINGKYAITVEGLDYSKYGGAK
ACTAKLHMAPKGTKCNIPTNLHGGLKGAMLKVKSKNAYEVVLQAEPFAYAPKTPSTLC
>Lsat_1_v5_gn_2_51341.4
GKVYCYSCYDWKYPIQSHAKHHLKGVVVEITCKAVGQKEIFAYGKTKINGKYAITVEGLDYSKYGGAK
ACTAKLHMAPNGTKCNIPTNLHGGLKGAVLMVKSKNAYEVVLEAKPFAYAPKTPSTLC

Vitales

>GSVIVT01006374001.1
GRVYCDTCRAGFETSATTYIAGARVRIECKDRNSLQLVYSVEGVTDSTGTYKFSIADDHGDQMCDAV
LVKSPQPDCAKVDAGRDRSRLSLTRSNGLVSDTRFANAMGFMKDEPASGC
>GSVIVT01008996001.1
GRVYCDTCRAGFETSATTYIAGAKVRVECKDRNSMQLLYSIEGITDSTGTYKIMVTEDREDQLCDAVL
VSSPQSDCASVDPGRDRAAVILTRYNGIVSDNRYANSMGFLKDHPMSEC
>GSVIVT01021211001.1
GQVYCDTCRIQFITRVSEYVPGAKVRLECRDREGGSVTYSIDGETDETGSYRLPVEGEHEEEVCEVQ
LIKSSKPECSEISRGVFARRSARISLASNNGMASSDRLANPLGFLKQESLSEC
>GSVIVT01037612001.1
GRVVCDVCGDSSIGPEDHILEGAEVAVLCITKSGEVLNYQAFTDAKGIYRVAETMPESDRWDACLARP
ISSFHEHCTHLGDGTSGIKFTYSRPSGYSHTIRPFVYRPANAPLYC
>GSVIVT01032773001.1

GMVYCQSCDQFGSWSLTGAEPIPAAKVSVICKDHKDQVSFYKAFETDGNGYFYAELDGFKMSHNILD
HPLHSCHVKLVSSPLENCNLLSNVNYGLYGSPLRYENKRLFGKHYEAVIYAAGPLAFRPAHC
>GSVIVT01036543001.2
GTVFCDTCFQEDFSKTSHFISGASVVVECGDGSLKPSFQKEAKTNEHGVFKVHLPISVSKHVRKIKRC
SVRLISSSEPYCSVASTATSSSLRLKSRKQGIHIFSAGFFSFKPLKQPYLC
>GSVIVT01024076001.2
GVVECADCSESHIKASQAFSGLGVTIDCKLANGEFKTRAVGDVSNEGKFKVSLPEEMVKDGYQLKEE
CFAQLHSASAAPCPDAQSGLASKIVLKAKTIGKKNVFGPIANLKFSSLTC
>GSVIVT01024071001.2
GTVFCDQCKDGQRSLFDYPLYGIKVTMACANSNGQITMWREETTNWFGTYAMRFDGAPDLTGCYA
QISGSGQGSTGCRAAAGPAQSVRLMFRMFDMEMYAVDSLLSQPAQPMSFC
>GSVIVT01024081001.2
GIGECADCKQRNIKTSQAFSGLRVTIDCKLANGEFKTRAVGELDEEGKFKVSLPEEIVKDGELKEECFA
QLHSASATPCPAHNGLESSKLILKTKVDGKHTFGPAGKLKFSPATC
>GSVIVT01007286001.3
GVVYCKACKYRGIDTLLGASPLLGATVKLQCNNTKYPLVVLGKTDKNGYFFIQAPKKITTFGAHKCKVS
LISSSSPTCNLATDLHFGLKGAILRPEKPPAGLPPPPYVTFSVGPFAFEPAKKVEC
>GSVIVT01024076001b.2
GIVECADCSESHIKASQAFSGLGVTIDCKLANGEFKTRAFGVVSNEGKFKVSLPEEMVKDGYQLKEEC
FAQLHSASAAPCSDAQSGLKASKIVLKAKTIGKKNDFGPIENLKFSSLTC

Malpighiales

>Lus10003874.1
GRVVCDVCGDSSMGPEDHVLEGAEVAVLCITKSGEVVNYQAFTNAKGIYTVAETMPESDRWDACLA
RPISSFHEHCSHLGASSKGVKFSYKKPSGYSHSVRSFVYRHSSPPSYC
>Lus10042201.1
GKVYCDTCNFGFETPKSTSIPGATVKIQCRNRKSNELVYEREGETNSEGMYEIHVDEDHMDQVCDAK
VVNSPQLDCFKPSAGRDQARVILTDSNGIVSKVRFANAMGFSKEGTVDGC
>Lus10001895.1
GKVYCDTCRTQFITRLSDFMKDAVVRLECKDREGGSVTFSETAITDEHGVFHIPVTGDHEDEICEINLIK
SPRPDCSEIPKQEHPNDETARISITKHNGMTTGERQANPMGFMVAKPKPEC
>Lus10023887.1
GKVLCQDCQKSYADWVNGERPIKGSKVSLTCMDERKRVIHYDSDTTDDRGQYEMVVSKYINGKLLN
EKLCQVRLVSSPDSNCNVMTDFAGGKSGVKLGQPAYVYRGSTKYEVGSVYFTHPRC
>Lus10039078.1
GSVVCQACSSTTVDHQDLHQLHEWPVPGAVVAVNCRGKKKHRKSTTQARTVTDEYGDFIIDLPSELH
GIADFDKMCSVEVLRMPKDSACRPAYVQNKHKALKLSSFGNGIRNYSAGKIQFLDTTSKPLQSC
>Lus10040948.1
GVVYCQSCKNSGSWSLSGANPLHSATVSVICKNSHHQVSFYKTYQTNEYGYFYAQLDGFKLGHGILD
HPLQACTVKLVSSPLQTCNIPTNLNYGIKGAKLRFENKVLRFPHYEAVIYAAGPLAFRPSEC
>Lus10017440.1
GIVYCQKCQNPGSLTGATPIPYAKVNVNCRNSRHMLTFQKVYTANQHGYFFAQLDGFRLGHGIFDIPP
LQACTAKLVSSPLRSCNVATEINSGPYGATLRSENKVYKYPPYETVIYAAGPLAFRPSNC
>Lus10034365.2
GTVYCDTCFQDAGTRFISGASVAVECRRGRFRQEVKTNEQGKFKVKLPFSVSKQVQSIRRCSVKLVS
SSDPSCAVTSTANSTSSLHRINKQGDDLHVFSAGIFTFKPAQHENSLC
>Lus10026279.2
GSVFCDQCKDGQLSLFDYPMTGVRVTMACVDENGETLMSREERTNMFGWYNIRFDGTPDLRNCYT
QFAPDSSSTGEGSSSGGGSANSCIAAAGPPQKLRLMFNMFGMEMYSVDSLLSQPAKPMSFC
>Lus10015434.3
GVVYCKPCKYSGVDTLLGATPVLGATVKLQCNNSRVPLVFKATTDKNGYFKVKAPKTITNYGVHKCKA
SLVSSPDAKCSFKSTLHGGTTGGSLRVDKKFVESNQTYVMFTVGPFAFEPKC
>Lus10025526.4
GKVYCYRCYDWAYPTKSHDKKHLKGAVVEVTCKAGEKEIKAYGKTKINGKYSITVDGFEYGKYGAKA
CTAKLHAPHKGSKCSIPTKLHGGNKGAELKVKSKDYHLIVLQAKSFAYAPKKPYAEC
>Lus10008615.1
GKVYCDTCNFGFETPKSTSIPGATVKIQCRSRKSNELVYEREGTTNSEGMYEIHVDEDHMDQVCDAK
VVNSPQLDCFKPSAGRDQARVILTDSNGMSSKVRFANAMGYSKEGTVDGC
>Lus10007515.1

GVVYCQKCQNPGSLTGATPIPYAKVNVNCRNLRHMLTFQKVYTANQHGYFFAQLDGFRLGHGIFDIP
PLQACNVKLVSSPLRSCNVATEINSGPYGATLRSENKVYKYPPYETVIYAAGPLAFRPSNC
>Lus10009837.1
GVVYCQRCHNSGSWSLSGANPLHSATVSVICKNSHNQVSFYKTYQTNEYGYFYAQLDGFKLGHGILD
HPLQACTVKLVSSPLQTCNVPTNLNYGIKGAKLRFENKVLRSPHYEAVIYAAGPLAFRPSEC
>Lus10005086.2
GTVYCDTCFHDADTRFISGASVAVECRRGRFRQEVKTNEQGKFKVKLPFSVNKQVQSIRRCSVKLVS
SSDPSCAVASTANSTSSFHRIKNNQGDNLHVFSAGFFTFKPAQHKNSLC
>Lus10001821.2
GRVVCDVCGDSSIGPEDHVLEGAEVAVLCITKSGEVVNYQAFTNAKGIYTVAETMPESDRWDACLAR
PISSFHDHCNHLGASSKGVKFSYKKPSGYSHSVRPFVYRHSSPPSYC
>Lus10042394.2
GSVFCDQCKDGHLSLFDYPMTGVRVTMACVDENGETLMSREERTNMFGWYNIRFDGTPDLRNCYT
QFAPDSSSTGEGSSSSGGSANSCIAAAGPPQKLRLMFNMFGMEMYSVDSLLSQPAKPMSFC
>Potri.011G137100.1
GRVVCDVCGDSSIGPEDHVLEGAEVAVLCITKSGEVLNYQAFTNDKGIYTVAETMSESDRWDACLAR
PISSFHEHCTQLGETSTGVKFSYNRPSGFSHRVKPFVYSPANVPTYC
>Potri.011G111300.1
GKVYCDTCRVEFQTKISDAIPGAKVKLVCNNRENGTLTYTVEGTTDSSGTYRLPVVGDHEEDICEVRL
VESPRADCNEPFKSVDSARILLTKNVGVVDNLRYANALGYMKKVAQPEC
>Potri.002G093100.1
GLVYCDTCLAGFETPKTTYIAGSKVKVECRDRKTQDLVYSKEGTTDSTGKYIITVDEDHKDQICDAMLV
SSPRKDCSSPSAGRDRARVILTSYNGLVSTTRYANSMGFMAAQPMSGC
>Potri.001G392400.1
GKVYCDPCRVEFQTKISEGIPAAKVKLVCNNRDNGTETYTVEGATDNSGTYRLPVAGDHEDDICEVRL
VESSRPDCNEPFRSIDSARILLTKNVGVVDKTRYPNALGYMKKEAQPEC
>Potri.016G006200.1
GTILCEACLNGGTQLLHAWPVSGALVYVECHTGGKWSKTTSSQAMTDEYGDFLIDLPSHLHGIPNLER
TCSVKVLRLPQNSVCRPAHARKQKALELSSVGNGIRNYSAGEIKFLQVTSKPLQAC
>Potri.010G185400.1
GMVYCQSCKYSGSWSLTGAKPIPSAKVSVICKNSNKQVTFYKAFETDAYGYFYANLDGFKMSNIVLD
HPLHGCHAKLVSSPLPNCSLLSNINYGLYGAPLRFKNKVLRGTHYEVVIYAAGPLAFRPAQC
>Potri.006G005600.1
GTVLCEACLHGENQLHAWPISGALVNVECHTSTKRSKKSSAQAITDEYGDFLIDLPSHLHGIPHLERIC
SVKVLRLPQNSVCRPAHARKQKALKLSSVGNGIRNYSAGEIKFLQVTSKPLQAC
>Potri.008G072000.1
GMVYCQSCKYSGSWSLTEAEPIPSAKVSVICKNFKKQVTYYKAYETNAYGYFYAQLDDFKMSNNILD
HPLHGCHVKLISSSLANCSLLSNVNYGLYGAPLRFENKVLRGSHYEAVIYAAGPLAFRPAQC
>Potri.001G392400.1
GKVYCDPCRVEFQTKISEGIPAAKVKLVCNNRDNGTETYTVEGATDNSGTYRLPVAGDHEDDICEVRL
VESSRPDCNEPFRSIDSARILLTKNVGVVDKTRYPNALGYMKKEAQPEC
>Potri.017G100600.2
GTVFCDTCFQEAFSRNSHFISGASVAVECKDEESRPGFREEVKTDEHGEFKVHLPFSVSKHVKKIKRC
SVKLLSSSEPFCAVASSATSSSLHLKSRKQGTHIFSSGFFTFKPEKQPILC
>Potri.009G129900.2.
GIGECADCAQSNIKTVHAFSGLKVTIDCKPENGEFKTRGVGELDEEGKFKVSLQNDVVKDGKLKEECY
AQLHSASAAPCPAHNGLESSKIVFKSKTDEKHTFGLAGKLKFSPVTC
>Potri.004G169200.2
GTVFCDQCKDGQVSLFDYPIYGIKVTMTCADASGQITMSREETTNWFGNYVMTFDGSPDLSNCYAQV
SSSGQGSNACGAAAGPAQKLRLTFRMFDMEFYAVDSLLTEPSASMSFC
>Potri.004G168600.2
GTGECADCAESNIKTVHAFSGLRVTIDCKPENGEFKTRGFGELDEEGKFKVSLPSEVVKDGKLKEECY
AQLHSASAAPCPSHDGLESSKIVLKSKTDEKHTFGLAGKLKFSPVTC
>Potri.004G114300.2
GTVYCDTCFQEYFSRNSHFISGAHVAVECKDEKSRPSFREEAKTDEHGEFKVHLPFSVSKHVKKIKRC
SVELLSSSEPYCAVASTATSSSLRLKSRKEGTHIFSAGFFTFKPEKEPFLC
>Potri.004G114400.2
GTVYCDTCFQEYFSRNSHFISGAHVAVECKDEKSRPSFREEAKTDEHGEFKVHLPFSVSKHVKKIKRC
SVELLSSSEPYCAVASTATSSSLRLKSRKEGTHIFSAGFFTFKPEKEPFLC
>Potri.004G044700.3

GVVYCKSCNYSGVDTLLGAKPVLGATVKLQCNNTMKPQDVKTTTDKNGYFLIKAPGTITNYGVHKCK
VWLVSAPSTACSKITNLHGGLTGAMLRPEKKPFVDEKKREFALFSVGPFAFESKC
>Potri.011G053600.3
GVVYCKSCNYSGVDTFLGAKPVPGATVKLQCNNTKYPLEVKATTDKNGYFLAKAPGTITNYGFHKCK
VWLVSAPNTACSKITDIHGGLAGAILRPEKKPFVDEKKRGYALFSVGPFAFESKC

Myrtales

>Eucgr.E02748.1
GKVYCDTCRTQFITKVSKDMPDAKVRLECKDREGGSVTYSIEGVTDNTGMYRLPVDGEHEEEICEIVL
VKSSQPGCEEVSNDPFLRKTARISLTKNNGMATPVRQANPLGYMKNESLPEC
>Eucgr.F00490.1
GKVYCDTCRAGFETPASTYIAGAKVKVECKDRTSMKLLYSQEATTDSTGTYKLFVSEDHQDQLCDAM
LLSSPQLNCQKPAAGRDRARVILTRYNGIVSDTRYANNMGFEMDQPLAGC
>Eucgr.F03264.1
GKVFCDTCRVDFETKVSYGLADSKVRLECHNRTSGALTLSAEAVTDKTGEYRLEVEGDHEEEICEVQ
LIESSEPECSSLLEGAVHNARVALTNKNGVAQPARFANALAFAKKETLADC
>Eucgr.G03079.1
GMVYCQSCKYFGTWSLDEAKPVGSAKVSVVCKNYKNRVTYYKTFQTEESGYFYAELKGYEMSNPIL
DHPLHACHVKLVSSPLEGCNLLSNINYGMYGAPLRYEDKTLYRKEYEVVVYAAGPLAFRPSYC
>Eucgr.H04813.1
GSVYCDTCRFGFETPASTHIEGAAVRVECKDSTGTQLDYSIDTVTDSNGKWEVTVEDDHDDQLCSAV
LVSSPKAGCQTVDPGRSKATLILTRSNGAISSRHYANSMGCLTDQPLAEC
>Eucgr.L01869.2
GAMECSDCAQRNVKLSQAFSGIRVSIECKTAKGKFEHRAVGELDGEGKFQVSLPEEMVEGGKLKEE
CYAQLHSAQATPCPAHDGLESSKIVLKSKANGKHTFGLSGKLKIASSTC
>Eucgr.I01377.2
GIGECADCKRNNINAKQAFSGIRVSINCLTPKGEYKRRGVGELNEEGKFKVLLPPEIFVEGKSKEECYA
QLHSAAATPCPSYGSPKSSKIVFKPVTHTKRTYGLADKLKFSPATC
>Eucgr.G01761.2
GTVICDQCKDGRMSIFDYPIYGIKVMVACTNSDGQVTMSREETTNWFGNYGMRFEGTPDLSSCHAQ
VMGGSGQGSMGCSASASPAQPLRLTFQMFNMEFYSVDTLLSQPAQPMSFC
>Eucgr.J01410.2
GTVYCNTCFTQGFSKASHFIPGASVAVECKDGSAKLSFHKEAKTDEHGEFKVSLPFSIGKHVRRIEGC
SVKLISSSEPNCAVASPVTSSSLHFKMRKNGDHVFSAGFFTFKPLQRPKLC
>Eucgr.E03269.3
GVVYCKSCEYTLNGAKPIIGAVVKLWCKNTKYPAWATATTDKNGYFFLEAPKTVSNFATHKCKVFLVS
SPIPSCSEPSNLNGGSSGAPLKFEKLVLGKPLFTLYSVGPLAEPKC
>AYMT_2111070_Eucalyptus_leucoxylon.1
GRVVCDVCGDSSIGPEDHVLQGAEVAVLCITKSGEVVNYQAFTNSKGVYKVAETMPESDRWDACLA
RPISSFHEHCTQLGGGTAGVKFSYNHPSGYFHTVRPFVYRTVNVPTYC
>AYMT_2010734_Eucalyptus_leucoxylon.2
GTVFCDTCFKQEFSRTSHFISGAYVSLECGNSSSSLSFRQEARTNRHGKFKVLLPPSMGKRREAEGI
EGCTVKLIKSGDPGCHAVSLGISSSLRLKAKRHGLQVFSAGLLAFKPLKQPSRC

Malvales

>Gorai.009G368100.1
GRVYCDTCRVEFETKISKPIKGASVKLECRNITNEKIVSHSQDVVTDEAGGYKIEVKGDHEDEICEVSL
VKSPRADCSEPTEVWRKARVVLTKADGVSGIYRFANNLGYMKKEALPEC
>Gorai.009G368200.1
GHVYCDTCRVEFETKLSEPISGAVVKLECRNRTNEAITFQSKEIVTDNHGDYHVIVEGDYEESDCDVAL
VRSPRADCSDPTEAWRKSRVVLTTFDGLSGKLRFANNLGFKKDIALPQC
>Gorai.009G282200.1
GQVYCDHCRAGFETPKTRNMAGAKVKVVCSNRKTGDVVYEKEGHTDSTGQYKIAVSEDHLDEICDA
VLVKSSQPECAEMSPGRERARVVLTNFNGISSNTRFANAMGFMANKAEAGC
>Gorai.004G272200.1
GKIYCDTCRVEFETKLSEPIPGAKVKLECRKQGDETVTYSHEAETDANGVYKIPVEGNHEEQICEVSTV
SSPRADCNEKMEAFEKAQVELTSDDGLAEPSRKANNLGFKKKVAVPGC

>Gorai.002G061500.1
GKVYCDTCRVEFETKLSQPINAKVHLECKNRTDERIVYSKDAVSDKLGMYSIPVEGDHEDELCEVRLIE
SPRNNCNEMMESWRKARVVLTRRDGVTDLTRQTNNLGFKIKPEDVDTKAC
>Gorai.002G087500.1
GRVICDVCADSSIGPEDHILEGAEVAVLCITKSGEVVNYQAFTNAKGIYTVAETMPESDRWNTCLARPI
SSFHDHCNHLGDRSTGIKFTYSRPSGHFHTVRPFVYQPSTAPSYC
>Gorai.002G160400.1
GKVMCQDCTKSYGEWTHGSQPIKGGKVSVTCKDDRSRIIYYASDESDEEGSFNMAVNKYINGKELQP
TSCLVRLVSSPHLTCNIPTNFAGGITGVNLPVRPTVLYRDLVQYQLGTFFYTTPRC
>Gorai.005G093100.1
GYVYCDTCRAGFETPKTRSLADAMVRLVCSDRKSGQVVFKKEGYTDKTGKYQIRVSEDHLDQICDAV
LVKSSQLGCATMSPGRERARVILTNFNGISSTTRYANAMGFMVDEPEAGC
>Gorai.011G291100.1
GMVYCQSCDNYGSWSLSKAKPIASAKVSVICKNQRGQVSYYKASETDGNGYFFAELQGFEMSHLLL
DHPLQSCVVKLVESPLEGCNVLSNINYGLYGSPLRYENKRFYRKDYDVVIYAAGPLAFRPAHC
>Gorai.012G153300.2
GVGECTDCKENNLDTTQAFSGLRVTIDCKPENGDHFKTRGSGKLDKQGNFKVSLPQHLFKDGKLNE
HCYAQLRTVSSPQPCPSINGLESSKLVFKSTTDQKHQFGLKENLKFSPITC
>Gorai.001G206700.2
GTVYCDTCFQSDFSRPTHFISGATVAVECKDGKSSRASFRQQVKTNRHGEFKVHLPFSVSKHVKKIE
GCEVKLIKSSEPYCAVASSATSSSLHLKSRKQGTHVFSAGFFTFKPFKQPTLC
>Gorai.001G206900.2
GTVYCDICFQQDFSKAHHFISGASVAVECKDGDSRPSFRQVAKTNEQGEFKVRLPFSVTKHVKKVKG
CSVKLVRSSEPYCAVASSSTSSSLHPKSTKQGTKVFSAGFLSFKPLKLPNLC
>Gorai.002G193700.2
GAVFCDQCKDGQRSLFDYPLSGMKVTVACADGTGQVTMSREETTNVFGNYVMRFDGTPDLSNCNA
QVSGSGEGSNDCGATAGPAQKLRLMFRMFGMEIYGVDSLLSEPSQPMSFC
>Gorai.002G193500.2
GAGECADCAENNLEISQAFSGLRVSIDCKPENGKNFKTRGSGELDKQGNFKVFVPEDLVENGELKEE
CYAQLHSVSAAPCPAHDGLESAKLVLKSRSDGKHEFGLKGKLRFSPLTC
>Gorai.010G154300.3
GVVFCKSCKYAGSDTLLGAKPILGATVKLTCKNTKYKQEATAKTDKNGYFFLQAPKTVTSFGAHKCTV
SLVSSPMAKCSKPSNLHGGLKGATLRPEKPFTANKLPFILYTVGPFAFEPKC
>Gorai.009G168900.3
GVVYCKSCKYAGVDTLLGAKPIPRATVRLTCKDAKNELTVQFKTDKNGYFFLQAPITIYNFDLHNCSVS
LVSSPLKACSKPSNLNGGLKGAPLKPEKPSTSKKLPYVLYSVGPFAFEPTC
>Gorai.005G018000.4
GKVYCYRCYDSSYPEKSHGKKHLEGATVEVTCKEGEKEITVYGKTKNNGKFAVTVDGFDYGKYGAK
ACIAKLHAPPKDSSCNIPMSVHDGIKGAVLKVKSEDKYEVVLRAKPFGYGSKKPYKKC
>Gorai.006G227800.4
GKVYCYRCYDWSYPAKSHNKKHLKDAVVEVTCKVGEKEITAYGKTKINGKFSITVEGFNYAKYGAEAC
KAKLHAPPKGSSCNIATGLHGGNKGAMLTVKSKDKYEVVLKAEPFAYAPKTPYKEC
>Gorai.013G160500.1
GRVVCDVCADSSIGPEDHVLEGAEVAVLCITKSGEVVNYQAFTNSKGIYTVAETMPESERWDACLAR
PISSFHDHCNHLGDGSTGIKFTYNHPSGHFHVIRPFVYRPSTAPTYC

Brassicales

>AT1G78040.1
GSTYCDICKFGFETPESSYFIPGATVKLSCKDRKTMEEVYTDKAVSDKEGKYKFIVHDDHRDQMCDVL
LVKSSDKTCSKISVGREKSRVILNHYSGIASQIRHANNMGFEKEVSDVFC
>AT1G29140.1
GSVYCDTCRVQFITRISKFLEGAKVKLECKGRENQTVTLTKEAVTDNAGNYQMEVMGDHEEEVCEIVL
LQSPDPECGDVNNQEFLRNAARISLTANDGIVSNETRTINPLGFMRKTPLAEC
>AT3G09925.1
GKVMCQDCSLNYDEWINGSEPIKGAVVSITCMDERRRVSYYGSDLTDERGQFDLMVNKVLSHGKVL
KPQLCNVRLVSSPDLSCNIPTNFGNGQTGVKLVRPFTVFRDLVKYVVGPFYYTTPMC
>AT4G08685.1
GRVYCDTCLAGFETPASTYISGAVVRLECKDRRTMELTYSHEARTDSTGSYKILVNEDHDEQFCDAM
LVRSSQLRCSNVSPGHDRARVTLTRFNGIASDDRFANNMGFLRDAAMPGC
>AT5G45880.1

GSVYCDTCRVQFVTRLSKFLEGAKVKLECRSRTNGTITLTKEAVTDKTGSYKMEVTGDHEEEVCELVL
VQSPDSGCSDVSTEAYLRNAAKISLTANDGIVSHETRIVNPLGFMVQTPLADC
>AT5G10130.1
GSVYCDTCRFGFETIATQYIRGARVRIVCKDRVTLKSELVGVAVTGPDGKYKVAVRGDRQDQQCLAE
LVHSPLSRCQEADPGRSTATVILTRSNGAASTRHYANAMGFFRDEPLRGC
>AT5G54855.1
GRVVCDVCADSSIGPEDHVLEGAEVAVLCITKSGEVVNYQAFTNSKGVYTVAETMPESERWDACLAR
PISSFHTPCNRLHQTNTGIKFTYNRPSGFFHAVKPFVYRPQYAPSYC
>AT5G15780.2
GTVYCDTCFNGAFSKSPNHLISGALVAVECIDENSKPSFRQEVKTDKRGEFKVKLPFSVSKHVKKIKR
CSVKLLSSSQPYCSIASSATSSSLKRLKSNHHGENTRVFSAGFFTFRPENQPEIC
>AT2G16630.2
GSVFCDQCKDGERSLFDFPVSGIKISVTCADENGQVYMSREETTNWLGGYVMRFDGTPDLSNCYAQ
VSDNGVQQDPSSCSIASGPAQKLKLMFSFFGIETFAADALLAQPVQPSSFC
>AT1G28290.3
GTVYCKSCKYAAFNTLLGAKPIEGATVKLVCKSKKNITAETTTDKNGYFLLLAPKTVTNFGFRGCRVYL
VKSKDYKCSKVSKLFGGDVGAELKPEKKLGKSTVVVNKLVYGLFNVGPFAFNPSC
>AT2G33790.3
GVVYCKACKYAGVNNVQGAKPVKDAVVRLVCKNKKNSISETKTDKNGYFMLLAPKTVTNYDIKGCRA
FLVKSPDTKCSKVSSLHDGGKGSVLKPVLKPGFSSTIMRWFKYSVYNVGPFAFEPTC
>AT2G34700.3
GMVYCKSCKYSGVDTLLEASPLQGATVKLACNNTKRGVTMETKTDKNGYFFMLAPKKLTTYAFHTCR
AWPTNPGPTTATMTCTVPSKLNNGITGAMLKPSKTINIGEHDYVLFSVGPFAFEPAC
>AT5G05500.3
GMVYCQSCDKFGSWSLAGAEAIAGAKISIICKNHRQQVSFYKVFRTDSYGHFYGELKGFKMTPHFLD
HPLHSCRAKLVSSPREDCNLFSNINNALDGAPLRYEEKRLKWTNYEAVIYAAGPLAFRPDHC
>AT4G18596.1
GSVYCDTCRVQFVTRLSKFLEGAKVKLECRSRTNGTVTLTKEAVTDKTGSYRMEVTGDHEEEVCELV
LVESPDSGCSDVSKEAYLRNAAKISLTANDGIVSHETRIVNPLGFMVQTPSAEC

>Plantago_lanceolata_CAC41635
GEVYCNVCHSRNLINELSERMAGAQVQLDCKDDSKKVIYSIGGETGQDGVYRLPVVGYHEDCEIKLV
KSGRPDCSEIPKLAKGTIQTSKVDLSKNTTITEKTRHVKPLSFRAKTDAPGC
