# Supplementary material for: The Cell Wall PAC (Proline-Rich, Arabinogalactan Proteins, Conserved Cysteines) Domain-Proteins Are Conserved in the Green Lineage
Source: Int J Mol Sci. 2020 Apr 3;21(7):2488. doi: 10.3390/ijms21072488 (PMC7177597; doi:10.3390/ijms21072488)
Supplement: Supplementary file 1 [file ijms-21-02488-s001.zip › Supplementary files_R1_Nguyen-Kim, San Clemente et al/Nguyen-Kim, San Clemente et al._Supplementary file 3.pdf]

**Supplementary file 3.** Some examples of PAC domain-related proteins containing predicted functional domains suggesting intracellular functions.

The sequences were retrieved from https://db.cngb.org/onekp/. They were analyzed using the InterproScan software (http://www.ebi.ac.uk/interpro/search/sequence-search).

Originally, the accession numbers were of the following type: #onekp:YFGP_scaffold_2007785 Pallavicinia_lyellii. They have been simplified for more convenience, as follows: >YFGP_2007785_Pallavicinia_lyellii

## *Marchantiophyta*

>YFGP_2007785_Pallavicinia_lyellii
KRSFPKYKEADSSSESESDFTRPARDTAMKPTGLPIGVVRGCPKCSDCQAVLARWRPEEGCKPVVDAAPVFYPT
EEEFKDTLKYIAKIRSAAEPFGICRVVPPPGWQPPCPLREDTAKLHVMKFPTRVQEIHKLQVRQPVKKCSQARQRL
GGRRGRGGFRGRMGRPCRRTPNLSTTKSVQPDESFGFEPGSYFSLSKFEKYAQQFKEQYFFRADAADELQSKS
EIGSDSTSSMSPSVDAIEAEYWRIVEKPTEQIEVLYGADVETGSFGSGFPKGKANATEPYEKSGWNLNNIARLPGS
MLAFEEGDISGVLVPWLYVGMCFSSFCWHVEDHHFYSLNYLHWGAPKVWYGVAGHAAHDMEAVMKEHLPELF
DEQPDLLHKLVTQLSPSILRREGVPVYRLVQQAGEFVITFPRAYHSGFNCGFNCAEAVNLAPVDWLPHGQGAVEL
YREQRRKTSVSHDKLLLAAAREAAKVLVHSRNQPGLQKLPMQVVKQEFIEEEEKKGVSEGDADVLYAWQTFCGQ
DGVLARALKARVELECSRQTSLTGDVLMKKMDCWYDATDERECAECHYDLHLSAVGCECCPEKFVCLAHASLLC
VCHWGRKFVLYRYSLAELELLVAALEEQPGAMSTWLEQEQRSSSLSISPFLHQDTGLAQTLGPCEFNSTVFPCNR
NVFKAQDVESRLESQTQYMASRSNVPGTSDNPLSHQGSRGVNTQSTVSLQHSSKAILSNTAQNLPESHTAASGL
DERKINSVGVQTRGQLAMYSERKRMLTNSMKTAKQIGETSIVKSRQEVIVLSDDEEANLKKTNMIVSSITKSHEGS
NAVLHAINSKTAFLDTARSSASCKQSTSFTEAECALSGTCLERQLVVPDGATLASVVPQLKLNLPRSKSAQLPDTS
STPMVRAQVHHHVVATEESPVSSPPYVLRGRSIGNLKQQARAASTQHLGSGLRTYSRADHAQRLQIGPEASASEI
CGHIPVPDSFTPAVNAVQSRTPARLDRPVGPRVAKVVRKKVCREVELLNVGSLVLREGWHDKHCIFPAGFKSRTS
FCDVLNISQNCFYVSEIVEYGSSGPQFKVSVEGNSLAEFVNTSIDLCWRTVQEKINEEIIRCRGIGETNLPPLQPPQ
SLHGLDMFGLLSPMIVQAIEALDPQHLCCEYWTAK

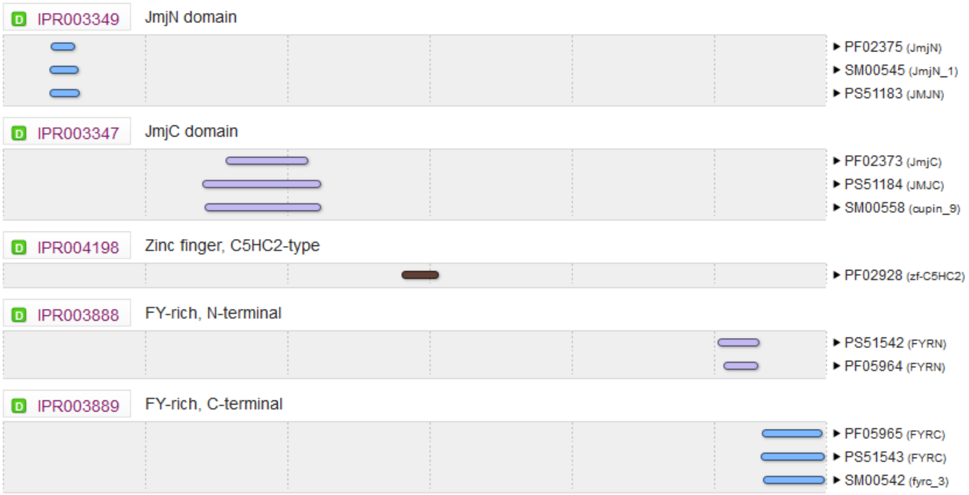


The JmjN and JmjC domains are two non-adjacent domains which have been identified in the jumonji family of transcription factors. Although it was originally suggested that the JmjN and JmjC domains always co-occur and might form a single functional unit within the folded protein, the JmjC domain was latter found without the JmjN domain in organisms from bacteria to human [PMID: 10838566, PMID: 11165500]. (http://www.ebi.ac.uk/interpro/entry/IPR003349)

>ILBQ_2007285_Conocephalum_conicum
PGVSQPGSPAASGGPPPSHPSRPTSNIDPNQIPRPQPSASHVIFETRVNGQANLPPSATTAFVVRDTGNCSPRLM
RSTLNQIPCSGDLLNTSGMPLAVMVQPFALPHPSEEPIQVVDFGETGPVRCSRCKAYINPFMKFIDQGRRFTCNL
CGFTNETPRDYHCNLGPDGRRRDADQRPELSRGTVEFVASAEYLVRPPMPAVFFFLIDVSMNAVQTGAVAAACS
AINRALSDLPEGPNTLVGIATFDSTIHYYSLNRNLQQPAMLVVPDIQDPYTPLQTDLIVPLSEGKDHLEQILESIPSMF
QNNRVAESAFGAAIKGAYLAMKSTGGKLMVFQSVLPSVGLGALPPRESEGRVSGEKEALKLLQPADKLLKTMAIE
FAEFQVCVDLFMTAQMYVDIASLSVVPRTTGGQLYFYQPFYAGADSAKLYNDLRWNVTRPQGFEAVMRVRCSQ
GLQVQEYYGNFCKHIPTDVDLPAIDSDKTIMVTFKHDDKFQDGSECCFQCALLYTTVTGQRRIRVITLSLSCTTVLS
NLFRGADLDAQFTYFLKHSAQEIPTIPLTQVRDQMTAQCVNILFTYRRYCATASSAGQLILPEALKLLPLYTLALTKSI

GLRADTRVDARFDDRSYW**C**TRVASLAGSLSIPLVYPRMFAIHNLPPKESLADGDLPPVVPLSSENLDPDGVFLLET
GEDAFLYVGKQAPVEVLEQLFGVHTVDEVVPGQFMLQEYDNPASKRLNAIINEIRRQRCSYLRLRLLRRGDPLEFL
FFSYLVEDKSSSGLSYVEFLVYYVHKQIQKRM

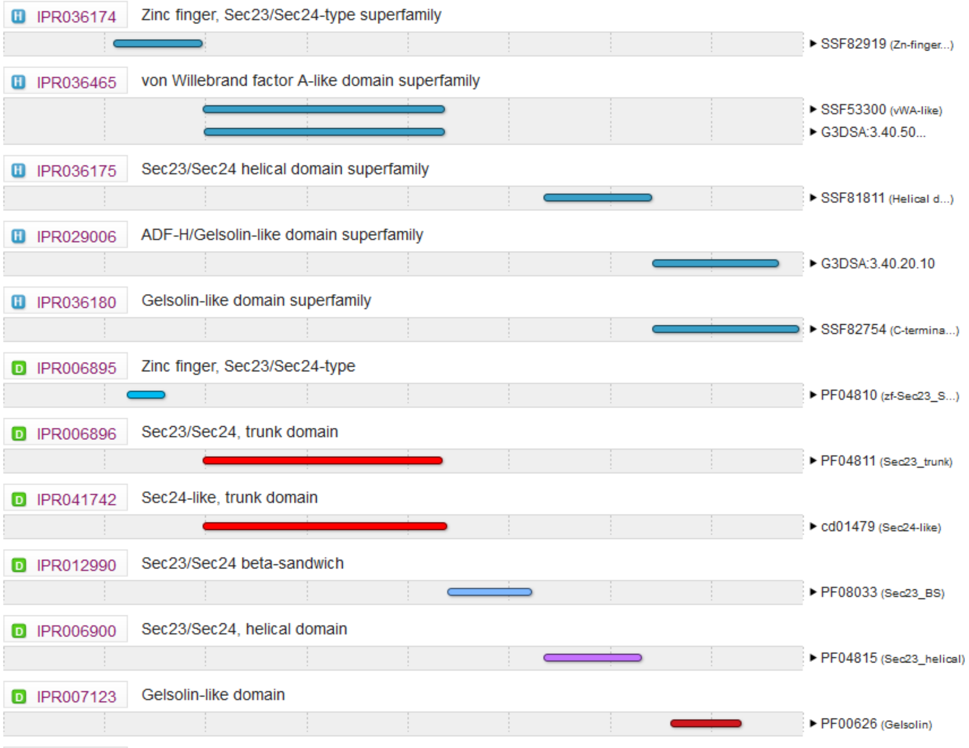


Gelsolin is a cytoplasmic, calcium-regulated, actin-modulating protein that binds to the barbed ends of actin filaments, preventing monomer exchange (end-blocking or capping) [PMID: 3023087]. It can promote nucleation (the assembly of monomers into filaments), as well as sever existing filaments. In addition, this protein binds with high affinity to fibronectin. Plasma gelsolin and cytoplasmic gelsolin are derived from a single gene by alternate initiation sites and differential splicing. (http://www.ebi.ac.uk/interpro/entry/IPR036180).
COPII (coat protein complex II)-coated vesicles carry proteins from the endoplasmic reticulum (ER) to the Golgi complex [PMID: 11535824]. COPII-coated vesicles form on the ER by the stepwise recruitment of three cytosolic components: Sar1-GTP to initiate coat formation, Sec23/24 heterodimer to select SNARE and cargo molecules, and Sec13/31 to induce coat polymerisation and membrane deformation [PMID: 12239560]. (http://www.ebi.ac.uk/interpro/entry/IPR012990).

>WJLO_2007937_Riccia_berychiana
GGPPSGPPPGARPAAPPFATPNAPGAAPGQVRAGPPFPGGFGPPSQPGMAPSNALRGPPPGPPGVPAVAGVS
MPPAPPGVAFPGRPGFPQMTNAHPLGPSAQPQLASMAQPPQRPPGPPFATPAVQGVATGTYSGGGPSVTSRP
AAQPGGPFSPSSAPMPPYGQPPQAPTAQMQNLSLQNQGIPGGAPPRSFAPPTQGGVQTSAPTSWQAPPRRVY
PDFGPPTGTQTGQAFSQIPSHLPPNVGHVTGVSDKYGSTVPGMPQPGSPATSAAQPPSHPSRPTSNIDPNQIPR
PQPTASHVIFETRVNGQANLPPSATSAFVVRDTGNCSPRLMRCTLNQIPCSGDLLNTSGMPLAVMVQPFALPHPS
EEPIQVVDFGESGPVRCSRCKAYINPFMKFIDQGRRFTCNLCGFTNETPRDYHCNLGPDGRRRDADQRPELSRG
TVEFVASPEYLVRPPMPAVFFFLIDVSMNAVQTGAVAAACSAINRALSDLPEGPNTLVGIATFDSTIHYYSLNKNLQ
QPAMLVVPDIQDPYTPLQTDLIVPLSEGKDHLEQILESIPSMFQNNRVPESSFGAAIKGAFLAMKSTGGRLMVFQS
VLPSVGLGALTPRENEGRVSGEKEALKLLQPADKVLKTMAIEFAEFQVCVDLFLTAQMYVDIASLSVVPRTTGGQV
YFYQPFYAGADSAKLYNDLRWNVTRPQGFEAVMRVRCSQGLQVQEYYGNFCKRIPTDVDLPAIDSDKTIMVTFK
HDDKFQDGSEC**C**FQ**C**ALLYTTVTGQRRIRVITLSLS**C**TTVLSNLFRGADLDAQFTYFLKHSAQEIPNVPLTQVRDQ
MTAQ**C**VNILYTYRKY**C**ATASSAGQLILPEALKLLPLYTLALTKSIGLRADTRVDARFDDRSYW**C**TRVASLAASLSIPL
VYPRMFAIHNLPPDSQAEGELPPVVPLSSENLDPDGVFLLETGEDAFLYVGKQAPVELLEQLFGVHSVDEVVSGQ
FILQEFDNPASKRLNAIVNEIRRQRCSYLRLRLLRRGDPLEFLFFSYLVEDKSSSGLSYVEFLVYVVHKQIQKRM

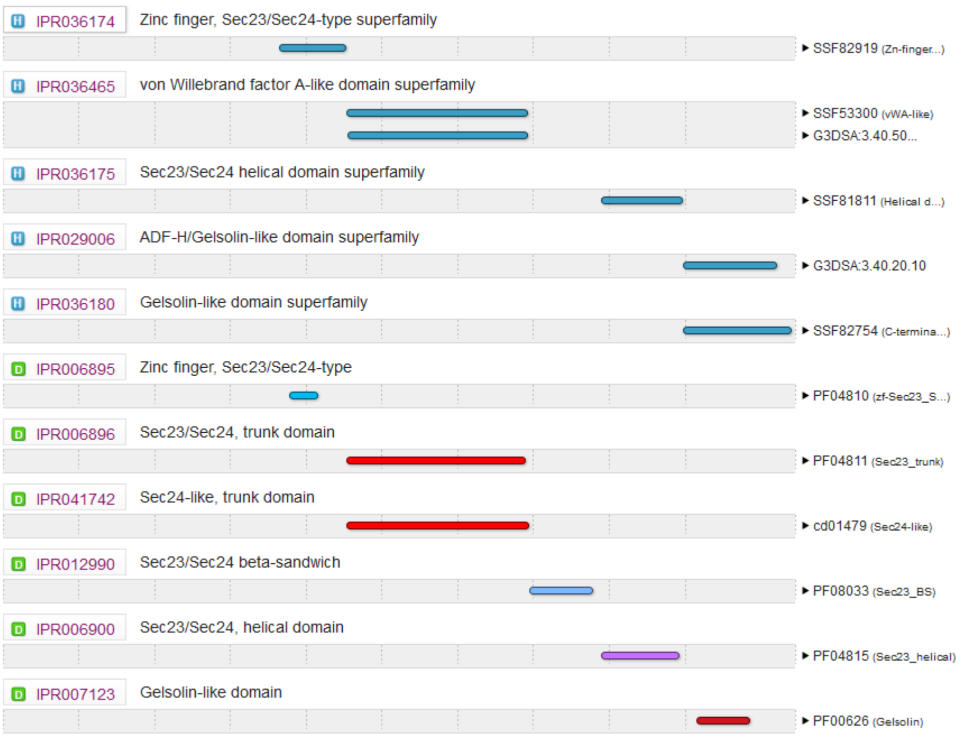


## *Bryophyta*

>HVBQ_2004216_Tetraphis_pellucida
QMELGGKDVCIICEDADLDLAAKNIIKGGFSYSGQRCTAVKLILVVESVADKLVDMVSKAVDGLSVGKPEDNCTITP
VISKSSADFIEGLVKDCEEKGAKLLQKYRREGNLIWPLLIDHVTPFGPVVPVVRVKTVEEAVDHCNANNLALQGCV
FSRDIDQAIRVSDAMETGTVQVNGAPARGPDHFPFQGFRDSGIGSQGIKNSLAMMVKTKTTVLNLRQPTYTMG

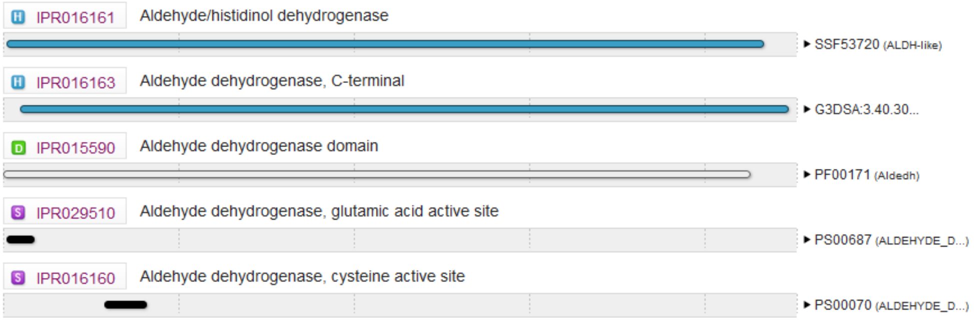


Aldehyde dehydrogenases (EC:1.2.1.3 and EC:1.2.1.5) are enzymes that oxidize a wide variety of aliphatic and aromatic aldehydes using NADP as a cofactor. In mammals at least four different forms of the enzyme are known [PMID: 2713359]: class-1 (or Ald C) a tetrameric cytosolic enzyme, class-2 (or Ald M) a tetrameric mitochondrial enzyme, class- 3 (or Ald D) a dimeric cytosolic enzyme, and class IV a microsomal enzyme. Aldehyde dehydrogenases have also been sequenced from fungal and bacterial species. A number of enzymes are known to be evolutionary related to aldehyde dehydrogenases. (http://www.ebi.ac.uk/interpro/entry/IPR015590).

>WSPM_2007928_Rhytidiadelphus_loreus
KKFRRKSRVSYRESDSSSEGENGPVRLPHAVSTKSELPIGVLRGCPS**C**RT**C**QKVVATWRPEASRRP**C**IEDAPVF
YPNEEEFQHPLRYIASIRAKAEPYGI**C**RVVPPQSWKPP**C**PLRGDSATVQSMQFPTRVQQVHKLQVRQPTAME**C**S
PTKQASKKRRGRAPMGRMGGAEVYAPSPPVNVQPEYFGFWPGEPFPLGDFEKYANDFKNSASLSDKELAQEW
EPTVEQIEGEYWRIVEQATDQIEVLYGADVETGKFGSGFPRADPRSEATSIYENSGWNLNNIARYPGSMLSFEEG

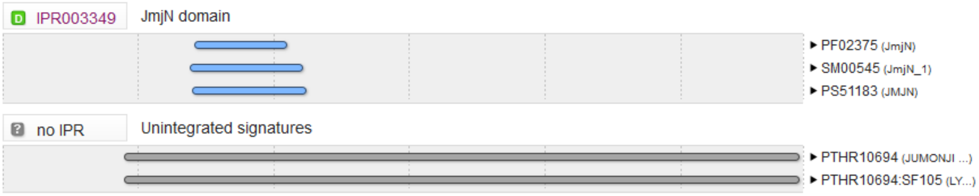


>ZTHV_2082727_Atrichum_angustatum
MGAFGCLICVAQSTVGVVEQWGRFSQIAQPGLHCLNPFAGEWLVGTLSLRVQSLDVRCDTKTKDNVFVTVVCSI
QYRVVRQNADDAFYELQNPREQIQAYVFDVVRACVPKMILDDVFEQKDDIAKAVSEELEKVMGAYGYSIEQTLIVD
VEPDATVRRAMNEINAAQRMRMAAMDKAEAEKILQVKRAEGEAEAKYLSGSGIARQRQAITEGLRESVLTFSNNV
PGTSAKDVMDLVLLTQYFDTMKEIGNSSKNTTIFLPHGPGHVGDITEQIRNGMMQGS

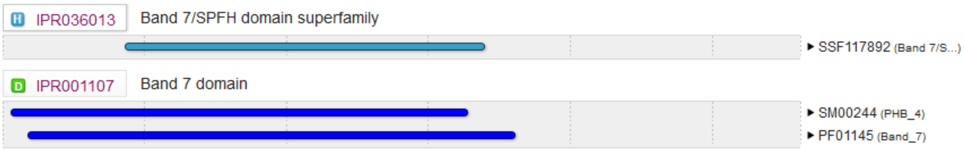


The band-7 protein family comprises a diverse set of membrane-bound proteins characterised by the presence of a conserved domain, the band-7 domain, also known as SPFH or PHB domain [PMID: 10542406]. The exact function of the band-7 domain is not known, but examples from animal and bacterial stomatin-type proteins demonstrate binding to lipids and the ability to assemble into membrane-bound oligomers that form putative scaffolds [PMID: 24782879]. A variety of proteins belong to this family. These include the prohibitins, cytoplasmic anti-proliferative proteins and stomatin, an erythrocyte membrane protein. Bacterial HflC protein also belongs to this family. (http://www.ebi.ac.uk/interpro/entry/IPR001107).

>WNGH_2001339_Aulacomnium_heterostichum
MGGIGCLICVGQSTVGVIEKWGKFSGIAQPGLHCLNPFAGEWLAGRLSLRVQSLDVRCDTKTKDNVFVSVICSIQY
RVVRQNADDAFYELQNPREQIQSYVFDVVRACVPRMILDDVFEQKNDIAKAVSEELEKVMGAYGYSIEQTLIVDVE
PDATVRRAMNEINAAQRMRVAALDKAEAEKILQVKRAEGEAEAKFLSGHGIARQRQAITEGLRESVLLFSNNVPGT
TSKDVMDLVLITQYFDTMKEIGASSKNTTVFLPHGPGHVSDITQQIRNGMMQGSASA

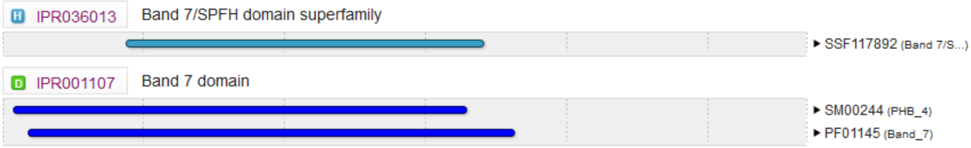


>YWNF_2049992_Hedwigia_ciliata
MGGLGCLVCIGQSTVGVIEKWGKFSGIAQPGLHCLNPLAGEWLAGRLSLRVQSLDVRCDTKTKDNVFVSVICSIQ
YRVVRQNADDAFYELQNPREQIQSFVFDVVRACVPRMLLDEVFEQKNDIAKAVSEELEKVMGAYGYSIEQTLIVDV
EPDATVRRAMNEINAAQRMRVAALDKAEAEKILQVKRAEGEAEAKYLSGHGIARQRQAITEGLRESVLLFSNNVP
GTTSKDVMDLVLITQYFDTMKDIGASSKNTTVFLPHGPGHVGDITQQIRNGMMQGSAS

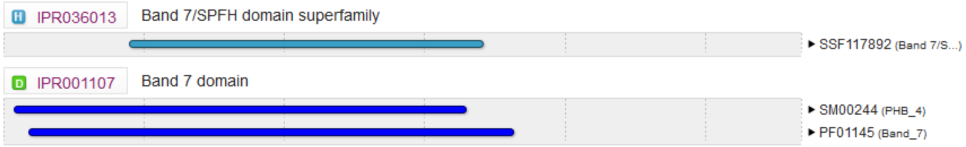


>TAVP_2009233_Calliergon_cordifolium
MGGLGCLVCIGQSTVGVIEKWGKFSGIAQPGLHCLNPFAGEWLAGRLSLRVQSLDVRCDTKTKDNVFVSVICSIQ
YRVVRQNADDAFYELQNPREQIQSYVFDVVRACVPKMILDDVFEQKNDVAKAVSEELEKVMGAYGYSIEQTLIVD
VEPDPTVRRAMNEINAAQRMRVATLDKAEAEKILQVKRAEGEAEAKFLSGHGIARQRQAITEGLRESVLLFSNNVP
GTTSKDVMDLVLITQYFDTMKEIGAGSKNTTVFLPHGPGHVGDITQQIRNGMMQSSASA

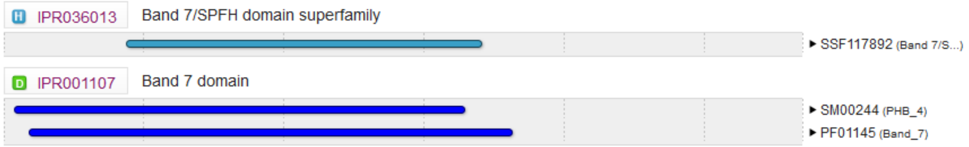

## Psilotales

>ALVQ_2020591_Tmesipteris_parva

YTSSSTAGQVITCRAAVAWKPKQPLVIEYVQVDPPQAGEVRIKITHSSLCHTDVFYWEAEVGETSPNDAEHLIIKNC
GPKSNMTQELQELNLSMNFKICLFKFHAHLSMFPRIFGHEAAGIVESIGEGVTDIREGDHVIPLFTGECGNCKYCKS
DKTNLCSKLRVNPGRVVMLRDQKTRFSFKGQPIYHFLNTSSFSEYTVVDYGCVVTVNFKAPLERICLFACCIPTGG
AVWNTAKMEVGSAIGIFGLGAVGLAVALGARISGASRIIGVDINPKKFPRAKAFGVTDFVNPNDFENPIEEVLRELS
NGGLDYCFECVGNVDVMRAAFQSCHDGWGKTILVGIEASNKQLVFNPMQFFDGREIKGCTFGDFKGRTQIPNLV
DTCLTKPDQLDLNAFVTHELPFSRINESFQMLKEGQSLRCVLRM

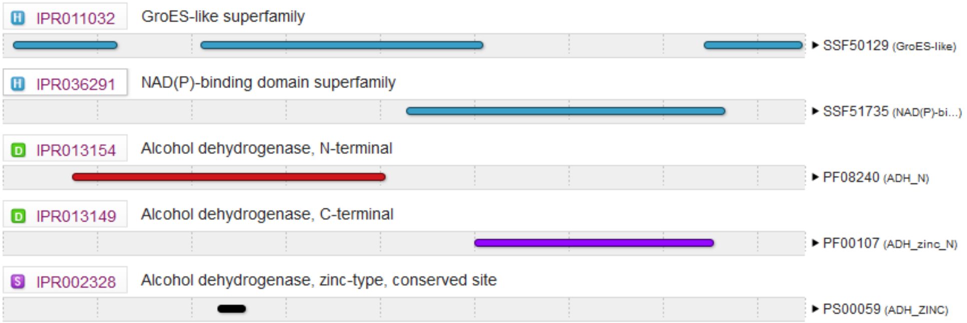


This is the catalytic domain of alcohol dehydrogenases (EC:1.1.1.1). Many of them contain an inserted zinc binding domain. This domain has a GroES-like structure; a name derived from the superfamily of proteins with a GroES fold. Proteins with a GroES fold structure have a highly conserved hydrophobic core and a glycyl-aspartate dipeptide which is thought to maintain the fold [PMID: 10556240, PMID: 8804825]. (http://www.ebi.ac.uk/interpro/entry/IPR013154).

>ALVQ_2017464_Tmesipteris_parva
EADCGSDDEQNLVRALRYRPWISYHESETSSEDESDIGQVYPSVRISTLPVGVVRGCPSCSTCQKVLARWRPEA
ACLPVLDDAPVFFPLEEEFSDTMEYIASISAEVKQYGICRIVPPQSWRPPCPLKDFEKWKTLKFPTRLQQIHKLQVR
EPQHKRYPQSKKRRTGRPAERGLSWQRSLFSSECHKEFKCHVPDEDDEFGFYPGPDFTIEAFEKYAHQFKEEYF
GGQDINEHLGRNTVFVDPKKRWEPSIDNIEGEYWRVVEHATEQIEVLYGADIETADFGSGFPKAKPGEEASPYEK
SGWNLNNIARLP

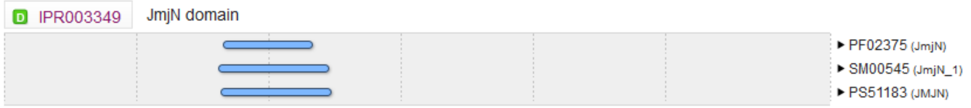


>QVMR_2011678_Psilotum_nudum
GCIRLYTVCDDKDGMIYSHSFPCVQGRILSVAWSVDATKLFSGGSDGCIRCWDVGRIQEVYRITAGLGGHGSGSD
MCVWSLLTLRDGTLVSGDSTGSTQFWDGKCGTLLQAYSCHGADVLALATSPSHHYVFAAGADGQVIQFQLVPES
SRLADRVSSSAEEVAVVLGDKWVFCGKKRMHTHDVRALAVAFPIGEGETINKATRKKNKRIKISWMEQDMWKWA
HPGVPMLISGGNDAKLFTYPANAFLAFHPHDVCFAPQQTQVKLAFEPDICNGVMLMAQYENCLDVWEVPTGGGH
SSQVKLGKGIVKSREGGDISVSKRKREAFESRNNGKHCGISNCNGVQETGMRLCELPNGTKKNCCNSNIVKQMG
KVKCPPPELLARIKCKSPNHISCSGLSGSGHLIAFSDQIKPRLCELEQQHTSDVSNRRGWSVRKRRLPRNLPPAH
NIAFSADSSRLLLACPHNSIFVVDIENVDVLHRFEVVGSELLEHADIASPITRMCTSADGQWLAVSNVLGDATIFNLE
TLRQHWSIPRLDGGPITSMMFHPRNSCILVVATRANQLHVLDVEARQFGEWSRKNASSLPQRLLEFPGGIQGLSM
SPCKMSSIIAYSSRAMCLIDFQKPVPQEKTKMTVPFTSKDKVAGSHLDLSNGSNCNLQNGGTVQSDKNFIVVPFK
DPVLFLQHLSDTSVLVIEKPWLDVLHQLPPPVYRHVYGT

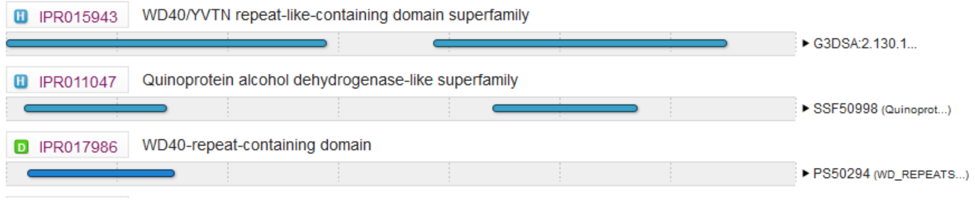


Quinoprotein alcohol dehydrogenases are a family of proteins found in methylotrophic or autotrophic bacteria. These quinoproteins use pyrroloquinoline quinone as their prosthetic group. There are three types of alcohol dehydrogenases: type I includes methanol dehydrogenase and ethanol dehydrogenase, type II includes soluble quinohaemoprotein with a C-terminal

containing haem C, and type III includes quinoprotein alcohol dehydrogenase with a C-terminal cytochrome C domain [PMID: 11761326]. (http://www.ebi.ac.uk/interpro/entry/IPR011047)

## Equisetales

>JVSZ_2014214_Equisetum_hymale
DLPDGLNTKVGIATFDSTLHFYNLNTALQQPSMLVVPDIQEVYTPLQTGLIVPLMECRSRLEQLLQAIPNMFQNTRI
PDSAFGAAIKGAALAMKSIGGKLLVFQSALPSVGLGALSAREAEGRTGPSVGEKEANKLLQPVDKTFKTLAIEFAE
YQVCVDLFLTTQSYVDVASLADVPRTTGGQIYYYYPFSATADSAKLYNDLRWNVSRPQGLEAVMRVRCSQGVQV
QDYFGNFCRRIPTDVDLPAVDCDKTFMVTLKHDDKFTEGSECCFQCALLYTTMYNERRIRVMTLSLPCTAILNSVF
RSADLDSQFTYFLKQAAQQIPNTPLPQVKEQVVTSCVNILYAYRKYCAAASSSGQLILPETLKILPLYTLAFTKSMSL
RPDVRIDDRCYWLARVASLSSSLSVAAVYPRMFAIHDLELEDLSNGSLPPFLYLSSENLGNEGIFMLENGEDVFFY
AGKSVPPDTLYQLFGVHSVNEMASNQFILQEFDNDSSRKLNALVNEIRRQRSSYLRMRWCKQGDPLEFFFLSML
VEDKNSLGDSYVEYLVRFHRQIQNKMS

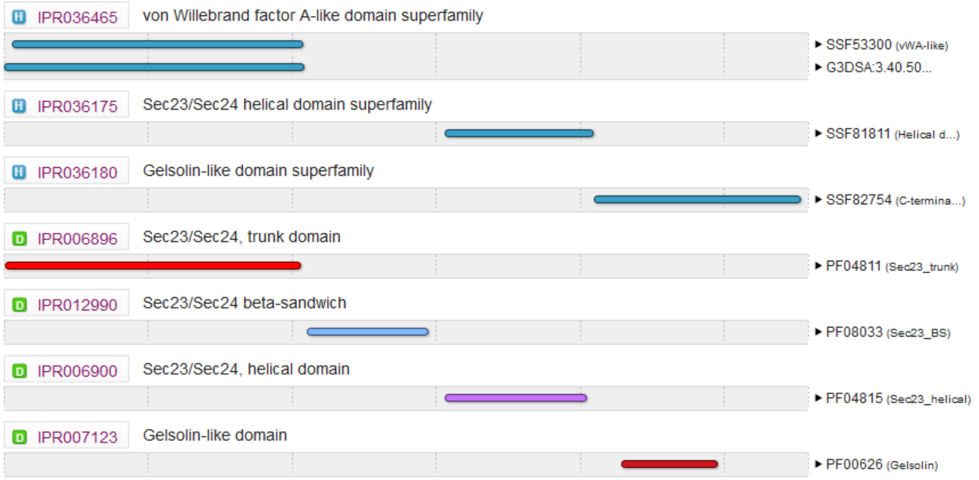


## Lycopodiales

>ENQF_2016454_Lycopodium_annotinum
ISLLWKLAGNKNAAAGVRWWASSGLACLGLYGFPSKLGENLKKMLTDSVLNDLTFVFANGNSLHCHAIMLLARCP
SLLPMFLQPDSSKQKLSRSHKYDDPISQLACMEYNDNAVEPRTQYSVDKAVKRGDPVKVQASARLSYEAMKYILE
FIYAGIVQLNAKNLTEVKLLAKRCRLKTLSGLLNGESPTWGAAIPQFSLPSLLGDLGHHFVDIVLQADTCISEQGAD
KCKLCKLTSDHVHAHRLVLSSQCEFFQALFRSGMRDSAEQAIRVHRRKNVLYYLYCGELLILSEVEGDCAWSHLG
TQRQLTYLRQLVELAELAEEWLLEDLQQQCFEIIMHYVRLNVYLCPEVMSHAATSGQGLIVEELAEIIAPMYTQMQ
NSGAFDSLEEDLRETVRTAHVRI

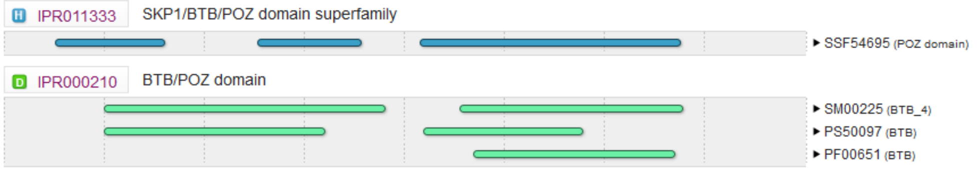


The BTB (for BR-C, ttk and bab) [PMID: 7938017] or POZ (for Pox virus and Zinc finger) [PMID: 7958847, PMID: 17384421] domain is a versatile protein-protein interaction motif involved in many cellular functions, including transcriptional regulation, cytoskeleton dynamics, ion channel assembly and gating, and targeting proteins for ubiquitination [PMID: 16207353]. (http://www.ebi.ac.uk/interpro/entry/IPR011333).

>ULKT_2018407_Lycopodiella_apressa
EDLERIAESLSPEGIPLCLGCLYDTNSEQKSLARRECIRLFGTLALLHGFLLAPYLSKIISCIIRRLKDPDTIVRDACVD
AVGAIALNVISPEDEDPSADYGNDGSFPSVFEVFFKPLVEALNEQNKHLQVGAAMCLARLIDSSSHPRSSSVQRLY
PRIIKLLKASNFLAKAALLSVVGSLTKADEVEADQFLASIVPHVQASLESSDWAIRRA

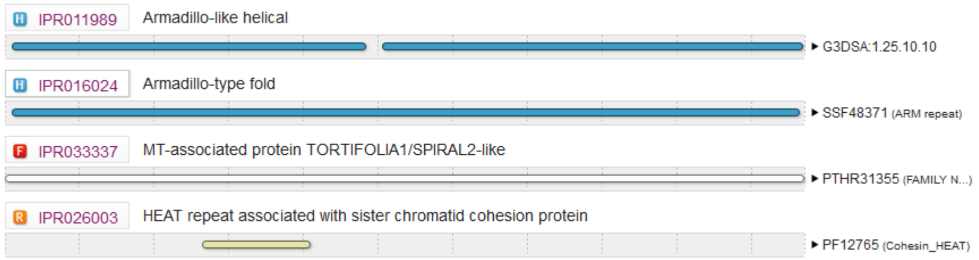


This entry includes a group of plant microtubule-associated proteins, including TORTIFOLIA1/SPIRAL2 and SPIRAL2-like (SP2L) from Arabidopsis. They regulate the orientation of cortical microtubules and the direction of organ growth [PMID: 18577573]. (http://www.ebi.ac.uk/interpro/entry/IPR033337).
This HEAT repeat is found most frequently in sister chromatid cohesion proteins such as Nipped-B [PMID: 15060134]. HEAT repeats are found tandemly repeated in many proteins, and they appear to serve as flexible scaffolding on which other components can assemble. (http://www.ebi.ac.uk/interpro/entry/IPR026003).

## Araucariales

>IZGN_2001467_Dacrydium_balansae.nd
KLTVNDALCCDCRELIYLPTVLNCGHAFCETCVSISEDGTLK**C**QV**C**QSVHPGDRPHI**C**LELNHFLELMFPLEYAER
KKSILVNKQT**C**KQSKEESTS**C**KKKAENTSAEVPANSSEQAAIIHPGVG**C**DYCGIIPIMGKRYKCLDCPESIGFDLCG
KCYEIGSNLPGRFNQKHTAEHSFEV

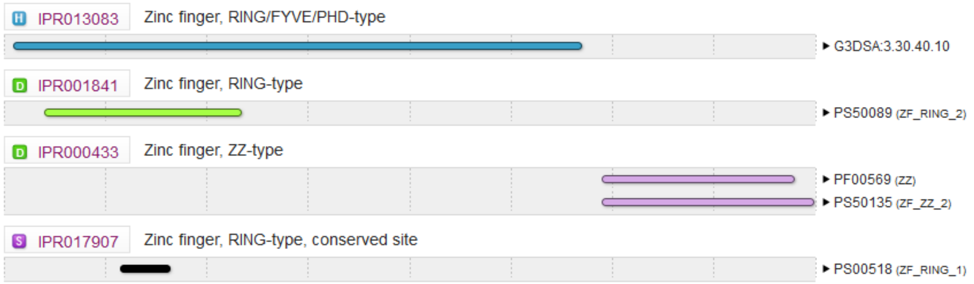


Zinc finger (Znf) domains are relatively small protein motifs which contain multiple finger-like protrusions that make tandem contacts with their target molecule. Some of these domains bind zinc, but many do not; instead binding other metals such as iron, or no metal at all. For example, some family members form salt bridges to stabilise the finger-like folds. They were first identified as a DNA-binding motif in transcription factor TFIIIA from Xenopus laevis (African clawed frog), however they are now recognised to bind DNA, RNA, protein and/or lipid substrates [PMID: 10529348, PMID: 15963892, PMID: 15718139, PMID: 17210253, PMID: 12665246]. (http://www.ebi.ac.uk/interpro/entry/IPR013083).
