# Supplementary material for: The Cell Wall PAC (Proline-Rich, Arabinogalactan Proteins, Conserved Cysteines) Domain-Proteins Are Conserved in the Green Lineage
Source: Int J Mol Sci. 2020 Apr 3;21(7):2488. doi: 10.3390/ijms21072488 (PMC7177597; doi:10.3390/ijms21072488)
Supplement: Supplementary file 1 [file ijms-21-02488-s001.zip › Supplementary files_R1_Nguyen-Kim, San Clemente et al/Nguyen-Kim, San Clemente et al._Supplementary file 4.pdf]

Supplementary file 4. Amino acid sequences of putative PAC domains of ancestors to the green lineage.

When several PAC domain sequences are present in a protein, they are numbered in red (*e.g.* >g11926_1_Chlamydomonas_rheinardtii).

Cys residues are highlighted in red. Pro-rich motifs are in bold light blue. The position of the PAC domain is highlighted in yellow in full-length amino acid sequences.

Chlorophyta; *Chlorophyceae*; Chlamydomonadales; *Volvocaceae*

>Vocar20006048m
CDDCRTQPGSGKRICWTPIGFEEHAGMGSSKKWKKSIRLLRFRCGSAAEALPASGSRGGCQTLGEWLERHGLR
VRTSRLRQREMDAAEVELC

Full length sequence
>Vocar20006048m
MSFPLVVSILSCHLLCLKCEQLQQQPPHEGLEQPPFAQAVFLDGGERQQQQQLQPPPPPPTPQQSQDNQSKVA
EQRAPLQLLMGHYEAQELPDGSPARDYLDWNDELERLPLLPLSPLPELSPPGVPPPLQEHEQPPESFPAAPPPQ
QTTLMWIPFGGDSDSYSDEDEEGDEDEEEEDEEGTGEGTGDEEAKMMRCHARYTGRKLQVTWQLFPVATTDL
QHVFITMAVGGQNTSEAGESFPFGSARQAVDLKIEVGKELSKRSSRCSTNWRPSLEHPRFSQAVHMNTQQHRQ
QQPAQHAATTATAALAAAPVGGDVRTPTHYHHASLAELPDEAAREVAAVVARELDGALPVVCKDHTGLFMLRYM
LVACDCDDCRTQPGSGKRICWTPIGFEEHAGMGSSKKWKKSIRLLRFRCGSAAEALPASGSRGGCQTLGEWLE
RHGLRVRTSRLRQREMDAAEVELCIRVSGLRWTDDD

Chlorophyta; *Chlorophyceae*; Chlamydomonadales; *Chlamydomonadaceae*

>g8431_Chlamydomonas_rheinardtii
CSACVVQTIVPPVSGTPGDIFTFVPELCEEQGAFIADSLNNAAADVGAALATPFATASCSAAWDPAAQPPVLPVLR
VCGAVATAADAALLQAWLESAAAPLDDWLSHTAGGDDC
>Cre12_g549000_Chlamydomonas_rheinardtii
CPICITLNTTDVSLTREVCDNLADYVNSVWVDRSNATLLQPFSCSLATPSQVKICAVAEGKPDTVDFAGIAEDTYEI
QLMLEELGLSC

>Cre12_g506750_Chlamydomonas_rheinardtii
CVVCYNLASAGFSFNNRTCVLFAGFTNGLLTQDWMFQEGLILEPFVCTQATPNFVQICATYASAEVAQEAADYISA
DDFSLVELMWDYMRLRRYLC

Full length sequence
>Cre12.g506750.t1.3
MPVRARFLSALLVGLVGLASAQTQPTFPFCQCVRSTSTSRYEVVDAPAVALKAGYWCVQLRVKEQCDKPKNPCC
NADLYKIELLAQSGCVGSRMTASYGYSAASLVEVPSVPTWEVPDAAPEGAAVLKLVRLGLNTSTADGMYICYKAL
SGSCKKPEQVCLPGATGQCQLSLFNTDSSCCPTETFPLPPPPSPPPPSPPPPSPPPPSPPPPSPPPPSPPPPSPP
PPPSPPPPCVVCYNLASAGFSFNNRTCVLFAGFTNGLLTQDWMFQEGLILEPFVCTQATPNFVQICATYASAEVA
QEAADYISADDFSLVELMWDYMRLRRYLCGGQGSLQFSSESDCINPLLYNSRACALEQTPFPFCECDKTPGTMP
FGVDYYYSEPANSKFCFTVLAVTPNDPSSSCGKITTIKKVEFNIQDSVKRQIKAVVDASGKTLRTAWGSLGDNTFKI
TPLSWTLPQLTAAPQQICIITTGAELSDLCVGGGCTASVFDDSQKCCPTSTSQLEPVVPDDAFRSFP

>Cre10_g449000_Chlamydomonas_rheinardtii
CDICVELTNLDGTLGTPGVPDKQCSALATALTTFFIQGAELAGFSVPAAEFQCVEQSGTRQVACVGGVDQGDVEP
MADHFWQTGAMFDKAVGNMIGLGPVAC
>Cre10_g448977_Chlamydomonas_rheinardtii
CEVCVTVLKGIGDLGSTADNCTLLAELANEHFRNDSEGGPPIPQATNFVCKPNFTPTEIYACETVDPQTADQMMF
KFDGDTDTVYFYYFIPFDINEINTC
>Cre06_g270500_Chlamydomonas_rheinardtii
CHHCGQGGGQWKMPDVCGNPFQDSPPINFTTRFYGFVANYTQGQEVDITINIATNHGGRMAMRVCPLSRGLISH
DCFNNPANQLRRVSSNPQYDAKLYWYVRPNDIDITQRFRLPPGVSC
>Cre06_g273100_Chlamydomonas_rheinardtii
CEHCGQGNGAPPDVCGNPFQGSTGVNFTDPYTWFDGFKATWTQEQEVDITIYLSTNHGGRMAMRLCPRDRFGL
YPTCFNEPANQLRRVSPDPKYNGKVYWYLKPSDAEITQRFRLPPGVSC
>Cre02_g118650_1_Chlamydomonas_rheinardtii
CQLCIQLSITASSTDVFIPYSLSEYECGAFGATIADELAFAVYDLLGPAAPGGFNIVACVPDATDAFGRHTTYVRVC
GQVREPISGSDFATLQQVLETQVGTWQTLPGNGQC
>Cre02_g118650_2_Chlamydomonas_rheinardtii

CQLCLTLQFIGTGTSGAKSFCAALNKAAADTFQPLADAQGIRLLGSGSGGGSVWRASGCGLTDSPGIAAVSVCAD
LLAAQAPALPRVLSQGLLQLFDSLFPPLASGAGSGGGC

>g11926_1_Chlamydomonas_rheinardtii
CEVCLTLTLLPPPGAAAATQGGGLPFALTDPLCTDLESPMLSGLADAARAAGATISIPFARTACSTSYVPTATPMVL
PYLRLCGAFRTASDGLRLQSWLAGPTTGLSAWLLQLTGGEAC
>g11926_2_Chlamydomonas_rheinardtii
CVVCITTTIQPPVSASASTVYNLTDAQCLDLSSTIIDNMQMLAFDTGAVIVTPFTQDACSPSYNATSVPAKYPFARV
CGAFLSAEDGALVADPLKELMKEWVESLTGGAAC
>g11926_3_Chlamydomonas_rheinardtii
CVVCITTTIQPPVSASASTVYSLTDAQCLDLSSTIIDNMQMLAFDTGAVIVTPFAQDACSLSYNATSVPAKYPFARV
CGAFLSADDGALVADPLKELMKEWVESLTGGAAC

Full length sequence
>g11926.t1
MAWRWRAWTFTLLVLFATWPTARGQGASTPSDEYDAALPARSTSSFPWCQCVAYDCGCSPYQLQVLPPTRDGI
NVRSCFRVTYVGCDTTRKCCRGMLAAVDKLALETSAACSERGRVLGVTLNGRVHESWNPYVHGTGAELKIYNLQ
RSNATFAGVLVCVTTAPPCGWLNDLCVHGSGGPGGAGADGTSGGGGGMQNSCRYSLSDTPEATYCPVCAVRL
PPAPPPPSPPGPNAPGRRRARPPPPEPTPPPSPTPSPAPTPCEVCLTLTLLPPPGAAAATQGGGLPFALTDPLCT
DLESPMLSGLADAARAAGATISIPFARTACSTSYVPTATPMVLPYLRLCGAFRTASDGLRLQSWLAGPTTGLSAWL
LQLTGGEACPASPGLSGYVITSLASSPAGGDTCLSAAAVQTCVAAQAPPPPPPPKARMPPPRASAAARAPPLPPL
PDVPQVGGPQCELYLHIQVLPAAASAASASSSLAAGAFGRRRMQQAWGPGVEGEEVVGKGGSAAAGEAAAMA
VTPQAVALRLPGGSGSLSGSGSGSRAASNGWRRQQKDITTGEQAFSFDVASCAELADRIISGFAVQAPLVGSEIT
VPFTQFACAPQQLTLYGAFATAYDGHLLYDWLNGPDGMQAWVHAVTGGDACSQRLSGYTIRSLVTGPGGELAA
SCMSGFAQQQCPSTPPPFLPPLPPESPSSPSPPLPPPDSPPPPSPWPPPDTPPPSPPPPPPPDAPPPPPPNSPP
QSTPPPPPDSPPPSPPPPPPTPPVAPEPPSPQPPGLPSPSPPQIPGPQVPSPPLPSPLPPSPPPSPAPPSPQPPL
PPPSPSPPPCVVCITTTIQPPVSASASTVYNLTDAQCLDLSSTIIDNMQMLAFDTGAVIVTPFTQDACSPSYNATSV
PAKYPFARVCGAFLSAEDGALVADPLKELMKEWVESLTGGAACSSASLSGHLISSKATDPSGAASCLDASFRQAC
SIRRPPSPRPPQPRPPSPAPPSPAPPSPGPPSPQPPSPEPPSPEPPSPEPPTPVPPSPAPPSPAPPSPAPPSPAP
PLPPPPSPVPPSPAPPSPVPPSPMPPSPAPPSPTPPSPTPPSPVPPSPQPPSPAPPSPAPPSPTPPSPAPPTPAP
PSPIPPSPPPPAPPPPCITCVSVFVLPDSLISGVPFRPNASLCSSMAAMVSAGFTAAAADAGAVVVQPFAVTSCVR
GGGPLAGVLTPLMKICGGLLSADDGAKLQLRIPAATAQPSPPVSATTFATAAFATAAFPTAAASATTQPATTEPAT
PEPVATVSTATQPSTALAVAALAAAAGPSASFTAATLAAAAPSPAAPTAATVAAATGSASTKPFAFDDSECGKLAK
RIDDGFKQQAALLGANIQVPFRLDHCTPTYLKLMGTFGSARDGEMLQEWLNGPLGMLDWVKSVTGECPDRLSGY
AVRSAVQPPNNDTNSCLVGDFKLECFYQPPPPQPPLPPPSPPPPCVVCITTTIQPPVSASASTVYSLTDAQCLDLS
STIIDNMQMLAFDTGAVIVTPFAQDACSLSYNATSVPAKYPFARVCGAFLSADDGALVADPLKELMKEWVESLTG
GAACSSASLSGHLISSKTPLSPAPFAFTPASCADLAAVITDDVSQQAADMGSVMLAPFALTECSDNSLRLCGTFQS
AADGAALGPWLEDQSQFWLEALGGGDCSAELAGYSMVFEAVAGPGGGGVCLQVNAPPLPRIPCEVLVHILLQPP
DGLPAVAEAITDGVGSATSTSRHRERLLLQQLTVTATAADASGSAAFSFDAASCAALSTRIATGFAVQAPLVGSDIT
VPFTQFACAPQQLVLYGSFATAYDGFLLYDWLNGPDGMQAWVHSVTGGDACSPELQGYLIRSVVTGPSGELAP
SCMAGGFKQQCEGGPALPPPPPPPPSPPPPLLPPSPSPPSPGPPPRQATPGLRQPSMPPPPPPSPSPPPPPPS
RPDLVQTHECVASTNDVPYSVGPLYVRQSLDQFGAPAVAMCTRVSGRQACRQAAFCCDMDFAKIEVPINPACRN
ELRRIAVNRRLKDWSWGSYPSGLLTVKFESLITDLPDGPDGAELCWLVRPGGVCADPSVFCLQRSCQVNIFSTNN
KCCPSAFI

>g11927_1_Chlamydomonas_rheinardtii
CEVCVTLTIIPPVLSDGTSVFTFDNDSCADSATTFIASMEFIASDAGSLIVAPFVLTTCSPAYNVSESPIVYPYLRVCG
AFLSASDGAMIESALQDFMQLWIQDLVGGDPEAC
>g11927_2_Chlamydomonas_rheinardtii
CEVCITLTIRPPDILPGPLFTFDRDTCTSVSDMMIINLANIAADVGALLITPFTLDICSGDFSATESPPVYPYLRVCGAL
LSAEQGARMQPGVLAAMQIWIMSLAGGPAEDC

Streptophyta; *Streptophytina*; *Coleochaetophyceae*; Coleochaetales; *Coleochaetaceae*
>GBSL01052221_Coleochaete_orbicularis
GVVYCDCCRTGKPNTNETNMQGAEVEYTCLTYTGGESSQTLRTDSNGQYLSAPLDSALVTHCTVMLKSSPDSVC
AELGPCGGGPIFETPIPANASVSVRIPDFC

Full length sequence
>GBSL01052221.1_6 TSA: Coleochaete orbicularis comp36682_c0_seq1 transcribed RNA sequence
VPPSLAPPPATVLSSSGILRARYNYRGGKGVTFRLLGVVYCDCCRTGKPNTNETNMQGAEVEYTCLTYTGGESS
QTLRTDSNGQYLSAPLDSALVTHCTVMLKSSPDSVCAELGPCGGGPIFETPIPANASVSVRIPDFCFQPSPEGMQ
DNYSDKCTPPGPEPSTAPNSDETSTSVEASPTDDSPPADGDHEGPSPQYTLPPSELSPYLAPTPAPSPAPEER
