# Supplementary material for: The Cell Wall PAC (Proline-Rich, Arabinogalactan Proteins, Conserved Cysteines) Domain-Proteins Are Conserved in the Green Lineage
Source: Int J Mol Sci. 2020 Apr 3;21(7):2488. doi: 10.3390/ijms21072488 (PMC7177597; doi:10.3390/ijms21072488)
Supplement: Supplementary file 1 [file ijms-21-02488-s001.zip › Supplementary files_R1_Nguyen-Kim, San Clemente et al/Nguyen-Kim, San Clemente et al._Supplementary file 5.pdf]

Supplementary file 5. Amino acid sequences of putative PAC domains with only five Cys residues, more than six Cys residues, or no Gly upstream Cys1.

In the protein name, “.nd” means that the type of protein could not be determined because the amino acid sequence of the protein is not complete; “.1”, “.2”, “.3” or “.4” refers to the type of protein (see Figure 2).

Originally, the accession numbers originating from the onekp database were of the following type: #onekp:JVSZ_scaffold_2013751 Equisetum_hymale. They have been simplified for more convenience, as follows: >JVSZ_2013751_Equisetum_hymale.

Five Cys residues

>LOC_Os07g40100.1
GEVY**C**DT**C**RAGFVTNVTTAIQAARVRLE**C**RHYMSASGSVERSAEGTTDATGHYRMELVEV
DNRGAELV**C**AVALLSSPVTE**C**HEMEVGRDRAPVTLVQDVGLATMVRRANPLGFLQTMLAR
YADTDTRYVDTKMWHL
>Aco007813.2
GSVT**C**LD**C**TPHHNLSGVVVAVK**C**AHMKKLLPAVTNNEGYFIVSIPATTPKSSPTLTPQ**C**FAR
LLGGREQLYAFERSMAASIVAVGRGSNGYDLASPLAYLSK**C**

Seven Cys residues

>JVSZ_2013751_Equisetum_hymale.**2** (incomplete sequence)
GSVY**C**DQ**C**LQGHRTLWSTPLRGASVALE**C**MNSTAAGQKKAETDLLGMYAIRLEGSLDMQG
CIARLVSSPKGSSG**C**NLAGVASRPLKLTWKLFGVSYYTSDALS**C**
>IZGN_2005470_Dacrydium_balansae.2
GSVF**C**DK**C**SQHSLHLPTTYFIPDATVAVE**C**SLNRKARASIRVEGRTDQSGEFRIELPSSIYNT
YSG**C**SVEVINSPDGL**C**NVPSVNKLAFTMNGKHDSVY**C**AGILSYRPQVLPSA**C**
>IZGN_2005471 Dacrydium_balansae.2
GSVF**C**DT**C**SHHSLHSPTTYFITDATVAVE**C**SLNRKARASIRVEGRTDQSGEFRIELPSSIYN
TYSG**C**SVEVINSPDGL**C**NVPSVNKLAFTMNGKHDSVY**C**AGILSYRPQVLPSA**C**
>Spipo4G0058500.2
GTVF**C**DI**C**ANNSFSKHSYFLRGAKVRVE**C**SFKAAAAAREEITISVERTTDRFGVYRVDVPPV
DDFQ**C**REGREIESF**C**RANLVRSSSSM**C**NVPGLSISSEHVGVG**C**
>Spipo1G0064500.2
GTVF**C**DQ**C**LDGQRDLFDYPLYGARVAVV**C**TGGGDGQMALYGEATTNWFGVY**C**VQFDGG
PDLSS**C**SAQVVSGTQW**C**GGAAGPPGGLHLLFGLFGTAMYTADALLALPSKPASF**C**
>P.equestris_XP_020598742.1
GRVF**C**DT**C**RAGFETPATTYIEGAKVKVE**C**KAGDKI**C**SYDGVTDNSGTFNILVEDEHEHEI**C**E
AMLVSSPDTE**C**KTEVPGREKSRIVLSHNNGISSDTRFANALGFQKDTPLAF**C**
>GSMUA_Achr1T21560.1
GRVF**C**DT**C**RAGFETPVSTYTKGAKVRIE**C**RSKATGAKT**C**NFEGTTDHTGTYNILVADEHEH
EI**C**ESMLVSSPESR**C**KTPLQGRERARVFLSHNNGIASDTRYANSLGFQTDTPLSV**C**
>GSMUA_Achr11T13000.1
GRVF**C**DT**C**RAGFETPASTYIRGAKVSVE**C**RSRITGAKT**C**SFDGTTDHTGTYNILIADEHDDH
EI**C**ESVLVSSPESG**C**KTVLRGRERARVFLSRNNGIASDTRFANSLGFTKDTPLSI**C**
>GSMUA_AchrUnT14160.1
GRVF**C**DT**C**RAGFETPVSTYIQGATVRVE**C**RSEFTGAKT**C**SFEGTTDHTGTYNILVEGEHDD
HEI**C**ESLLVSSPESG**C**KTALQGRERAPVFLSHNNGIASDTRFANSLGFLKDTSLPV**C**

>GSMUA_Achr8T21210.1
GMVYCQKCKYVGSWNLDGARPLPSAKVSIICKDHKHRVIFYDSAETDRNGYFYKLLDGAHL
RSATFDPVTACTARLLASPDASCNRLTNVNYGIQGAKLRDESKTNSGEHCETELYAAGPLAF
KPVHC
>Oropetium_21061A.1
GRVWCDTCRAGFETPASTYIAGAKVRVDCKSKTTGEKTCSFEGHTDRTGTYNILVADEHEH
ELCESTLVSSPDTSCAKAVAGRERAPVFLTNNNGVASNVRMANALGFKKDVALPEC
>Oropetium_08744A.1
GRVYCDTCRAGFETNVTEYMKGAKVRLECRHFGTGAVERAVDGVTDETGTYRIELEGNHV
EDICEVTLVQSPRADCAEVEQHRDRARVVLTTDGGICDALRLANPLGYLKDAPLPVC
>Oropetium_24235A.2
GSVYCDACSNNTFSKHSFFLKGAKVLIQCNFKVNSTASEELSLAAERTTDQNGVYKLDVPTV
AGFACREGHELRSACRATLVRSSSAACNVPGLTRGSTQHVALRSRATNAC
>Oropetium_26560A.2
GTVFCDACSSSSFSNHSYFLPGVKVRLDCMVKVNSNSREEIKITAEKVTNSYGAYRLDIPAID
GFECAATAAEALCRAAVLDNPSELCNVPAVTTTVRHVSFPTPNAASGC
>Bradi2g03930.1
GLILCQPCAHRNSQCLDAATPIPNAQVTVTCRDPKNRVKTSRVAKADAQGYFLAEIGVADGK
QEFYEGDPSKACFVRLLSSPDKGCNDLTNVRYGIEGAELRDEGKRWVADGVENVVYAAGP
LAFRPRSC
>Bradi2g31950.1
GRVYCDTCRAGFETNASHSIPGAVVQMECRHFETNELHHKAEATTDAQGWYKMEVGEDH
QEEICEVALLRSPEKDCAEIEKSRDRCRVPLTRNNGIKQSGVRYANPIAFFRKEPLANC
>Bradi3g00370.2
GMVFCDQCKDGARGLFDYPLYGARVAIQCGGGETPLTVRECNTNWFGGFSVRMEGSPDM
NRCTARLIQGTPHCPGGGSTAAPQQRDLTLAFRMLGLALYTVQPLLSHPDHPMDFC
>Bradi2g48680.3
GVIYCKPCRSRRYSRDMDASPLQGATAQLVCYGREVVNVTGTVTDERGYFLVMFYDLGNF
HPRNCKLYLGTSPTTLCDNPVYPPNKWIGLSLVRETRTTPPEGLQGIYCPTSVLFYGPTAGQ
KC
>LOC_Os04g32680.1
GRVYCDTCRAGFETNVTEYIKGAKVRLECKHFGTDKVERAIDGVTDETGTYKIELKDSHEED
ICEVVLVHSPLANCSEIEAERDRARVLLTRNVGICDNLRLANPLGYLKDIPLPIC
>LOC_Os10g13914.1
GQVACELCLCPGSDLLTFELPGAKVEVACETEGPNTQANSVFTATDEFGNFTFHLPSRLHA
TPSLENACVVKVLQLPPDSACGLRHRPAASYRIRPSSSLSSSADGFRAYTAGVIRLQHGGTP
SGEC
>LOC_Os10g39890.1
GSVLCQDCTKSWNAYAYNAKPIPGSMVGITCLDKETGRTVYHGTDKTDDKGMFNIEVPYTV
GSAHLHPSACLVRLASSGDHGCAVFTNFNGGKTGERPCRPSHVYPGRVTYSAGPFYFTLS
QC
>LOC_Os10g22590.1
GRVWCDTCLAGFETPASTYIAGAKVKVECRSKSTGAKTCSFEGQTDHTGTYNIPVNDEHEH
ELCESVLVSSPDAKCGKIVAGRERAPVFLTNNNGVTSNVRLANALGFQKDAPLAAC
>LOC_Os09g32988.2
GSVYCDACSNNTFSKHSFFLKGARVLIQCSFKVNSTMAEELSLEAERTTDQNGVYKLDVPA
AGGFDCREGHDLRSACRATLVRSSSAACNVPGLRGSTQHIALRSRATNAC
>LOC_Os02g01190.2
GMVFCDQCKDGARGLFDYPLYGARVAIECGGGESPVTVRECNTNWFGGFSVRMEGTPEM
NRCTARVVQATGHCGAAIPTAPRDLTLAFRMLGLALYTVPPLLSQPLRPMDFC
>LOC_Os01g52660.3

GVVYCKTCKSRGYSSDMDASPLPGATAQLVCYGKKVVNVTGTVTDANGYFLVMFYDLRNF
NPRTCKVFLGSSPTSLCDKPVYPPNKWIGLSLLKETRTVPPVGLQAIYCPTSVLFYGPANAG
QC
>Sobic_009G082900.1
GRIYCDNCRAGFETNISHAIQGATVEMECRHFESQQIHDKAQATTDAGGWYKMEIAGDHQD
EICDVRLLKSPEADCAEIEVNRDRCRVPLTGNDGIKQSGVRYANPIAFFRKDPLPNC
>Sobic_003G059000.3
GVVLCQSCEKKGSQSLDGATPLPRANVTVTCRDRKNCVMAYRQRAADDNGYFHAEFGVQ
RADYYLDKDPLEACFVRLLSSPDAKCNVVTDINGGMLGAPVRGEGKQWTDHRGFKNVVYA
AGPLAFTPEKC
>Zm00008a036634.1
GTVFCDACSTSSFSKNSYFLPGVRVRLDCMIKVNSNSKEEIKITAEKVTNSDGAYQLDIPAID
GFQCAAPGATAGESFCRAAVLDNPSALCGVPAVTTTVGHISLPSQDPSAC
>Zm00008a004591.1
GQLTCELCLRPGSPLLALEMPGAKVAVTCDCESHQHPSDEPDSHALLAATTDESGNFTIDLP
ARLHATPDLEKACAVKVLRLPAGSSSCRLRHRAADAYRLRLASAEDGARAYTAGVIRLRDG
DTPSDRC
>Zm00008a025512.1
GRVYCDNCRAGFETNVSHAIEGATVEMECRHFESQEVHDKAEATTDAGGWYRMEISGDH
QDEICEVLLLKSPEADCAEIERTRDRCRVPLTRNDGIRQSGIRYANPIAFFRKEPLSNC
>Aco000299.1
GRVVCDVCADAAIGPEDHVLEDCLMLASENIFEYSPLRTGAEVAVLCITKSGEVLNYQAFTD
SKGVYTVAETMPESDRWESCLARPISSFHDRCAKRGDSHSGVKFTYNRPSGHSHTVKPFL
YKPTSVPVYC
>Aco003633.2
GTVFCDACSDNTFSKHCYFLQGVKVRVDCMFKVNSTSKEEISITVDRVTDRFGVYKLDIPPV
DGFECRVGREIESFCKASLLESPSTLCNVPELSSSIEHVSLKSPGGTMC
>Aco006667.2
GSVYCDPCSNNTFSKHSFFLKGARVQIQCRFRVNSTSREEILIEAERTTDKFGIYKLDIPPVD
GFECREGREIRSACRANLISSSSSSCNVPGLQGSTQHLAIKPKQANIC
>Aqcoe6G061600.1
GKVFCDTCRLGFETPATTYITGAKVRVVCKTRDSLQPVFSCDAFTDSTGSYSVTVAYDHED
QICESVLISSPQKNCAAIMPGRERANVILTNNNGIISYNRFANNLGFVRDEPLAAC
>Aqcoe5G346000.2
GFVYCDICSNNTFTRHSYFLPGVKVHINCKFNAISATTTEQISFSVNRTTDKYGIYKMEIPSVE
GIECAEGVSVQSSCEARLITSSSSSCNVPGLTTTSDQSAFKLKQDNVC
>Aqcoe1G261600.3
GVVYCKSCKYSAFDTLLGAKPLSDAVVKLECNNTKKPITTYGKTDKNGYFFFQAPKKVSTHG
FHKCRAFLVSSPSSFSCTKPTDMNSGWKKGALLIAANQTFPTPIALFSVGPFCFAPTKC
>Aqcoe3G160500.1
GTVYCDMCSSNTFSRHSFYLSGVDVHIDCKFKASIPTTSELISFSVNRTTDKYGVYKLDIPSV
GVNCAEGHATELSCRAITIGSSSSLCNIPGLSVASEQVKIKSRHNHC
>Lsat_1_v5_gn_5_42161.1
GRVYCDTCRCGFETSATTYLAGAKVRVECRDRNSMNLRYTMDAVTDATGTYHIEVATDHG
DQKCECTLVGSPDPGCGKPNIGRHRATVILTSNNGMNYGARYANAMGFLKDTALAGC
>Lsat_1_v5_gn_6_28201.1
GKVLCQDCTEGWNEWVSGQKPIEGCTVSVTCMDNRKRIIYYGRDQTDKAGEFVVTINKYIH
GKQVKPESCFTRLVSSPDPVCNIPTDFAGGKSGVKLGRPTMVYRDTIKHVLGPFYYTTPMC
>Potri.006G119100.1
GKVMCQDCTKGYNDWINGDRPIKGSKVCLTCTDDRGRVIHYDSDVTDERGEFDMIVSKYIN
GKQLKEKKCSVRLVSSPDPSCNILTDFAGGKSGVKLKRPTSVYRDTVKYMLTPFYFTSPMC
>Eucgr.K02418.1

GRVLCQDCTKGYDEWVKGGSPIKGAKVSLTCMDQRYRVIFYGSDDTDGTGQFNMIVDKYIN
GKQLNEKLCLVRLVSSPDPTCNVATDFAGGCRGVKLSRPRSVYREVIKYAVGPLYYTSPMC
>Eucgr.D00613.3
GVVYCKSCKYSGVETLLGATPVLRSCRFDDEVSFFAGAVVKLECNNTKYRPTVQTAKTDKN
GYFFLKAPKTITTYAFHKCMASLASSPVPSCQKPSALHGGTVGAVLRPEKPVMIDKLPYTVF
TVGPFAFEPQC
>AYMT_2115347_Eucalyptus_leucoxylon.nd
GMVYCDICFNNTFSRHSYFLPGAEVKIDCKFEAVSPKTTEQISFSVKRTTNRYGVYKLEIPAV
DGIECAKDSAIVSFCEASLMGISSDSCNVPGYRTTTDQIAFKSKQANLC
>Gorai.010G248800.1
GTVLCEACHQRRRRDPQPELRSWPISGALVSVKCETPCKTKSGTTPATTDEYGDFMIDLPS
HLHGVADLQKICTVKVIGIPKESMCRPAFVKKHEHLRLSSVGNGIRTYTAGRIRFQDIMSKPS
KSC

Eight Cys residues

>Zosma169g00070.2
GTVFCDICSKNTFTKHSYFLPGTKVEVQCNFKVINSTSKEEISITVDRKTDRYGIYRVDVPPV
DGFECRQGREIQSFCRARLIQSSSSLCNVPGLSTSVEHLAMKFSQSNLCIYNLNALNYKPSK
KNLALC
>P.equestris_XP_020589188.2
GTVFCDACSENTFSKHSYFLQGATVQISCKFSTETTSGEEISINTKRTTDKFGIYKLEIPPVDG
FQCSEGRALQSFCKASLVQSPSPLCNIPSLSSSKQHLAFKAGGFNSCIYDLNALSYKPAQRD
SSFC
>GSMUA_Achr9T29260.2
GTVFCDACANNNFSQHSYFLEGVKVRVQCVLRVNETSAGEMRVAVDRTTDRFGVYKLDIP
PVDGFECREGRGVDSFCRASLAESPSSLCDVPGLSSSTEHVAVRGGEGKLCLYNLNALNY
RPSKKDADLC
>Bradi3g32620.1
GTVLCQDCTKNWNAYAYNAKPIPGSGVAVTCLEKGKVAFYGKDETNKEGVFSVEVPYEPA
GSGAGCRLDPSECLVRLVASEDKGCSVLTNFNGGRSGQRPFRPSKVCPTEVVYNVGPYYA
TLPQC
>Aco011770.1
GKVLCQDCSKGWNQWAHGATPIIGSKVGVTCMGPQARVLLHKSDACDRRGEFDVVVDKY
VNGVEVNPEECTVRLVSSGCQNCTVMTNFNGGKDGVRLSRPSTVYPGRIAYKVGPFYFTT
PKC
>Lsat_1_v5_gn_0_3380.1
GVVYCDACSNNSFSTHSYFLPGAEVRIDCKFKAASPRTTEQISFSVNRTTNKHGVYKLEIPS
VDGINCAKEEVQVLNTCRASLIRSTSPACNVPAFITTSNQFSVKSKQANLCIYSLYALSFRPS
KKDLAIC
>Lsat_1_v5_gn_4_186081.1
GMVYCDVCSNNSFNNNHSYFLSGAEVRIDCKFNAVSPRTAEQISVSVNKTTNRYGVYRLDI
PSVDGIYCASESAVMNTCRASLIASSSPQCNVPGFITTSDEFSLKSQHANLCIYSMFALSFRP
FKKDLTIC
>Lsat_1_v5_gn_4_2141.2
GSVFCDVCYSNLFTRYSYFLPGADVHVQCKFQGNAPSTKEQISFSVNRTTNRYGVYRLDIP
SVEGVDCVEGPAIQSLCEATLIKSSVPGCNVPGVSTTTKEITVKSKSTNVCVYSVSALSYRPF
QINSTLC
>GSVIVT01033047001.1
GIVYCDICSNNTFSRNSYFLPGVEVNIECKFQAISPRTTEQISFSVTRTTDKFGVYKLEIPSVE
GIECASDSAIESSCQASLMWSSSSSCNVPGHKTTTDEIAIKSKQANVCIYSLNALNYRPSERD
TALC

>AT3G26960.1
GFVYCDVCSKNSFSKHSYFMSGVEVRIVCRFKAASSTTTETITFSANRTTNEFGLYKVAISSL
DCADVDSLASSCQASLIGRKNFSDSSCNIPGYRTTTDQVLFKSQQSNSCVYGFNALNFRPF
KRDLALC

Nine Cys residues
>GSMUA_Achr9T01210.1
GTVFCDACANNVFSEHSYFLAGVRVRVQCMLRVTNSTSREEMSITVDRTTDKFGVYKLDIP
PVEGFECREGVVMDSCCRASLLGSPSSLCDVPGLNSSTGHVAVRGGEGKRCLYNLNALNY
RPSKKDANLC

No Gly upstream Cys1
>AmTr_v1.0_019.68.1
DQISCLDCTKHQAHGVGLVIKCDSEKPILAVTDRTGSFKAEITTSKTSAPLEDTCYASLLGPR
NKLCSFKNNQASKFVKSAKYHKDPSSSYYALFTPLSFSSSDC

>Gorai.002G165500.1.
TLLDCYLCPKSFKGLQNVFYSARARERERELLYPGACGGGGWGWISQGIASYGTGHGTRE
TCALHTARLSCDTLVDFWSALGEETRESLLRMKEEDFIERLIYRC
