# Supplementary material for: The Cell Wall PAC (Proline-Rich, Arabinogalactan Proteins, Conserved Cysteines) Domain-Proteins Are Conserved in the Green Lineage
Source: Int J Mol Sci. 2020 Apr 3;21(7):2488. doi: 10.3390/ijms21072488 (PMC7177597; doi:10.3390/ijms21072488)
Supplement: Supplementary file 1 [file ijms-21-02488-s001.zip › Supplementary files_R1_Nguyen-Kim, San Clemente et al/Nguyen-Kim, San Clemente et al._Supplementary file 6.pdf]

Supplementary file 6. Amino acid sequences of putative PAC domains with six Cys residues, but predicted α-helices.

In the protein name, “.nd” means that the type of protein could not be determined because the amino acid sequence of the protein is not complete; “.1”, “.2”, “.3”, “.4” refers to the type of protein (see Figure 2).

Originally, the accession numbers originating from the onekp database were of the following type: #onekp:ILBQ_scaffold_2006312 Conocephalum_conicum. They have been simplified for more convenience, as follows: ILBQ_2006312_Conocephalum_conicum.

Secondary structures have been predicted using SABLE (http://sable.cchmc.org/) and NetSurfP (http://www.cbs.dtu.dk/services/NetSurfP/). Examples of SABLE predictions are given below.

>Mapoly0139s0006.2
GWVFCASCMNVNLPLLGIEAVLLCPHPPPTAGGGGGSAGSAMYEVASTVTSKDGAFHLTA
NASYASIMNQCAVIIAKSSVSNCDLPPPTGTSIPVSTSSTPSEAMSAPDVLHYSVGSFSFRPA
SC

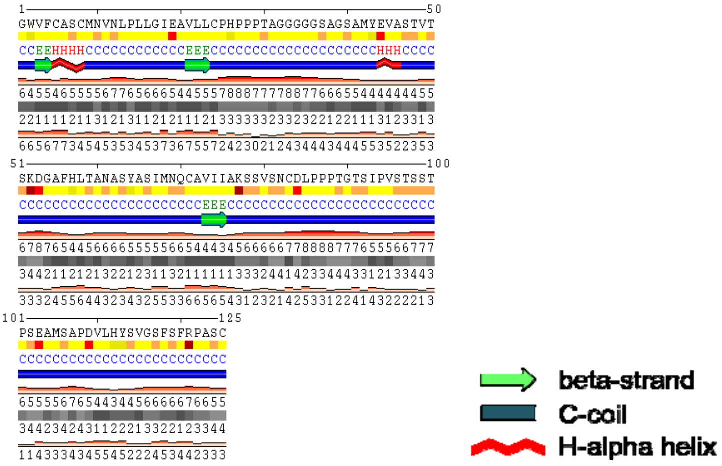

>ILBQ_2006312_Conocephalum_conicum.nd
GVWMCDWCQLGTQAARNDRGSDPAASAAACTVPYVSGDADALEFLLKAALNVNEAGVDD
VAAAGQCSSVDGGSFCSSSIESVKGDEVRLGGGGHSQDASWGSNTERMSEGDEEATTTT
TYRC

>CHJJ_2011674_Lejeuneaceae_sp.nd
GCGSCEACEKQKSSSQKENGGRRNSHCVDDLVEQVMAFTRSSKRRIYQVASLSGSCLGP
SKELPVPCMELFRPNSKWIPSAQVPDLDFKTLQDVVRVNSIDDILNHPRYFMC

>YFGP_2007785_Pallavicinia_lyellii.nd
GCPKCSDCQAVLARWRPEEGCKPVVDAAPVFYPTEEEFKDTLKYIAKIRSAAEPFGICRVVP
PPGWQPPCPLREDTAKLHVMKFPTRVQEIHKLQVRQPVKKC

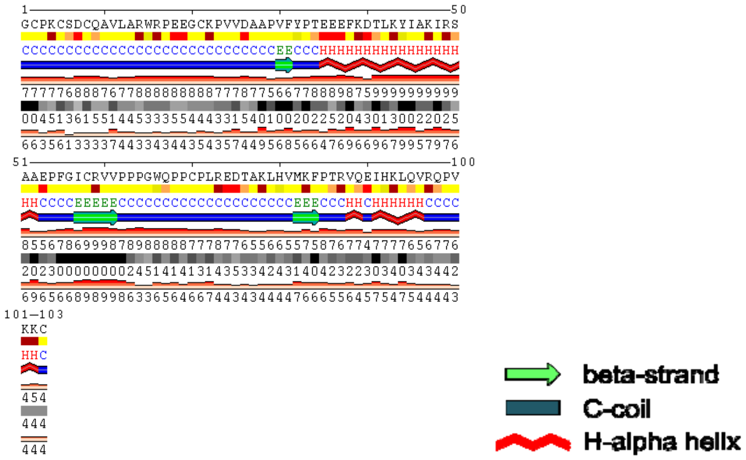

>HVBQ_2020253_Tetraphis_pellucida.nd
GGWLCDWCRLSKQWQPKKDFKGCKPHPVIVKPRSIALDFLVQVALSPTELGDLDVTENFNT
LNMDCGSYTSSSVECDKLGPDAGSQDLGVGERERSSSFENKTVTSDEEGSLGSTRIVKDG
MKKKPHLLRC
>UCRN_2050882_Megaceros_tosanus.nd
GVYTCPFCPNKKRESTGTSNSSIMHKAKVRISAVGCRLEDEMKSAAEVAAQRIVTLQQAVP
KRLNPTHKIVYPVGVLCNLGRKGGDRTLETPGCAELKEKFASFRPKHVKTFGHWGSLGLAV
FTFRRGMEGLDDC

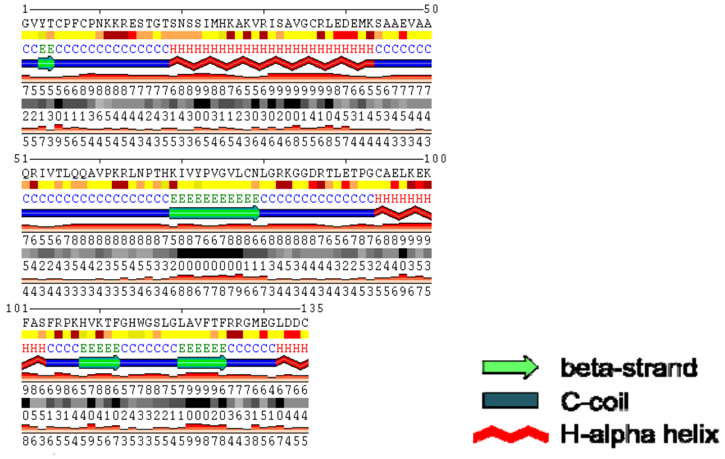

>JVSZ_2014214_Equisetum_hymale.nd
GSECCFQCALLYTTMYNERRIRVMTLSLPCTAILNSVFRSADLDSQFTYFLKQAAQQIPNTPL
PQVKEQVVTSCVNILYAYRKYCAAASSSGQLILPETLKILPLYTLAFTKSMSLRPDVRIDDRC

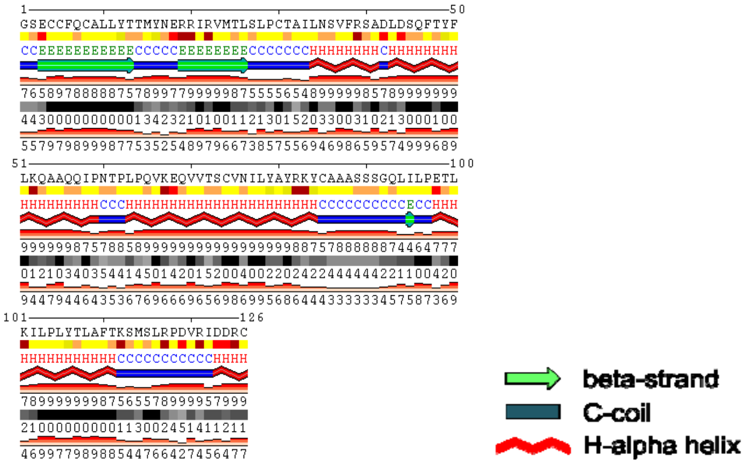

>ALVQ_2020591_Tmesipteris_parva.nd
GLDYCFECVGNVDVMRAAFQSCHDGWGKTILVGIEASNKQLVFNPMQFFDGREIKGCTFG
DFKGRTQIPNLVDTCLTKPDQLDLNAFVTHELPFSRINESFQMLKEGQSLRC
>ALVQ_2017464_Tmesipteris_parva.nd
GCPSCSTCQKVLARWRPEAACLPVLDDAPVFFPLEEEFSDTMEYIASISAEVKQYGICRIVPP
QSWRPPCPLKDFEKWKTLKFPTRLQQIHKLQVREPQHKRYPQSKKRRTGRPAERGLSWQ
RSLFSSEC
>JVSZ_2131256_Equisetum_hymale.nd
GVDYCFECAGNSNLMYAAFESCHDGWGQAILVGVDDAAKKIIFGCMQFFDGREIKGCTFGG
YKGKTQIPGLVQMYLNKELNVERFITHKLPFERIDEAVELLEKGKSLRC
>CAPN_2040128_Equisetum_diffusum.nd
GVDYCFECAGNTNLMHAAFESCHDGWGQAIFVGLSDAAKKVDFSCIEFFDGREMKGCTFG
GFKGKTQISGLVQRYLNKELNVDGFITHKLPFERINEAVELLEKGQSLRC
>2118953_Equisetum_hymale.nd
GCPKCSVCQKVNARWRPEAACRPSIEEAPVYFPLEEEFKDPLEYISSIRAEAEQYGICRIVPP
QSWRPPCPLRHSEKWETLTFSTRVQQVHKLQVREVC
>ENQF_2016454_Lycopodium_annotinum.nd
GADKCKLCKLTSDHVHAHRLVLSSQCEFFQALFRSGMRDSAEQAIRVHRRKNVLYYLYCGE
LLILSEVEGDCAWSHLGTQRQLTYLRQLVELAELAEEWLLEDLQQQC
>ULKT_2018407_Lycopodiella_apressa.nd
GIPLCLGCLYDTNSEQKSLARRECIRLFGTLALLHGFLLAPYLSKIISCIIRRLKDPDTIVRDACV
DAVGAIALNVISPEDEDPSADYGNDGSFPSVFEVFFKPLVEALNEQNKHLQVGAAMC
>IZGN_2001467_Dacrydium_balansae_nd
GTLKCQVCQSVHPGDRPHICLELNHFLELMFPLEYAERKKSILVNKQTCKQSKEESTSCKKK
AENTSAEVPANSSEQAAIIHPGVGC
>Zosma236g00130.2
GVSECSDCGNDDSFKGIQVAVVCKSEDKSTYDLVGVGESSKDGKFSVQLPASLLKKDGTQL
TRQCFAQLHSKSKNAPCPIENHPDSLMLSLASTFKSEGEAALHVFATATGKLAFSRSVC
>Zosma16g01740.2
GKAECPDCGSHNLNGIRVRVMCRSRTSHRLYRSRVHGNRELDLNGKFTLQLPATLLNKDG
TGLIRKCYAQLYSTSKKAQCPTEKNNREMFRLVLTSKFKSVHVFGIPTGKFNFSRNVC
>Spipo1G0061700.2
GAGECSDCASKNIDSAVAFNGLNVAVYCMGGKGQFQIKGVGKLDKDGKFSVKLPSDTVKE
EGGLKEQCFAQLHGGSQNTPCPSAGGSFDAAKLVPESVEGGEHTFVTAGTLSFSSETC
>Spipo6G0013300.2
GAGECSDCAIKNIDAAVAFNGLNVAVYCVAGKGQFHIRGVAKLDKNGQFSVRLPSDIVKENG
GLKEQCFAQLHGGSKNTPCPSAGGSFDAAKLVAKSVEGATHTFVTAGKLRFSSETC
>Lus10026281.2
GFGECADCAKSQLHPSQIFSGLHVTIDCKPHSGEFKTIGSAKLDELGKFSVSLPNGVVAEDG
QKLKQECYAQLHSAADGAAPCAAHGGLESTKVVFNSQKKTFELSGGKIKFSPEVC

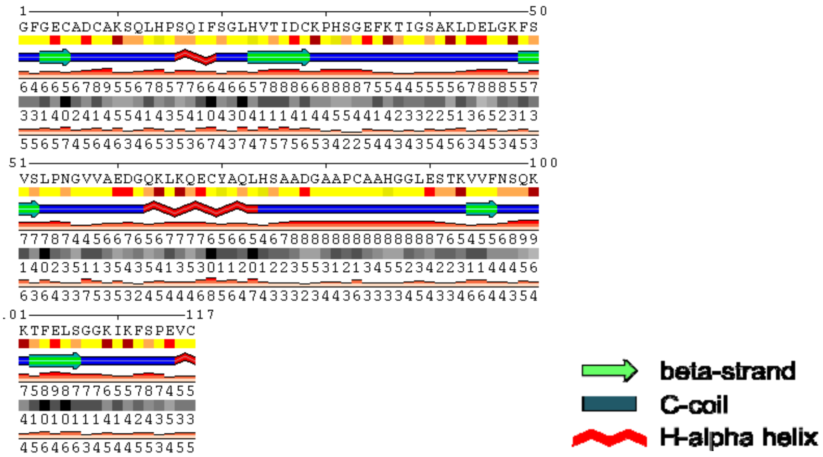
1
50
GFGECADCAKSQLHPSQIFSGLHVTIDCKPHSGEFKTIGSAKLDELGKFS
64665678955678577664665788866888875544555567888557
33140241455414354104304111414455441423322513652313
55657456463465353674374757565342254434354563453453
51
100
VSLPNGVVAEDGQKLKQECYAQLHSAADGAAPCAAHGGLESTKVVFNSQK
77787445667656777765665467888888888888887654556899
14023511354354135301120122355312134552342231144456
63643375353245444685647433323443633334543346644354
.01
117
KTFELSGGKIKFSPEVC
75898777655787455
41010111414243533
45646634544553455
beta-strand
C-coil
H-alpha helix
