# Supplementary material for: The Cell Wall PAC (Proline-Rich, Arabinogalactan Proteins, Conserved Cysteines) Domain-Proteins Are Conserved in the Green Lineage
Source: Int J Mol Sci. 2020 Apr 3;21(7):2488. doi: 10.3390/ijms21072488 (PMC7177597; doi:10.3390/ijms21072488)
Supplement: Supplementary file 1 [file ijms-21-02488-s001.zip › Supplementary files_R1_Nguyen-Kim, San Clemente et al/Nguyen-Kim, San Clemente et al._Supplementary file 7.pdf]

Supplementary file 7. Amino acid sequences of the PAC and W-W domains of type 2-PDPs including a C-terminal W-W domain

Forty proteins are listed. Only those with complete W-W domain have been retained.

In the protein name, “.nd” means that the type of protein could not be determined because the amino acid sequence of the protein is not complete; “.1”, “.2”, “.3” or “.4” refers to the type of protein (see Figure 2).

Originally, the accession numbers originating from the onekp database were of the following type: #onekp:RTMU_scaffold_2181156 Calypogeia_fissa. They have been simplified for more convenience, as follows: RTMU_2181156_Calypogeia_fissa.

Amino acid sequences of PAC domains

>Mapoly0014s0128.2
GNVFCDQCMKNTVFPFALPMLGAKVSVECKAADGSMMASAEATTDFLGGFVASFKGMADLEGCKAY
MKSSPDSSCSIAGYEGGGELKLRSKFFFTSFYGVEPMFFKPAAPKSFC
>RTMU_2181156_Calypogeia_fissa.2
GSVFCDQCLTHEVFPFSYPLKDARVSVQCYDSSGVHVVASATATTDFFGGYIATFKGVSDLGGCKAF
VSSSPDSSCNLVDGGGKTLSLKSKFLWDAFYIVDPLFYKPAHPKDFC
>ILBQ_2004952_Conocephalum_conicum.2
GNVFCDQCMKNEIFPFALPMHGAKVSVECMGADGRMAASAEATTDIFGGFWVSFKNAENMDGCRV
YMKSSPDSSCSMVGDQSGRAVQLKTKLFFSSFYAVEPMFFKPPAPKSFC
>CHJJ_2136182_Lejeuneaceae_sp.2
GSVFCDQCLKQAVFPFGLPLKGAKVTVQCYDASGKIVASGGATTDFIGGYAVTFKGASDLTGCKAFAT
SSPDSSCVLTESSGGQAVALKTKFIHNAFYVAEPLFFRPPQPKNFC
>TXVB_2074716_Lunularia_cruciata.2
GNVFCDQCMKNAVFPYARPMLGAKVSVQCYGADGSMLASADASTDFLGGFLASFKGVSDLEGCKVF
MKGSPDSSCNFADQSGQGVKLRTKFFFSSFYGVEPMFFKPPTPMGFC
>YFGP_2104693_Pallavicinia_lyellii.2
GSVFCDQCLKHEVFPFGLPLNGAKVSIQCYGASGANVVMSSEATTDFLGGYVATIKGVSDLGGCKAV
VTASPESSCNLVDGGAQALVLKTKFFFNAFYVVDPLFYMPAKPKDFC
>HPXA_2020358_Ptilidium_pulcherrimum.2
GGVYCDQCLKQEIFPFGLPMNDAKITVECYDGTGVNVVASATATTNFLGIYETSFKGVSDLSGCKAFV
TSSSQSSCSLAEGNGQTLDLKTKFFNNAFYSVDALFYKPAQPKGFC
>WJLO_2035800_Riccia_berychiana.2
GNVFCDQCMKNTVFPFALPLHGAKVSVQCAGADGKMMAAADANTDIFGGFLVTFKGVEDMGGCRV
YMKSSPDSSCNIVGNQGGLAVQLKTKLFFTSFYGVEPMFFKPPAPKSFC
>IRBN_2161692_Scapania_nemorosa.2
GSVFCDQCLKQEVFPFGRPLKGATVSVQCYDGAGVNVVASATATTDFFGGFIATFKGVSDLGGCKAV
VSSSPDSSCSVSDGGGQTLALKSKFLNHAFYFVDPLFYAPAKPDDFC
>HERT_2003915_Sphaerocarpos_texanus.2
GNVFCDQCMKNAVFPYALPMLGAKVSVQCYGAGGSMVASADASTDFFGGFVATFKGVSALDGCKV
VMKGSPDSSCSINADSGGKALELKTKFFFSSFYGVKGPMFYKPSAPKTFC
>Pp3c1_27500V3.2
GKVFCDRCIQNKVFPFAHPMSKAKVSVQCKDWSGRVVGYARTSTNFLGDFIVQFKGKKDLSGCSVYL
DGSSDRSCNIIGGGRRAISLKSKFLFQAFYVADPLFYKPARPMGFC
>Pp3c14_8250V3.2
GKVFCDQCIQNKVFPYAHPMSKAKVKVECKDASGNVVDYADASTNFLGDFIVSFKGKEDLTGCSVSL
AGSPDSKCSIVGGGGKTVTLKSKFLFKALYVVDPLFYKPALPMEFC
>YWNF_2047246_Hedwigia_ciliata.2
GKVFCDQCIQNKVFPFAHPMHDAKVSVKCKDKYGKVMDYAQASTNFLGDFIVSFKGKEDLSGCYVYL
SGSSDRKCNIVGGGGKTLNLKSKFLFKSVYVVDPLFYKPWKPMGFC
>TMAJ_2023569_Neckera_douglasii_2
GKVFCDQCIQSKVFPFAHPMNKAKVSVQCKDKSGKVVDYAQASTNFLGDFMVSFKGKEDLSGCYVY
LSGSSDRKCNIAGGGGKTLSLKSKFLFKAMYVVDPLFYKPATPMGFC
>BGXB_2078424_Plagiomnium_insigne.2

GKVFCDQCIQSKVFPFAHPMNKAKVSVQCKDQSGKVVDSAQASTNFLGDFMVSFKGKEDLAGCYVY
LSGSADSKCNIVGGGGKTLSLKSKFFFKAMYVVDPLFYKPAKPMGFC
>Sphfalx0052s0052.2
GSVFCDQCLQDRVFPFSEPMDGAKVTVACKDNNGNVVDSVQGSTDFLGNFLVTFKGKQDLSGCTVS
LAGSSRSDC
>SKQD_2012127_Takakia_lepidozioides.2
GSVFCDQCIQGRVFPFSYPLDGAKVTVQCKDAYGRVVDSAQGSTNFLGDFLITFKGKEDLSGCQAILS
GSPEAKCNIVGGGGRTMTLKSKLFFQALYVVDPLFYKPAQPAGIC
>QVMR_2006336_Psilotum_nudum.2
GSVYCDQCLDGHRSVWSIPLDGAKVAVECNDSKGQKVLSREGETGLFGDYAIRFDGSPHLGGCVVR
VLSSPKRTCNIAATGSKSISLSWKLFGLSFYSVDALSFRPSKAMSFC
>QVMR_2012184_Psilotum_nudum.2
GSVYCDRCIEGHRSVWGLPLDGAKVAVECKDSSGQMSLTKEGTTNMLGLYSVEFDGSPEMEGCGV
RALSSPSESCNVAGVTSQPVSLSWKLFSMAFYTGNALSYKPSKPMSFC
>ALVQ_2024672_Tmesipteris_parva.2
GSVYCDQCLDGRRSVWSLPLDGAKVTVECNNSKGQEILSREGDTGLFGDYVIRFDGSPNLGGCAVR
VLSSPKRTCNIVAIGSKSISLSWKLFGLSSYSADALSFRPSKAMSFC
>ALVQ_2102199_Tmesipteris_parva.2
GSVYCDQCVEGHRSIWGLPLDGAKVAVECKNSNGQISLSKQGTTNILGLYSVEFDGSPGMEGCGVR
ALSSPSGSCNISAVASQPVSLSWKMFSLAFYSGNALSYKPSKPMAFC
>NHCM_2064417_Angiopteris_evecta.2
GSVYCDQCLNGHRTMWSSPLNGAKVMVECKNSNGQVTLSKQGETDLLGMYSIKFDGSPDMQGCTA
KVVSSPKGPCNLAGTASNSVTLSWKLFGLAVYAAEALSYKPAKPSSIC
>ZZEI_2006244_Phylloglossum_drummondii.2
GTVFCDQCLIGEPSIWGPPMHDASVTLDCQNSGNSDQAKTSFIGTFVFKVGGDADLSSCTARLTGSS
SASCSLLGNAEKPLKLDWKIFGESFYSVAHVLLPHSGPTPPLVLPKPPPLSLPPSPFQDPSSC
>PQTO_2078178_Lycopodium_deuterodensum.2
GSVFCDQCLMGKPSIWGSPMHDANVTVDCKNSNNAIQSLHHASTSVLGTFVFTVSGDADLSSCSARL
TGSSSSSCSLFGNAEQSLKLEWKMFGEAFYSVEPMFFKPEKSMPFC
>S.moellendorfii_430897.2
GNVFCDQCLHGRPSMWGIPIRGAKVLVHCRNRQGMAMAVPAMSNFLGAFVANFPGEHDLQHCTSR
LMWSPLKQCNVVANAQQSLRLEWKILDNAFYTVKPLYFTPVKPLAIC
>ZFGK_2039051_Selaginella_kraussiana.2
GNVFCDQCLHGRPSMWAIPIQGAMVVVHCPSRQGVALQRRAYTNFLGAFIASFPTATLQECTSHLVW
SPMSECNNVVGSGRQQSLRLEWKMFDDSYYTTKPLYFKPLSPMSHC
>SGTW_2038568-Ginkgo_biloba.2
GNVFCDQCRDGHRTMFDLPINGAKVAVECTGSDGQITMSKQETTDLIGNFMMKFDGTPDLSGCTAR
VTDSPQDSDCKIVGSSSKPLSLSFRLFSMEVYTVESLFFQPAKPMSFC
>GNQG_2085018_Encephalartos_barteri.2
GNVFCDQCLDGLRTMFDLPLYGAQVRVDCRGDDGEITFSKEDSTDLSGNFVVKFDGIPDMSGCAAQ
VIGSPRGSDCNIVGSPARPLDLTFRLFAMEVYSVDPLFFHPAKPMPFC
>IZGN_2072084_Dacrydium_balansae.2
GNVFCDECQDGRRTLFDLPVNGAKVAIECNGQSNQVTTDLVGNFVMKFDGIPDLSGCSARVVQSPP
GSECNIVSSPAKPLTLTFSLFGMEMYTVESLSLEPAKPMSLC
>JRNA_2066102_Phyllocladus_hypohyllus.2
GNVFCDECQDGRRTLFDLPVSGAKVAVECNGQSNEVTTDLVGNYVLKFDGTPDLSGCSASLVQSPP
NSECNIVGSPAKRLSLTFSLFGMEMYSVESLSLQPAKPMSFC
>AmTr_v1.0_041.161.2
GTVFCDACKDGQRSSFDYPLSGAKVAVACAGNDGQVTVWKEESTNWFGNYGMRFEGSPDLSRCFA
QVVANPQGSNCGITGGPARGLNLVFDMFGMEMYVVDSLLSQPSQPMAFC
>P.equestris_XP_020587277.2
GSVFCDQCKDGSRGLFDYPLSGARVAVSCGDQYREESSNILGTYSVRFNGNPDLGGCLARVVRGPD
NCSVASGPAQSLKFIFRMFDMEAYSVDPLLAQPSKVMDFC
>Oropetium_25700A.2
GMVFCDQCKDGTRGLFDYPLYGARVAIQCGGGDTPLTVRESNTNWFGGFSIRMEGSPDMNRCTAR
VVQGTGHCGSSGAARELTLAFRMLGLALYTVPPLLSQPEESMDFC
>Sobic.004G001700.2
GMVFCDQCKDGARGLFDYPLYGARVAIQCGGGDTPLTVRESNTNWFGGFSVRMEGSPDMNRCTAR
VVQGTGHCGAGAGAGGGGAPRELTLAFRMLGLALYTVPPLLAQPEEAMDFC
>Zm00008a018944.2

GMVFCDQCKDGARGLFDYPLYGARVAIQCGGGDTPLTVRESNTNWFGGFSVRMEGSPDMNRCTAR
VVQGTGHCGAASSSGGGAVPRELTLAFRMLGLSLYTVPPLLSQPEEAMDFC
>Aco019419.2
GTVFCDQCKDGQRSIFDYPLSGATVAVECGSGGAVLKEDTTNWLGFYTVRVDGSQDLSACTARVVA
APSSCGAAAGPPRPLALMFRMLGMALYAAEEPLLSEPREPVGFC
>Aqcoe5G117200.2
GTVFCDQCKDGHRSLFDYPLSGMKVRVTCDSMEKEETTNWLGSYTVSFEGSPDLSGCYAHLLEDGQ
GSTKCGAVAGPAQQLKLMFKIFGMEMYAVDSLLSQPARPMSIC
>Lsat_1_v5_gn_8_126000.2
GSVFCDQCKDGQVSLFMDYPLTGVKVAMACPGQGGQLKVISEETTNFLGSYVMRFDGAPDMSGCR
AQVSGDGQGCRAVAGPAQSLNLVFQMFDTSIYTVGHLISQPAQPMPNC
>GSVIVT01024071001.2
GTVFCDQCKDGQRSLFDYPLYGIKVTMACANSNGQITMWREETTNWFGTYAMRFDGAPDLTGCYA
QISGSGQGSTGCRAAAGPAQSVRLMFRMFDMEMYAVDSLLSQPAQPMSFC
>Lus10026279.2
GSVFCDQCKDGQLSLFDYPMTGVRVTMACVDENGETLMSREERTNMFGWYNIRFDGTPDLRNCYT
QFAPDSSSTGEGSSSGGGSANSCIAAAGPPQKLRLMFNMFGMEMYSVDSLLSQPAKPMSFC
>Potri.004G169200.2
GTVFCDQCKDGQVSLFDYPIYGIKVTMTCADASGQITMSREETTNWFGNYVMTFDGSPDLSNCYAQV
SSSGQGSNACGAAAGPAQKLRLTFRMFDMEFYAVDSLLTEPSASMSFC
>Eucgr.G01761.2
GTVICDQCKDGRMSIFDYPIYGIKVMVACTNSDGQVTMSREETTNWFGNYGMRFEGTPDLSSCHAQ
VMGGSGQGSMGCSASASPAQPLRLTFQMFNMEFYSVDTLLSQPAQPMSFC
>Gorai.002G193700.2
GAVFCDQCKDGQRSLFDYPLSGMKVTVACADGTGQVTMSREETTNVFGNYVMRFDGTPDLSNCNA
QVSGSGEGSNDCGATAGPAQKLRLMFRMFGMEIYGVDSLLSEPSQPMSFC
>AT2G16630.2
GSVFCDQCKDGERSLFDFPVSGIKISVTCADENGQVYMSREETTNWLGGYVMRFDGTPDLSNCYAQ
VSDNGVQQDPSSCSIASGPAQKLKLMFSFFGIETFAADALLAQPVQPSSFC

Amino acid sequences of W-W domains

>Mapoly0014s0128.2
DQ**W**KNSDFFCH**W**KKATPFQTTVEIMFGKAAKKKYGDMNLYSALQGEDDLLKQAVAAMMNAYGNLKF
SMSPKAVKKEFLLALKGPQQLKWDTAWKLKQANLGWGKGKCMLAPCK
>RTMU_2181156_Calypogeia_fissa_2
DD**W**LNPKFFCY**W**KNATPHETTVGQMFGKAAEKKYGNISVYTALQSGDDLLKQAVAAMMNSNTNKQF
YMSRNEIKKKFNAALGGARQLQWETAWSFKQFNSGYGQGRCMM
>ILBQ_2004952_Conocephalum_conicum.2
EE**W**MKSEFFCH**W**KKVTPFQTTVGVMFGKAAKRKYGNMTLNQALQGQDDLLKQAVAAMMNSYVNPK
FEMSPRGVKRDFLLAMKGSQQLKWDTAWKLKQVNSAWGKGKCYLTACKF
>CHJJ_2136182_Lejeuneaceae_sp.2
DV**W**LNLDFLCQ**W**KNATPFDTTVGQMFGKAAQNKYGNISVYTALQQSGDNLLRQAVTAIMNSNTNAH
FYMSPKKVRNEFVKALQGSDQLKRETAWSFQQANSGFGSGKCMLSPC
>TXVB_2074716_Lunularia_cruciata.2
DE**W**MKSEFFCH**W**KKATPFQTTVPMMFGKASKKPYGNMNLYDALHGKDDLLKQAVAAMMNSYGNPK
FSMSPNGVKKEFLAALKGSQQLKWDTAWKLKQANMGWGKGKCLLAPCK
>YFGP_2104693_Pallavicinia_lyellii.2
GE**W**LNTGFFCN**W**KNATPFQTTVGQMFGKAAQKKYGNMSVYTALQSGDELLREGVAAMMNAYTNAH
FYMSPKAIKKDFNSGLLGNRQEQANIAWKFQQANSGYGKGKCMLSPC
>HPXA_2020358_Ptilidium_pulcherrimum.2
FD**W**QNTKYLCR**W**TKATPFQTTVGQMFGKAAQKKYGNLSVYTALQSGDDLLRQAVAATMNANSNEH
FYISAKSVKKQFNAALGGTQQLQWNTAYAFKQANSGYGTGKCMLTTC
>WJLO_2035800_Riccia_berychiana.2
DQ**W**KMSDFFCH**W**KKVSPFQTTVGMMFGKSAKRKYGNMTLLQALQGRDDLLKQAVAATMNSYINPK
FSMSPRGVKNEFLTAMKGPQKLKWETAWKLKQANGGWGKGKCYLTPCK
>IRBN_2161692 Scapania_nemorosa.2

ED**W**LNLKHVCH**W**TNANPYNTTVGHMFGAAAQKKYGNMPVYKGLQIGDELLKQSVAAMMNSYSNTH
FYMSPKEVRKRFNAALKGTQQLQWDTAWSFKEMNTGYGSGTC
>HERT_2003915_Sphaerocarpos_texanus.2
DE**W**MKSEFLCH**W**KKATPFQTSVGMMFGKDAKKAYGEMNLYTALRHQGQDDLLKQAVAAMMNSYS
SDKFGMTTAAVKDEFLKALNGSQQLKWDTAWKLKQANAGWGKGKCMLTPCK
>Pp3c1_27500V3.2
LD**W**LRPDYRCY**W**PKDLSPWKTSVGRVFGRGAQKKYGNKPLAGGLLTGDDLLRQAITALLNSRTNKH
FYMSPRGIKAKFSKALSGSWKAKSAQANAFRNANNGYGKGKCLLVPCKW
>Pp3c14_8250V3.2
LD**W**LRPDYKCY**W**PKDMQPWKTTVGTVFGKGAQQKYGSKTLAGGLLTEDELLRQAISAILNSRTNMH
FYMSPRAIQRSFAKALYASEPTKAAQAKAFKDANNGYGKGKCLLVSCKW
>YWNF_2047246_Hedwigia_ciliata.2
FD**W**LRPDYKCY**W**PKDMSPWKTTVGSVFGRGARKKYGYKTLAGGLLTGDELLRQAIAALLNSRTNQH
FYMSPRGIKSKFSKALWGSWAAKSAQAKAFQAANNGY
>TMAJ_2023569_Neckera_douglasii_2
CY**W**PKDMSP**W**KTTVGGVFGRGAQKKYGSKTLAGGLLTGDELLRQAIAALLNSRTNQHFYMSPRAIK
SKFSKALYGSWAAKSAQAKAFKSANNGYGKGKCLLVSCKW
>BGXB_2078424_Plagiomnium_insigne.2
AD**W**LRPDYKCY**W**PKDLSPWKTTVGSVFGKGAQKKYGYKTLAGGLLTGDELLRQAIAALLNSRTNQH
FYMSPRAIKGKFSKALSASWAAKSAQAKAFQAANNGYGKGKCLLVSCKW
>Sphfalx0052s0052.2
SD**W**LNIEYYCW**W**PKALSPLHTTVGSVFGKGAEKKYGNQSLVGGLLTGDELLRQSIAALLNSQTNLHF
YMSSRGVTRDFNNALLGSAANAATIANQLAAANSGYGRGKCLLVPCKL
>SKQD-2012127-Takakia_lepidozioides.2
YD**W**LHIDYFCW**W**PKTMSPFRTTVGRVFGRLAQKKYGNMNLLSALECSDPLLREATAALLNSYTNRHF
YMSSRRVVRDFNIALRGSPAVTANQAQAFHAANSGFGKGKCLLVPCK
>QVMR_2006336_Psilotum_nudum.2
ND**W**LDPQFKCY**W**RLLSPDTKVSVLVLLREAITSSLNAFNSIHFYYSPVSVHVHFNQAIKGTDKDVTRW
ALAFKRANSGYGGKAKCLMTKC
>QVMR_2012184_Psilotum_nudum.2
YY**W**LKPEYRCY**W**RLLSPNTTISVFYGEAAAKKYGANITLWEGLQGGGDVQKVLLRESITASLNAFNSL
KFYYNAIAVDVHFNEAINGTDNDAVKWGSEFRSANSGYG
>ALVQ_2024672_Tmesipteris_parva.2
ND**W**LDPQFKCY**W**RLLSPSTKVSVFYGETAGKKYGSNITLWEGLHGSGDVNKVLLREAITSSLNAFNSI
PFYYNAVSVHVHFNQAIKGTDKDVTRWALAFKRANSGYGGKAKCLMTKCK
>ALVQ_2102199_Tmesipteris_parva.2
YN**W**LKPEYRCY**W**RLLSPNTTISVFYGEAASNKYGENTTLWDGLQGGGDVQKVLLRESITASLNAFNS
WKFYYNAITVDVHFNEAIRGTDKDAVKWGSAFQSANSGYGGKSKCLMTVCK
>NHCM_2064417_Angiopteris_evecta.2
YY**W**TNPQYRCY**W**RLTSPDWKVSSYYGEAAGKRYGANKTLWQGLQGRGELYGALLREGITSSLNAL
NSAKFYYNAVEVNIHLNEALKGTTKDALKWMLAFRRANNGYGGKAKCLMSVCK
>ZZEI_2006244_Phylloglossum_drummondii.2
DE**W**MKPEYRCY**W**KFASPNETVVEAFGPAAAHRYGTNTTLMDALSGGGGGGKLRGRSKAYRTLLPE
AVAALLNAYSSWKFKYAPWAVAAEFTEVVVRNSPRGAAKLAEDFRTANSEHKCLLQACK
>PQTO_2078178_Lycopodium_deuterodensum.2
DQ**W**AKPEHLCY**W**KFSSPNETAREAFGPAVAHHYGDTTLMDALNGKSTAVEMIYRSRSYRVLLRESV
TALLNSYSSVKFKYSPLTVIAQFTDVIASHSSRAAAKLAHDFQTANSGGKCLLQTCK
>S.moellendorfii_430897.2
DH**W**MNPVYSCH**W**KFVTPDESIAKLLGPRARDKYGNITALEALVKNSSLNGIADQHYATTYNGLLSETV
AALLNSYYNSRFSLSPMTISRIFDNVIARGTPKDASKIAAEFRRANAGLGKGTCLLDVCKSK
>ZFGK_2039051_Selaginella_kraussiana.2
DH**W**KNPRYLCN**W**RLLAPGQLVGDVLGWSAEARYGNLTLLQALENGPTLVSESLAALLNSYFSSKFPY
SPLWIASSFKHALSHGTANRLASQLRKANAGLGKGHCLLQPC
>SGTW_2038568-Ginkgo_biloba.2
EY**W**VNPAYNCY**W**RLARPDTTVSLAFGEEAAKKYGRNMTLWEGLYGRGDVYRTLLREATASLLNSYN
SFQFQYNTLSVFLHMNSALQGSPQDALKQALYFKRANTGGGKIKCSLTPCK
>GNQG_2085018_Encephalartos_barteri.2
EY**W**VKPEFRCY**W**KLVNPNTTVGAAFGEVAGSKYGKNLSLWDALFGRADVYKTLLREATASLLNSYSS
FLFQYNALTVVDRLNSALQGSPRDALIQALKFKKANYGRGKAKCLFIPCK
>IZGN_2072084_Dacrydium_balansae.2

DF**W**VRPEYACS**W**RVVRPDTKVGVVFGAEAANKFGHNMTLWEGLHGKGDVYRTLLREGTASLLNSY
NSLSFCYNTLSVIMHMHSALHSGSPQYALKQAFYFKKANIGGGSTKCLLKPCK

>JRNA_2066102_Phyllocladus_hypohyllus.2
DL**W**RKPEYACY**W**RVVRPDTKVGVVFGEEALNKFGQNMTLLEGLYGRGDVYRTLLREGTASLLNSYN
SFSFSYNTLSVVMHMHSALHSGSPRYALKQAFHFKKANIGGRNTKCLLKPCK

>AmTr_v1.0_041.161.2
EY**W**LMPEYRCY**W**KVVGPGTKVAVAFGPAAAQKYGTDLPLYEALQGRGDLYRTLLREATTALLNSYN
SLQFGYPTLTVVGNMNWALQASPQEALRQAMRFKRANAGTPETPCRFTPCN

>P.equestris_XP_020587277.2
SK**W**LMPAYKCY**W**KMVSPETPVAVVFGPVAAGKYGTDMSLWTALHGRGDVYRTLLREATAALLNSYN
SLSFPYPTLSVISHMNSALLGSQREALMTALRFRRANSGVAGHSTVACNFTPCS

>Oropetium_25700A.2
EK**W**AAPEQRCR**W**KVVTPNTTVAMAFGPLAAQRYGSELTLREALEGRGDMYRTLLREATAALLNAYY
NAPGGPFLYPTTASVIDHMNGALLSSAQRVLIEGARFRRANAGGGGPAGRTRLTCDFATPCAPAAEA
PQSGLESRHY

>Sobic.004G001700.2
DM**W**ASPEHRCH**W**KVLTPNTTVAMAFGPLAAQRYGSELTLGEALEGRGDMYRTLLREATAALLNAYY
NAPGGPFLYPTTASVMDHMNGALLSSGQRVLLEGARFRRANAGGGGPAGRTRLPCDFTPCGSCAP
PTS

>Zm00008a018944.2
DM**W**SSPEHRCH**W**KVVTPNTTVAMAFGPLAAQRYGSELTLGEALEGRGDMYRTLLREATAALLNAYY
NAPGGPFLYPTTASVMDHMNGALLSSGQRVLLEGARFRRANAGGGGRTRLMCDFTTCS

>Aco019419.2
DK**W**ITPDYMCY**W**KVVSPGTSVALAFGPVAAARYGMDMTLWQGLQGRGDVYRTLLREATTSLLNSYN
SLGFFYPTLSVIELTNLALLGSPQQALMTALRFRRANAGVAGRGTNATCNFTPCS

>Aqcoe5G117200.2
EI**W**TRPEYRCH**W**TFVQPDTKVAVAFGLIAARKYGNDMSLWQGIQGRGNIYRTLLREGTTALLNSYNSI
QFHYPTLSVLYHMNSALMGTPRQALMTALRFKRANSGYGNSGARCNLTPCK

>Lsat_1_v5_gn_8_126000.2
RM**W**TMPEHECY**W**RVLAPDLKVSFVFGPLAARKYGNDITLRGSMTGRGDPYKTLLREATTALLNSYNS
IEFPYHPLDVVQHLNMALIGGSTRQVLITALQFLRANSGRPGNVTCKFTTCK

>GSVIVT01024071001.2
EI**W**TAPEYKCY**W**KVVNPDMKVASVFGLLAAQKYGTDMTLREGLQGRGDPYRTLLREAITSLLNSYNS
LLFPYHTLGVIQHMSLALMGSQQNVLLTALRFKRANSGSGNLTCKFTACK

>Lus10026279.2
QT**W**TAAEYRCF**W**RIVTPETKVAVAFGLPAAQRYGTEMTLLEALQGRGDPYRTLLREATTSLLNSYNS
LRFRYDAVSVVARTNYALLGSQRDALYTALRFIRANSGSPCAAPGSPAVTCRLSPCK

>Potri.004G169200.2
QS**W**TMPEYKCY**W**RAVSPDMKVAVVFGLLAARRYGTDMTLWQGLQGRGDPYRTLLREGTAALLNSY
NSIEFPYNAISVVTRMNWALMGSQRSVLLTALRFMRANSGYGRVTCKFNTCK

>Eucgr.G01761.2
EK**W**MMSEYKCY**W**RAVNPDTKVALVFGLTAARRYGTGLTLWQGLQGRGDPYRTLLREATTALLNSYS
SIQFPYHTLGVLQHMNSALMGSTQNVLLTALRFRRANLGYGTTTCKFTPCK

>Gorai.002G193700.2
QY**W**TMPEYKCY**W**RVLNPETKVSLIFGPLAARRYGSDMTLWESLQGRGDPYKTLLREATTALLNSYNT
LQFPYNSIGVVTRTNWALMGSTRGVLITALRFIRANSGSGRVTCKLTPCKS

>AT2G16630.2
QL**W**MKPEYRCY**W**RAIGPDTKVAVAFGLVAGRIYGTDMTVREALDGRGEAYKTLLREATTALLNSYNS
LGFPYNSVAVITYTNLALLGNSEHDVLMTAIRFIKANSGTCRFTVCN
