# Supplementary material for: The Cell Wall PAC (Proline-Rich, Arabinogalactan Proteins, Conserved Cysteines) Domain-Proteins Are Conserved in the Green Lineage
Source: Int J Mol Sci. 2020 Apr 3;21(7):2488. doi: 10.3390/ijms21072488 (PMC7177597; doi:10.3390/ijms21072488)
Supplement: Supplementary file 1 [file ijms-21-02488-s001.zip › Supplementary files_R1_Nguyen-Kim, San Clemente et al/Nguyen-Kim, San Clemente et al._Supplementary file 8.pdf]

Supplementary file 8

Top-scoring 3D-models of PAC domains and the corresponding scores (page 2)

3D-model of the *C. orbicularis* putative PAC domain (GBSL01052221) (page 3)

3D-models of proteins exhibiting α-helices (page 4)

ILBQ_2006312_Conocephalum_conicum

YFGP_2007785_Pallavicinia_lyellii

Supplementary file 8. Top-scoring 3D-models of PAC domains and the corresponding scores

Both I-Tasser and MODELLER tools usually provide similar models in simple modeling tasks - if a solid template structure is available. The models differ more if they are less reliable. Thus, the root-mean-square deviation (RMSD) between the models can provide an additional sound estimation of the reliability of the prediction, besides the scores. The more resulting structures differ, the less reliable they are.
The table below provides RMSD values based on the CA-atoms between the top-scoring MODELLER and I-Tasser models. Only the structures of domains with RMSD values higher than 10.0 are considered for further analyses.

I-Tasser was not able to find conformations which enables disulfide bridges between the predefined binding partners
PAC domain in bold characters are those shown in Figure 5.
* lower score is better

PAC domain	Modeling tool	Sequence identity	Model	DOPE*	MAESTRO*	ProSA 2003*	RMSD between top scoring MODELLER and I-Tasser model*	Comments
**Oropetium_11363A.2**	I-Tasser	12,5	Oropetium_11363A.2_model1.pdb	-0,032464677	-1,8025527	-7,6	3,884751081	
AT5G10130.1	I-Tasser	20,3	AT5G10130.1_model1.pdb	-0,454205842	-2,244394931	-6,79	4,281828403	
AmTr_v1.0_00047.2	I-Tasser	17,6	AmTr_v1.0_00047.2_model1.pdb	-0,410957408	-1,384055711	-7,16	4,322821617	
Gorai.002G165500.1	I-Tasser	9,9	Gorai.002G165500.1_model1.pdb	-1,076360751	-2,000915032	-6,1	4,361501694	no Gly upstream Cys 1
TFDQ_2013818_Monoclea_gottsche	I-Tasser	18,4	TFDQ_2013818_Monoclea_gottsche_model1.pdb	-0,390457154	-1,190396533	-7,33	4,415602207	
AmTr_v1.0_061.7.1	I-Tasser	22,8	AmTr_v1.0_061.7.1_model1.pdb	-1,047867733	-2,291373767	-6,72	4,424602509	
AmTr_v1.0_062.88.1	I-Tasser	15,0	AmTr_v1.0_062.88.1_model1.pdb	0,118184404	-1,788505066	-6,16	4,51101923	
ILBQ_2006312_Conocephalum_coni	I-Tasser	11,2	ILBQ_2006312_Conocephalum_coni_model1.pdb	-0,267583162	-0,656269559	-5,97	4,732092381	prediction of α-helices
AmTr_v1.0_019.68.1	I-Tasser	11,9	AmTr_v1.0_019.68.1_model1.pdb	-0,048679557	-1,340679231	-7,27	4,749872208	no Gly upstream Cys 1
**AT1G28290.3**	I-Tasser	21,6	AT1G28290.3_model1.pdb	-0,322687102	-1,348849379	-6,44	4,759518147	
AT2G34700.3	I-Tasser	17,6	AT2G34700.3_model1.pdb	-0,204833192	-1,547771425	-7,48	4,820729256	
Bradi3g20970	I-Tasser	14,4	Bradi3g20970_model1.pdb	-0,210726377	-1,542712873	-7,09	4,84980917	
Mapoly0014s0128.2	I-Tasser	18,3	Mapoly0014s0128.2_model1.pdb	0,032723491	-0,919309931	-6,94	4,858971596	
AmTr_v1.0_066.9.4	I-Tasser	25,6	AmTr_v1.0_066.9.4_model1.pdb	-0,799350464	-1,220401257	-7,38	4,883558273	
Mapoly0139s0006.2.4	I-Tasser	15,2	Mapoly0139s0006.2.4_model1.pdb	0,079750506	-2,031587116	-7,22	4,911189556	prediction of α-helices
AmTr_v1.0_041.161.2	I-Tasser	14,3	AmTr_v1.0_041.161.2_model1.pdb	-0,143505494	-1,82528676	-5,83	5,180187702	
TFDQ_2117810_Monoclea_gottsche	I-Tasser	22,9	TFDQ_2117810_Monoclea_gottsche_model1.pdb	-0,804771044	-1,306090621	-7,16	5,275324821	
Bradi3g21030.2	I-Tasser	12,3	Bradi3g21030.2_model1.pdb	-0,364703605	-0,591656625	-6,46	5,327032566	
HVBQ_2020253_Tetraphis_pelluci	I-Tasser	12,0	HVBQ_2020253_Tetraphis_pelluci_model1.pdb	0,437950655	-0,500743718	-6,83	5,332158089	prediction of α-helices
AT5G54855.1	I-Tasser	18,3	AT5G54855.1_model1.pdb	-0,188869501	-1,42199867	-7,35	5,466360569	
CHJJ_2011674_Lejeuneaceae_sp_n	I-Tasser	13,6	CHJJ_2011674_Lejeuneaceae_sp_n_model1.pdb	-0,800172013	-2,273718658	-6,75	5,495284557	prediction of α-helices
RXRQ_2140319_Phaeoceros_caroli	I-Tasser	20,8	RXRQ_2140319_Phaeoceros_caroli_model1.pdb	0,000180931	-1,18472794	-5,6	5,543737888	
Bradi3g20980.2	I-Tasser	13,6	Bradi3g20980.2_model1.pdb	-0,557693647	-1,680107732	-6,6	5,630766392	
AmTr_v1.0_153.4.1	I-Tasser	20,0	AmTr_v1.0_153.4.1_model1.pdb	0,047110306	-0,906467314	-6,1	5,81362772	
**IQJU_2004004_Anthoceros_formos**	I-Tasser	14,8	IQJU_2004004_Anthoceros_formos_model1.pdb	-0,036337289	-0,363583909	-6,19	5,971568108	
**AmTr_v1.0_068.122.1**	I-Tasser	15,2	AmTr_v1.0_068.122.1_model1.pdb	-0,196016121	-1,426214731	-6,35	6,117260933	
Oropetium07184A.3	I-Tasser	24,0	Oropetium07184A.3_model1.pdb	-0,806702421	-1,219163372	-6,95	6,120306015	
AT3G09925.1	I-Tasser	16,8	AT3G09925.1_model1.pdb	-0,032861326	-1,224853771	-4,81	6,134305477	
AmTr_v1.0_047.45.1	I-Tasser	16,8	AmTr_v1.0_047.45.1_model1.pdb	-0,660871901	-1,629893761	-7,42	6,3801651	
AT5G15780.2	I-Tasser	20,0	AT5G15780.2_model1.pdb	-0,746161239	-1,384316548	-7,59	6,560771465	
Oropetium_17805A.1	I-Tasser	23,8	Oropetium_17805A.1_model1.pdb	-0,836394711	-1,092954176	-7,35	6,725212097	
GBSL01052221.1_6_Coleochaete	I-Tasser	16,4	GBSL01052221.1_6_Coleochaete_model1.pdb	-0,204761625	-1,851821353	-5,89	6,756970882	7 Cys, prediction of a-helices
IQJU_2076581_Anthoceros_formos	I-Tasser	22,0	IQJU_2076581_Anthoceros_formos_model1.pdb	-0,554490867	-1,243160888	-6,67	6,958469868	
AT5G05500.3	I-Tasser	17,6	AT5G05500.3_model1.pdb	0,186798645	-0,840101526	-6,26	7,12987709	
WNGH_2084316_Aulacomnium_heter	I-Tasser	30,4	WNGH_2084316_Aulacomnium_heter_model1.pdb	-0,945875405	-2,172397247	-7,44	7,296413422	
Bradi3g21000.2	I-Tasser	14,4	Bradi3g21000.2_model1.pdb	-0,303142539	-1,418778655	-7,86	7,453835487	
Oropetium_03649A.4	I-Tasser	16,0	Oropetium_03649A.4_model1.pdb	-0,216938665	-2,153989215	-5,66	7,76369381	
QVMR_2011678_Psilotum_nudum.nd	I-Tasser	15,2	QVMR_2011678_Psilotum_nudum.nd_model1.pdb	-0,495463495	-0,747178083	-5,23	8,444963455	prediction of α-helices
Lus10026281.2	I-Tasser	15,7	Lus10026281.2_model1.pdb	0,517180007	-1,450888971	-6,48	8,625576019	prediction of α-helices
TFDQ_2004419_Monoclea_gottsche	I-Tasser	18,4	TFDQ_2004419_Monoclea_gottsche_model1.pdb	-1,001793526	-1,515026176	-7,52	8,810534477	
WNGH_2083088_Aulacomnium_heter	I-Tasser	26,4	WNGH_2083088_Aulacomnium_heter_model1.pdb	-0,567925533	-1,671295764	-6,86	8,882749558	
AmTr_v1.0_041.169.2	I-Tasser	15,6	AmTr_v1.0_041.169.2_model1.pdb	-0,466132691	-1,189647673	-7,53	11,09608364	
Bradi3g21010.2	I-Tasser	14,8	Bradi3g21010.2_model1.pdb	-0,349565123	-0,847062522	-7,2	11,56159878	
Oropetium_03650A.3	I-Tasser	12,8	Oropetium_03650A.3_model1.pdb	-0,836217324	-1,043942678	-5,28	12,05586529	
UCRN_2050882_Megaceros_tosanus	I-Tasser	9,6	UCRN_2050882_Megaceros_tosanus_model1.pdb	-0,300128844	-1,851226693	-5,79	14,60367012	prediction of α-helices
Oropetium_22028A.1	I-Tasser	16,0	Oropetium_22028A.1_model1.pdb	-0,700727846	-1,150744996	-6,95	15,68772411	
AmTr_v1.0_019.72.1	I-Tasser	15,2	AmTr_v1.0_019.72.1_model1.pdb	-0,517700354	-1,35219022	-6,55	16,10477257	
YFGP_2007785_Pallavicinia_lyel	I-Tasser	11,9	YFGP_2007785_Pallavicinia_lyel_model1.pdb	-0,069022948	-1,164291398	-4,31	17,43003654	prediction of α-helices
AT2G16630.2	modeller	13,0	AT2G16630.2_model1.pdb	-0,372392901	-0,976095088	-6,94	17,95002937	
Oropetium_25700A.2	modeller	15,5	Oropetium_25700A.2_model1.pdb	-0,439591223	-1,368648691	-5,99	19,16456223	

Supplementary file 8

3D-model of the *C. orbicularis* putative PAC domain (GBSL01052221)

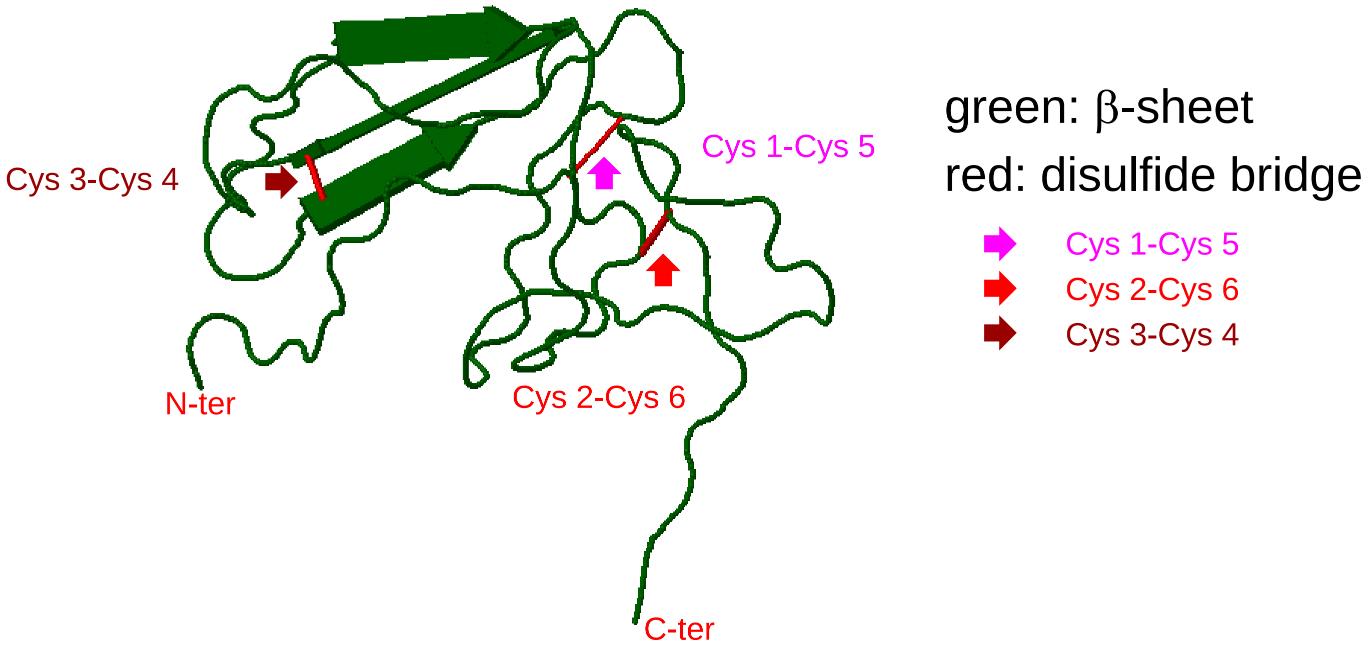

3D-models of proteins exhibiting α-helices

ILBQ_2006312_Conocephalum_conicum

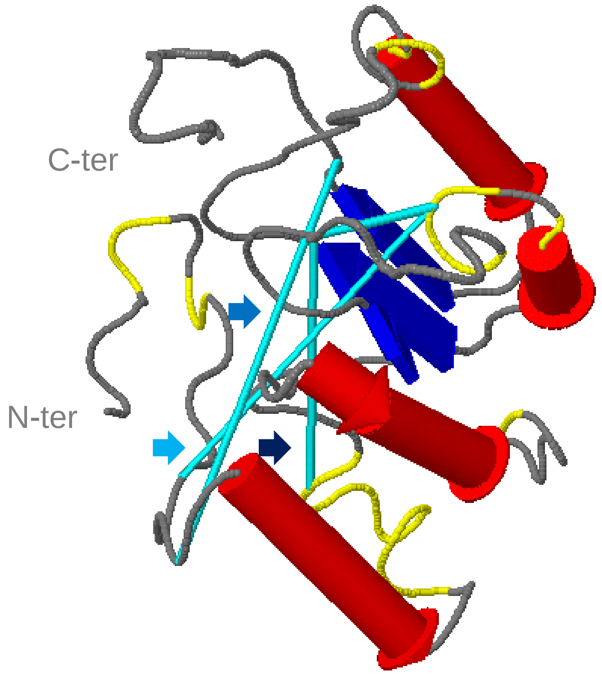

YFGP_2007785_Pallavicinia_lyellii

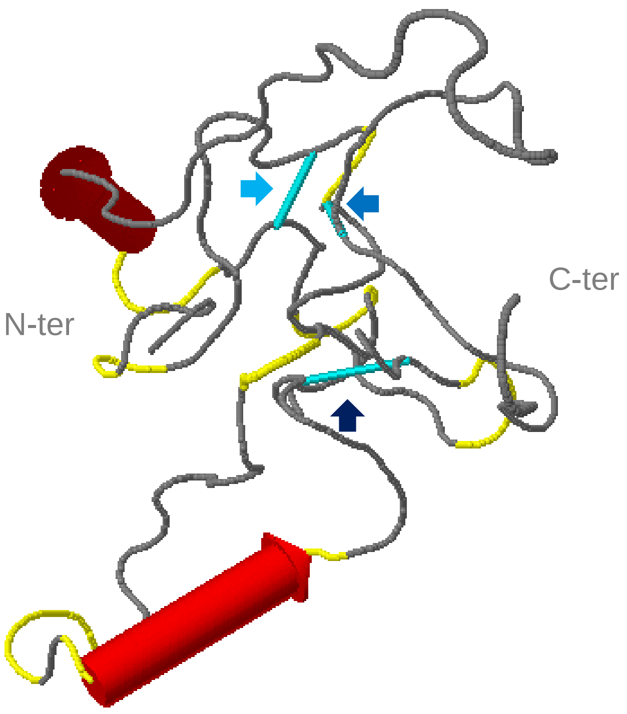

red: α-helix

blue: β-sheet

cyan: disulfide bridge

- Cys 1-Cys 5
- Cys 2-Cys 6
- Cys 3-Cys 4
