# Supplementary material for: The Cell Wall PAC (Proline-Rich, Arabinogalactan Proteins, Conserved Cysteines) Domain-Proteins Are Conserved in the Green Lineage
Source: Int J Mol Sci. 2020 Apr 3;21(7):2488. doi: 10.3390/ijms21072488 (PMC7177597; doi:10.3390/ijms21072488)
Supplement: Supplementary file 1 [file ijms-21-02488-s001.zip › Supplementary files_R1_Nguyen-Kim, San Clemente et al/Nguyen-Kim, San Clemente et al._Supplementary file 9.pdf]

Supplementary file 9. Expanded phylogenetic trees of PAC domains from Bryophytes to *A. trichopoda* (Tree I) and from *A. trichopoda* to Brassicales (Tree II).

Calculations have been performed using PROMALS3D (http://prodata.swmed.edu/promals3d/promals3d.php) for sequence alignment according to secondary structures and MEGA7 (https://www.megasoftware.net/) for phylogenetic tree building using the Maximum Likelihood statistical method with 500 bootstraps.

The evolutionary history was inferred by using the Maximum Likelihood method based on the JTT matrix-based model [Jones *et al.* 1992]. The percentage of trees in which the associated taxa clustered together is shown next to the branches. Initial tree(s) for the heuristic search were obtained automatically by applying Neighbor-Join and BioNJ algorithms to a matrix of pairwise distances estimated using a JTT model, and then selecting the topology with superior log likelihood value. The tree is drawn to scale, with branch lengths measured in the number of substitutions per site. All positions containing gaps and missing data were eliminated. Evolutionary analyses were conducted in MEGA7 [Kumar *et al.* 2016].

Tree I (page 2). The tree with the highest log likelihood (-9717.26) is shown. The analysis involved 116 amino acid sequences. There were a total of 58 positions in the final dataset.

Tree II (page 3). The tree with the highest log likelihood (-14586,02) is shown. The analysis involved 197 amino acid sequences. There were a total of 60 positions in the final dataset.

Jones DT, Taylor WR, Thornton JM (1992) The rapid generation of mutation data matrices from protein sequences. Computer Applications in the Biosciences 8: 275-282

Kumar S, Stecher G, Tamura K (2016). MEGA7: Molecular evolutionary genetics analysis version 7.0 for bigger datasets. Molecular Biology and Evolution 33: 1870-1874

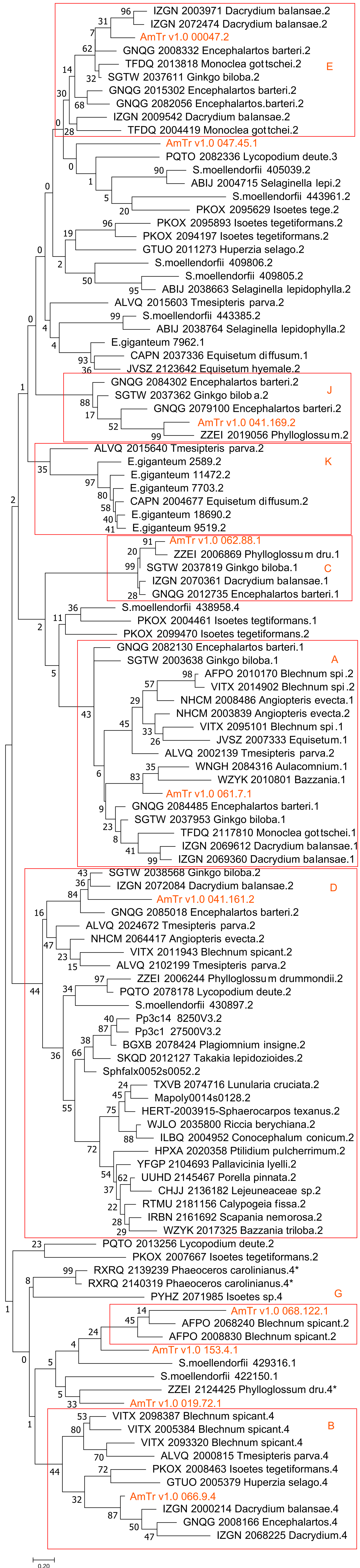

IZGN 2003971 Dacrydium balansae.2
IZGN 2072474 Dacrydium balansae.2
AmTr v1.0 00047.2
GNQG 2008332 Encephalartos barteri.2
TFDQ 2013818 Monoclea gottschei.2
SGTW 2037611 Ginkgo biloba.2
GNQG 2015302 Encephalartos barteri.2
GNQG 2082056 Encephalartos.barteri.2
IZGN 2009542 Dacrydium balansae.2
TFDQ 2004419 Monoclea gottchei.2
E
AmTr v1.0 047.45.1
PQTO 2082336 Lycopodium deute.3
S.moellendorfii 405039.2
ABIJ 2004715 Selaginella lepi.2
S.moellendorfii 443961.2
PKOX 2095629 Isoetes tege.2
PKOX 2095893 Isoetes tegetiformans.2
PKOX 2094197 Isoetes tegetiformans.2
GTUO 2011273 Huperzia selago.2
S.moellendorfii 409806.2
S.moellendorfii 409805.2
ABIJ 2038663 Selaginella lepidophylla.2
ALVQ 2015603 Tmesipteris parva.2
S.moellendorfii 443385.2
ABIJ 2038764 Selaginella lepidophylla.2
E.giganteum 7962.1
CAPN 2037336 Equisetum diffusum.1
JVSZ 2123642 Equisetum hyemale.2
GNQG 2084302 Encephalartos barteri.2
SGTW 2037362 Ginkgo biloba.2
GNQG 2079100 Encephalartos barteri.2
AmTr v1.0 041.169.2
ZZEI 2019056 Phylloglossum.2
J
ALVQ 2015640 Tmesipteris parva.2
E.giganteum 2589.2
E.giganteum 11472.2
E.giganteum 7703.2
CAPN 2004677 Equisetum diffusum.2
E.giganteum 18690.2
E.giganteum 9519.2
K
AmTr v1.0 062.88.1
ZZEI 2006869 Phylloglossum dru.1
SGTW 2037819 Ginkgo biloba.1
IZGN 2070361 Dacrydium balansae.1
GNQG 2012735 Encephalartos barteri.1
C
S.moellendorfii 438958.4
PKOX 2004461 Isoetes tegtiformans.1
PKOX 2099470 Isoetes tegetiformans.2
GNQG 2082130 Encephalartos barteri.1
SGTW 2003638 Ginkgo biloba.1
AFPO 2010170 Blechnum spi.2
VITX 2014902 Blechnum spi.2
NHCM 2008486 Angiopteris evecta.1
NHCM 2003839 Angiopteris evecta.2
VITX 2095101 Blechnum spi.1
JVSZ 2007333 Equisetum.1
ALVQ 2002139 Tmesipteris parva.2
WNGH 2084316 Aulacomnium.1
WZYK 2010801 Bazzania.1
AmTr v1.0 061.7.1
GNQG 2084485 Encephalartos barteri.1
SGTW 2037953 Ginkgo biloba.1
TFDQ 2117810 Monoclea gottschei.1
IZGN 2069612 Dacrydium balansae.1
IZGN 2069360 Dacrydium balansae.1
A
SGTW 2038568 Ginkgo biloba.2
IZGN 2072084 Dacrydium balansae.2
AmTr v1.0 041.161.2
GNQG 2085018 Encephalartos barteri.2
ALVQ 2024672 Tmesipteris parva.2
NHCM 2064417 Angiopteris evecta.2
VITX 2011943 Blechnum spicant.2
ALVQ 2102199 Tmesipteris parva.2
ZZEI 2006244 Phylloglossum drummondii.2
PQTO 2078178 Lycopodium deute.2
S.moellendorfii 430897.2
Pp3c14 8250V3.2
Pp3c1 27500V3.2
BGXB 2078424 Plagiomnium insigne.2
SKQD 2012127 Takakia lepidozioides.2
Sphfalx0052s0052.2
TXVB 2074716 Lunularia cruciata.2
Mapoly0014s0128.2
HERT-2003915-Sphaerocarpos texanus.2
WJLO 2035800 Riccia berychiana.2
ILBQ 2004952 Conocephalum conicum.2
HPXA 2020358 Ptilidium pulcherrimum.2
YFGP 2104693 Pallavicinia lyelli.2
UUHD 2145467 Porella pinnata.2
CHJJ 2136182 Lejeuneaceae sp.2
RTMU 2181156 Calypogeia fissa.2
IRBN 2161692 Scapania nemorosa.2
WZYK 2017325 Bazzania triloba.2
D
PQTO 2013256 Lycopodium deute.2
PKOX 2007667 Isoetes tegetiformans.2
RXRQ 2139239 Phaeoceros carolinianus.4*
RXRQ 2140319 Phaeoceros carolinianus.4*
PYHZ 2071985 Isoetes sp.4
G
AmTr v1.0 068.122.1
AFPO 2068240 Blechnum spicant.2
AFPO 2008830 Blechnum spicant.2
AmTr v1.0 153.4.1
S.moellendorfii 429316.1
S.moellendorfii 422150.1
ZZEI 2124425 Phylloglossum dru.4*
AmTr v1.0 019.72.1
VITX 2098387 Blechnum spicant.4
VITX 2005384 Blechnum spicant.4
VITX 2093320 Blechnum spicant.4
ALVQ 2000815 Tmesipteris parva.4
PKOX 2008463 Isoetes tegetiformans.4
GTUO 2005379 Huperzia selago.4
AmTr v1.0 066.9.4
IZGN 2000214 Dacrydium balansae.4
GNQG 2008166 Encephalartos.4
IZGN 2068225 Dacrydium.4
B
0.20

Supplementary file 9. Expanded phylogenetic trees of PAC domains from Bryophytes to *A. trichopoda* (Tree I) and from *A. trichopoda* to Brassicales (Tree II).

Calculations have been performed using PROMALS3D (http://prodata.swmed.edu/promals3d/promals3d.php) for sequence alignment according to secondary structures and MEGA7 (https://www.megasoftware.net/) for phylogenetic tree building using the Maximum Likelihood statistical method with 500 bootstraps.

The evolutionary history was inferred by using the Maximum Likelihood method based on the JTT matrix-based model [Jones *et al.* 1992]. The percentage of trees in which the associated taxa clustered together is shown next to the branches. Initial tree(s) for the heuristic search were obtained automatically by applying Neighbor-Join and BioNJ algorithms to a matrix of pairwise distances estimated using a JTT model, and then selecting the topology with superior log likelihood value. The tree is drawn to scale, with branch lengths measured in the number of substitutions per site. All positions containing gaps and missing data were eliminated. Evolutionary analyses were conducted in MEGA7 [Kumar *et al.* 2016].

Tree I (page 2). The tree with the highest log likelihood (-9717.26) is shown. The analysis involved 116 amino acid sequences. There were a total of 58 positions in the final dataset.

Tree II (page 3). The tree with the highest log likelihood (-14586,02) is shown. The analysis involved 197 amino acid sequences. There were a total of 60 positions in the final dataset.

Jones DT, Taylor WR, Thornton JM (1992) The rapid generation of mutation data matrices from protein sequences. Computer Applications in the Biosciences 8: 275-282

Kumar S, Stecher G, Tamura K (2016). MEGA7: Molecular evolutionary genetics analysis version 7.0 for bigger datasets. Molecular Biology and Evolution 33: 1870-1874

Tree II

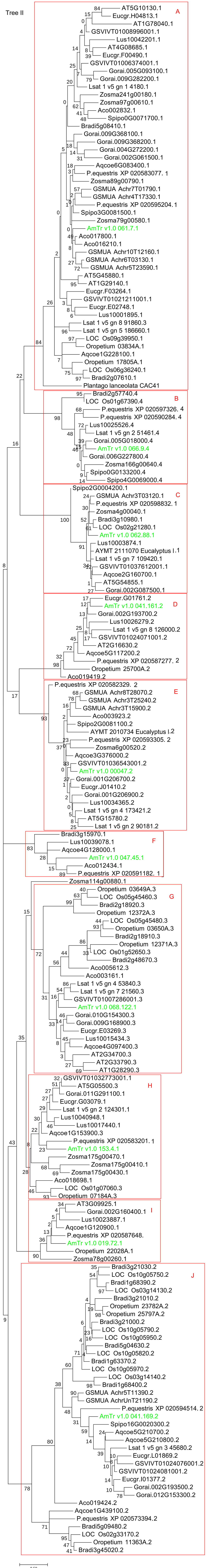
A
AT5G10130.1
Eucgr.H04813.1
AT1G78040.1
GSVIVT01008996001.1
Lus10042201.1
AT4G08685.1
Eucgr.F00490.1
GSVIVT01006374001.1
Gorai.005G093100.1
Gorai.009G282200.1
Lsat 1 v5 gn 1 4180.1
Zosma241g00180.1
Zosma97g00610.1
Aco002832.1
Spipo0G0071700.1
Bradi5g08410.1
Gorai.009G368100.1
Gorai.009G368200.1
Gorai.004G272200.1
Gorai.002G061500.1
Aqcoe6G083400.1
P.equestris XP 020583077. 1
Zosma89g00790.1
GSMUA Achr7T01790.1
GSMUA Achr4T17330.1
P.equestris XP 020595204.1
Spipo3G0081500.1
Zosma79g00580.1
AmTr v1.0 061.7.1
Aco017800.1
Aco016210.1
GSMUA Achr10T12160.1
GSMUA Achr6T03130.1
GSMUA Achr5T23590.1
AT5G45880.1
AT1G29140.1
Eucgr.F03264.1
GSVIVT01021211001.1
Eucgr.E02748.1
Lus10001895.1
Lsat 1 v5 gn 8 91860.3
Lsat 1 v5 gn 5 186660.1
LOC Os09g39950.1
Oropetium 03834A.1
Aqcoe1G228100.1
Oropetium 17805A.1
LOC Os06g36240.1
Bradi2g07610.1
Plantago lanceolata CAC41
B
Bradi2g57740.4
LOC Os01g67390.4
P.equestris XP 020597326. 4
P.equestris XP 020590284. 4
Lus10025526.4
Lsat 1 v5 gn 2 51461.4
Gorai.005G018000.4
AmTr v1.0 066.9.4
Gorai.006G227800.4
Zosma166g00640.4
Spipo0G0133200.4
Spipo4G0069000.4
C
Spipo2G0004200.1
GSMUA Achr3T03120.1
P.equestris XP 020598832. 1
Zosma4g00040.1
Bradi3g10980.1
LOC Os02g21280.1
AmTr v1.0 062.88.1
Lus10003874.1
AYMT 2111070 Eucalyptus l.1
Lsat 1 v5 gn 7 109420.1
GSVIVT01037612001.1
Aqcoe2G160700.1
AT5G54855.1
Gorai.002G087500.1
D
Eucgr.G01761.2
AmTr v1.0 041.161.2
Gorai.002G193700.2
Lus10026279.2
Lsat 1 v5 gn 8 126000.2
GSVIVT01024071001.2
AT2G16630.2
Aqcoe5G117200.2
P.equestris XP 020587277. 2
Oropetium 25700A.2
Aco019419.2
E
P.equestris XP 020582329. 2
GSMUA Achr8T28070.2
GSMUA Achr3T25240.2
GSMUA Achr3T15900.2
Aco003923.2
Spipo2G0081100.2
AYMT 2010734 Eucalyptus l.2
P.equestris XP 020593305. 2
Zosma6g00520.2
Aqcoe3G376000.2
GSVIVT01036543001.2
AmTr v1.0 00047.2
Gorai.001G206700.2
Eucgr.J01410.2
Gorai.001G206900.2
Lus10034365.2
AT5G15780.2
Lsat 1 v5 gn 4 173421.2
Lsat 1 v5 gn 2 90181.2
F
Bradi3g15970.1
Lus10039078.1
Aqcoe4G128000.1
AmTr v1.0 047.45.1
Aco012434.1
P.equestris XP 020591182. 1
Zosma114g00880.1
G
Oropetium 03649A.3
LOC Os05g45460.3
Bradi2g18920.3
Oropetium 12372A.3
LOC Os05g45480.3
Oropetium 03650A.3
Bradi2g18910.3
Oropetium 12371A.3
LOC Os01g52650.3
Bradi2g48670.3
Aco005612.3
Aco003161.1
Lsat 1 v5 gn 4 53840.3
Lsat 1 v5 gn 7 21560.3
GSVIVT01007286001.3
AmTr v1.0 068.122.1
Gorai.010G154300.3
Gorai.009G168900.3
Eucgr.E03269.3
Lus10015434.3
Aqcoe4G097400.3
AT2G34700.3
AT2G33790.3
AT1G28290.3
H
GSVIVT01032773001.1
AT5G05500.3
Gorai.011G291100.1
Eucgr.G03079.1
Lsat 1 v5 gn 2 124301.1
Lus10040948.1
Lus10017440.1
Aqcoe1G153900.3
P.equestris XP 020583201. 1
AmTr v1.0 153.4.1
Zosma175g00470.1
Zosma175g00410.1
Zosma175g00430.1
Aco018698.1
LOC Os01g07060.3
Oropetium 07184A.3
I
AT3G09925.1
Gorai.002G160400.1
Lus10023887.1
Aqcoe1G120900.1
P.equestris XP 020587648.
AmTr v1.0 019.72.1
Oropetium 22028A.1
Zosma78g00260.1
J
Bradi3g21030.2
LOC Os10g05750.2
Bradi1g68390.2
LOC Os03g14130.2
Bradi3g21010.2
Oropetium 23782A.2
Oropetium 25797A.2
Bradi3g21000.2
LOC Os10g05790.2
LOC Os10g05950.2
Bradi5g04630.2
LOC Os10g05820.2
Bradi1g63370.2
LOC Os10g05970.2
LOC Os03g14140.2
Bradi1g68400.2
GSMUA Achr5T11390.2
GSMUA AchrUnT21190.2
P.equestris XP 020594514. 2
AmTr v1.0 041.169.2
Spipo16G0020300.2
Aqcoe5G210700.2
Aqcoe5G210800.2
Lsat 1 v5 gn 3 45680.2
Eucgr.L01869.2
GSVIVT01024076001.2
GSVIVT01024081001.2
Eucgr.I01377.2
Gorai.002G193500.2
Gorai.012G153300.2
Aco019424.2
Aqcoe1G439100.2
P.equestris XP 020573394.2
Bradi5g09480.2
LOC Os02g33170.2
Oropetium 11363A.2
Bradi3g45020.2
